# Laboratory-to-Industrial Translation in Plant-Derived Bioproduct Valorization: A Critical Integrative Review and Proposed Framework for Route-Level Evidence Assessment

**DOI:** 10.3390/molecules31203689

**Published:** 2026-10-15

**Authors:** Hyo Jun Won

**Affiliations:** Independent Researcher, Seoul 06300, Republic of Korea; hjwon7943@ust.ac.kr

**Keywords:** agri-food byproducts, plant-derived bioproducts, scale-up, product specifications, functional equivalence, evidence assessment, techno-economic assessment, life cycle assessment

## Abstract

Gross extraction yield alone cannot establish product conformity or broader economic and environmental performance. This critical integrative review examines evidence linking laboratory findings to industrially relevant claims for plant-derived bioproduct valorization. It synthesizes seven evidence streams and proposes specification-adjusted route accounting, connecting route identity, output states, and claim-specific requirements. Selected elements were illustrated retrospectively using 17 purposively selected evidence packages. Artemisinin retention decreased from 90.0% after maceration to 37.3% after crystallization on the same initial artemisinin basis. Higher grapefruit-pectin yield accompanied changes in product properties without establishing functional equivalence. Red-grape-pomace economic preference depended on price assumptions. For microencapsulated pomegranate-peel extract powder, the highest deterministic net present value and highest probability of a positive net present value occurred in different modeled scale scenarios. Its separately scoped life cycle assessment identified freeze-drying as a hotspot without establishing comparative environmental improvement. No selected package provided verifiable observed-displacement evidence at the documented access level. The framework distinguishes supported conclusions from unresolved requirements; the cases illustrate its logic rather than validate its performance. Purposive selection, limited source access, and single-author assessment constrain interpretation. Independent and prospective evaluation is needed before reproducibility or decision benefits can be established.

## 1. Introduction

Agri-food processing byproducts and other plant-derived resources can supply bioactive compounds and functional ingredients [1,2,3]. Laboratory extraction studies provide useful measures of recoverable material, composition, and process response [4,5]. These measures answer different questions: gross extraction yield describes recovered extract mass on a stated feed basis, concentration describes a constituent within an analyzed material, and target recovery relates recovered target mass to its initial amount. None alone establishes that a final product meets a specification or supports a broader economic or environmental claim. Feedstock source, tissue, handling, moisture, composition, and storage conditions also affect how such results can be interpreted and compared [2,6,7,8].

Chemical characterization connects these process measurements to the product being evaluated. Linking successive recoveries of a named constituent requires continuity of target identity and compatible mass bases; reduced recovery alone does not identify degradation as the cause of a loss. Purified compounds require an explicit basis for assay or purity [9,10,11], whereas standardized extracts require compositional criteria appropriate to their intended use [12,13]. For polysaccharide ingredients such as pectin, composition and structural or physicochemical properties must be related to performance under defined conditions [14,15]. The relevant requirements therefore depend on product grade and use, rather than a universal preference for higher purity, molecular weight, or yield. Separation, concentration, drying, and formulation can alter target retention, composition, final mass, and product properties [16,17]. Analytical results must consequently identify the material state and measurement basis they represent. Characterization, conformity with predeclared criteria, and functional equivalence to a named comparator remain distinct assessments.

Laboratory-to-industrial translation introduces an additional question: which evidence can be transferred across changes in process configuration and scale? Laboratory measurements, pilot observations, modeled industrial configurations, and operating industrial systems provide different forms of evidence; their connection requires explicit treatment of changes in feed, operations, product state, utilities, and system boundary [18,19]. Product grade and function also affect the interpretation of price assumptions and substitution relationships [20]. Techno-economic assessment (TEA) and life cycle assessment (LCA) address different outcomes even when they share a process model. In environmental comparisons, a byproduct’s existing use and burden origin, the counterfactual, and the treatment of foregone functions remain relevant [21,22,23]. Likewise, circularity indicators depend on their stated scope and accounting choices; increased circulation alone does not establish environmental improvement [24,25,26,27,28]. These distinctions motivate explicit links between chemical and product evidence and the assumptions used in economic, environmental, and substitution assessments.

Recent reviews address material readiness, green recovery and profiling, integrated ingredient processing, and valorization across biomass and waste domains [29,30,31,32,33,34,35,36,37]. Functionality-driven design, quality-sensitive circularity assessment, and integrated economic–environmental assessment already address relationships among products, processes, and their consequences [15,38,39,40]. The question pursued here is how heterogeneous records support a particular conclusion about a defined feedstock–process–product configuration at a stated scale. Evidence essential to that conclusion—termed load-bearing evidence in this review—must refer to compatible conditions or be reconciled through a documented, justified transformation. The contribution is an explicit structure for examining those connections and identifying which unresolved requirements limit a particular claim while retaining separately supported findings.

The scope comprises selected non-energy plant-derived products, including agri-food byproduct routes and other plant-derived systems with contrasting chemical, product, and process characteristics. Four research questions guide the review:

RQ1. What feedstock, chemical-characterization, process, product, and comparison information is needed to assess a specified route-level claim? Definitions and requirements are set out in Section 2.2, Section 2.3, Section 2.4 and Section 2.5 and Section 3, and the cross-stream synthesis is presented in Section 4.1.

RQ2. Which differences require alignment or explicit reconciliation when linking laboratory measurements, pilot observations, modeled scale-up, and industrial evidence? Linkage rules are described in Section 2.2, Section 2.3, Section 3.2 and Section 3.3 and illustrated in Section 4.4, Section 4.5 and Section 4.6.

RQ3. What additional evidence is needed to move from recovery measurements to claims of specification-conforming output, benchmark-equivalent service, or substitution? The relevant criteria are defined in Section 2.4, Section 2.5 and Section 3 and illustrated in Section 4.3, Section 4.4 and Section 4.5.

RQ4. How do selected cases illustrate the distinction between supported conclusions and unresolved evidence requirements? Case selection and coding are described in Section 2.1, Section 2.2 and Section 2.3, with the demonstration findings reported in Section 4.2, Section 4.3, Section 4.4, Section 4.5, Section 4.6 and Section 4.7.

These questions concern evidence interpretation, not prediction of industrial performance. Supplementary Table S5 defines the manuscript-specific terms and principal abbreviations.

This critical integrative review synthesizes seven evidence streams spanning feedstock identity, recovery and profiling, downstream processing and formulation, product quality and readiness, circularity and substitution, TEA, and life-cycle and counterfactual assessment. It proposes specification-adjusted route accounting to connect route-identification fields, output states, and claim-specific evidence requirements and stopping rules. Selected elements are illustrated retrospectively using 17 purposively selected evidence packages covering molecular-identity, composition-constrained, and function-constrained products. The assessment preserves technical and model-conditional findings within their documented scope and distinguishes modeled substitution potential from empirically observed displacement. The cases provide a nonrepresentative demonstration of the proposed logic; they do not validate the framework. Detailed selection procedures, analytical units, and implementation limits are reported in Section 2, Section 3 and Section 5.5.

## 2. Review Design and Evidence Architecture

### 2.1. Review Design, Source Identification, and Criterion-Based Case Selection

This critical integrative review combines synthesis across seven evidence streams, a proposed framework, and retrospective illustration of selected framework elements using author-curated cases. Specification-adjusted route accounting links route-identification fields, successive output states, and claim-specific evidence requirements and stopping rules. Supplementary Table S2 translates these relationships into proposed reporting items. The framework, checklist organization, and operational rules are author proposals informed by the literature; case application illustrates their logic rather than validating them. The review supports conceptual integration and clarification of claim scope, not exhaustive enumeration, meta-analysis, field-wide frequency estimates, or formal risk-of-bias assessment [41,42,43]. Sources could contribute to synthesis, case evidence, or methodological and contextual discussion. These roles could overlap, but synthesis or contextual use alone did not establish eligibility as an evidence package.

The methodological comparison in Section 4.8 is purposive and qualitative. It covers literature-review methodology, product specification and functionality, circularity and recycling quality, substitution, scale-up, TEA, LCA, and integrated assessment. Indicators, conceptual frameworks, reporting guidance, standards, and integrated models are compared by purpose, unit of analysis, principal inputs and outputs, linkage requirements, and scope. Established methodological principles are distinguished from this review’s proposed route-identification fields, coding categories, and stopping rules. The comparison examines evidence interpretation and linkage; it is not an exhaustive benchmark, test of computational performance or standards compliance, or demonstration of superiority.

The seven streams were feedstock identity; recovery and profiling; downstream processing and formulation; product quality and readiness; circularity and substitution; techno-economic assessment (TEA); and life-cycle and counterfactual assessment. The coverage criterion for each stream was at least two anchor publications not authored by the present author and one source addressing its principal contested boundary. This authorship criterion did not establish statistical independence or exclude overlap in the underlying studies. The author’s earlier reviews provided continuity with previous syntheses and were not counted as anchors. Eligibility for the case set was assessed separately.

The targeted-audit backbone and scope-challenge candidates were provisionally assembled before the database searches on 11–12 August 2026. Candidate records in Supplementary Workbook S1 give verification dates of 8 August for most entries and 13 August for CC-02 and FC-02; these dates do not necessarily indicate initial discovery. Source-specific verification and targeted checks for missing route information continued through 13 August. Package retention required (i) a clearly defined non-energy plant-derived product, (ii) sufficient feedstock, process, and product information to identify at least one configuration, (iii) at least one claim-relevant evidence link, and (iv) a publicly verifiable source record. The inclusion criteria did not require demonstrated industrial operation, a complete route assessment, or both TEA and LCA. Partial evidence or a source-defined modeled scenario could support inclusion while limiting the claims supported. Recorded exclusions concerned product scope or insufficient route definition; one conference poster/proceedings record lacked sufficient primary route-level evidence.

The candidate-selection sheet documents 30 entries: 17 linked to retained packages (CAN-001–CAN-017), five contextual or methodological sources (CAN-018–CAN-022), and eight excluded candidates (CAN-023–CAN-030). The contextual sources informed baseline fate, existing uses, preparative chromatography, scale reconciliation, and substitution; they were neither excluded candidates nor evidence packages. Figure S4 summarizes these dispositions and the recorded exclusion reasons, separately from the subsequent database coverage searches. Source keys, package identifiers where applicable, dispositions, and rationales remain in Supplementary Workbook S1. The records do not provide complete candidate-specific initial-discovery histories; the documented verification dates therefore cannot reconstruct that process. These records describe a purposive candidate audit, not screening of the database result sets. The 17 packages illustrate contrasting evidence structures; completeness of candidate capture and freedom from selection bias cannot be inferred. Core and boundary-condition labels denote analytical roles, not representativeness, evidence quality, or validation status.

Database searches were used to check predefined stream coverage and examine documented route-evidence gaps. Scopus was searched on 11 August 2026 using TITLE-ABS; Web of Science Core Collection was searched on 12 August using Title or Abstract (TI/AB; all editions), and the main PubMed searches on the same date used Title/Abstract [tiab]. Where available, no user-defined filters were applied for date, language, document type, subject area, species, access status, or full-text availability. The recorded result counts were 63,465 for Scopus, 48,706 for Web of Science, and 19,592 for PubMed. These are platform-specific search outputs, not counts of screened publications or full-text assessments. The result sets were not pooled or used as an eligibility corpus. Exact search strings, execution details, coverage checks, and export scopes are reported in Supplementary Table S1.

Complete database result sets were not exported. Supplementary Table S1 records targeted exports comprising the Scopus relevance-ranked Top-50, Mandatory-16, and Desirable-8 lists; the Web of Science Mandatory-13 Full Record list; and the PubMed Mandatory-7 and Desirable-5 lists and Search History CSV. Potentially overlapping lists were not summed and no cross-database duplicate count or unique-record count after deduplication was calculated. DOI, title, and publication-version checks concerned source identity, not deduplication of a pooled corpus.

The source-access tally comprised 38 predesignated audit sources (SRC-001–SRC-038 in Supplementary Workbook S1): 15 checked in full text, one checked in official publisher-hosted unedited full text [44], and 22 checked through official abstracts, metadata, or other accessible records. SRC-039–SRC-046 document eight additional sources used only for the exclusion audit. Other cited references were outside this tally. The 38 sources, 30 candidate entries, 17 retained packages, and 45 registered analytical records refer to different counting units.

Bibliographic identity and publication status were checked against official publisher or Version of Record metadata. Claim scope, numerical conditions, system boundaries, and evidence locations were examined only to the extent supported by the accessible material, which included final published full texts, an accepted manuscript, an official publisher-hosted unedited full text, and official abstracts or metadata records. Metadata checks were distinguished from full-text verification, and manuscript versions were identified separately. Source-access rechecks for CC-01 and MI-01 were recorded on 26 August 2026. For CC-02, the source register records verification of the publisher-hosted unedited version on 13 August 2026, and the Monte Carlo extraction records document a separate content check on 26 August 2026. Supplementary Workbook S1 records access levels and interpretive limits. Targeted reference-list and cited-by checks addressed specific claim–source or route-evidence gaps; no exhaustive citation-network search was undertaken.

NR was assigned only when a sufficiently complete accessible record supported a judgment that information required for the evaluated claim path was not reported. Omission from an abstract or metadata record alone did not establish nonreporting. When limited access prevented verification of a claim-relevant field, that field was coded P and the access limitation was recorded in the rationale. Coding was field-specific; limited access did not require assigning P to every field from a source. Access to a full text does not, by itself, establish nonreporting for every field; the judgment requires examination of the material relevant to that field. An explicit source statement that a required measurement or assessment was not reported can support a nonreporting judgment for that item within the stated scope; it does not establish absence of the underlying phenomenon.

### 2.2. Evidence Units and Route-Linkage Rules

Evidence was organized as source publications, evidence packages, and nested analytical records. Publications provided the source evidence. A package could comprise a single publication containing one route or a set of related comparisons, or multiple publications linked by a documented common route. A publication could therefore contribute more than one configuration, scenario, or comparison record. A route family described a shared feedstock–product objective and was not a counted analytical unit. Source identifiers connect the supporting publications to package and analytical record identifiers in Supplementary Workbook S1; methodological or contextual use alone does not establish membership in a package.

When evidence from multiple publications was linked, a common route identity required compatible feedstock conditions, unit operations, product definitions, and scale or model bases, or a documented reconciliation of differences relevant to the claim. A shared plant species or target compound was insufficient. Grouping records within a package did not itself establish compatibility or permit their results to be combined. Each claim-relevant linkage was assessed separately, and any reconciliation was limited to its documented conversion or assumption.

A route configuration was identified by feedstock condition, unit-operation sequence, scale, final product state or grade, utility basis, and residual fate. The functional unit, comparator, and counterfactual were additional linkage fields when required by the claim. A reference or parent configuration could itself be modeled. A configuration variant denotes an experimental or operational alternative, whereas a modeled scenario specifies changes in source-defined model conditions. Stage-specific records and records for different botanical extracts require separate interpretation: a downstream purification stage is not necessarily an alternative route, and a different extract does not establish an equivalent product. Parent–child links in the register indicate how records are organized; their scientific meaning must be established from the associated conditions and source evidence.

A within-source comparison record represents a contrast between two separately identified configurations. For example, FC-04-C01 and FC-04-C02 identify conventional and ultrasound-assisted heating extraction, respectively, whereas FC-04-C03 records their comparison. The third record does not represent another extraction route or an independent replicate. The register label “paired comparison” does not, by itself, imply a paired statistical design or a paired-sample test.

Laboratory-to-industrial translation was examined as a question of evidence linkage across scales. Laboratory measurements, pilot-scale observations, modeled scale-up scenarios, and evidence from operating industrial systems were interpreted at their documented levels of inference. Cross-scale linkage required alignment or explicit reconciliation of the relevant feedstock, process, product, utility, residual-fate, and scale or model bases. The presence of records at different scales did not establish a longitudinal progression of the same route.

A common route-level claim required explicit evidence for its essential fields, or sufficient partial evidence for a bounded conclusion when no stopping condition applied. Every essential cross-source or cross-basis linkage had to be aligned or explicitly reconciled. An R code required a traceable record of the original and converted bases, the transformation or assumption, and the affected claim. Unresolved differences in feedstock basis, scale, unit operations, product grade, utilities, residual fate, functional unit, comparator, counterfactual, geography, time, or impact method stopped the affected path when essential to the claim. Reference-substance prices were therefore not assigned to crude extracts, laboratory and industrial drying were not linked without reconciliation, and beneficial side-stream uses were not assigned a default zero burden.

The 17 packages contain 45 registered analytical records. This total describes the structure of the selected evidence; it is not a count of independent routes, studies, experimental replicates, or validation samples. Configuration- or scenario-specific inference requires distinct reported conditions and outcomes, an identifiable source location, and a traceable connection to the relevant record. Where the accessible evidence did not distinguish those outcomes, interpretation remained at the package level. Conformity, economic, or environmental findings for one configuration cannot be transferred to another solely because both belong to the same package. The number of records in a package is not a measure of evidence strength.

The evidence package remained the organizing unit of the retrospective assessment, and the package–field cell was the unit of the ten-field coding matrix. Supplementary Figure S1 summarizes the case-set structure. The Package Register, Configuration Index, and Unit of Inference sheets in Supplementary Workbook S1 provide identifiers, organizational links, source locators, and permitted and prohibited inferences. These records support traceability but do not establish complete source verification or independent reproducibility. The retrospective assessment did not implement A01–A08 comprehensively for every configuration and claim. The reported ceilings therefore remain structured qualitative package-level judgments, informed by configuration-specific evidence where separable, rather than a complete set of reproducible configuration-level calculations.

### 2.3. Coding, Provenance, Compatibility, and Internal Consistency

The retrospective matrix comprised 170 package–field decision cells (17 evidence packages × 10 evidence fields), each representing one field within one package. Availability and completeness concern the information available for that field; provenance identifies the source or derivation of an evidence record; and compatibility concerns a specific linkage supporting a claim. These dimensions have different units and potentially different evaluable denominators. The 170 cells are neither independent observations nor a pooled count of all judgments. The matrix summarizes package-level evidence; it does not separately assess all 45 registered analytical records or constitute full application of the proposed 42-item checklist in Supplementary Table S2.

Availability and completeness were expressed as E (explicit), P (partial or bounded), NR (required for the evaluated claim path but not reported), and NA (not applicable). E denotes explicit evidence for the assessed field, which may concern route identity, recovery basis, specification, conformity or equivalence assessment, a model result, or an empirical displacement relationship. It does not indicate favorable performance or a passing conformity decision. A documented specification failure can therefore be explicit without supporting conformity, and an explicit LCA result can identify a hotspot without establishing comparative environmental improvement.

P can reflect incomplete conditions or denominators, support for only part of a requirement, or limited source access. Its rationale should identify the limitation and any narrower conclusion directly supported by accessible evidence. P does not necessarily indicate partial fulfillment: an unverified requirement cannot support a claim that depends on it. NR requires applicability and an accessible record sufficient to support a nonreporting judgment, as specified in Section 2.1; it does not establish absence of the underlying phenomenon. NA identifies a requirement outside the defined claim scope and is not justified by missing information alone. No unreported load-bearing parameter was imputed.

Provenance and derivation status distinguish source-reported information (SR), source-reported modeled outputs (MS), author calculations from traceable source-reported inputs (SR/AD), and author derivations introducing additional assumptions or constructions (AD). SR includes observed measurements and directly reported study conditions, including model inputs or scenario settings; these are distinguished from MS outputs of a source-defined model. SR/AD requires a traceable calculation record. AD includes author-constructed scenarios, classifications, or other derivations involving author-specified assumptions. Inputs, derived results, and interpretive rationales should remain distinguishable. These labels describe provenance, not evidence quality, and assigning an evidence-status code does not change the provenance of the underlying information.

Linkage compatibility distinguishes aligned (A), explicitly reconciled (R), and incompatible (I) records. The relevant records and intended claim must first be identified. A applies when the conditions essential to that linkage are aligned. R requires a traceable reconciliation specifying the original and converted bases, the transformation or assumption, and its limits. I identifies a documented mismatch in an essential basis that remains unresolved. Membership in the same publication or package does not establish A, and R does not permit an unreported load-bearing value to be invented. Insufficient information to determine compatibility is distinct from demonstrated incompatibility. An invoked linkage that cannot be evaluated should be identified as unresolved in the rationale; it is not assigned I solely because information is missing or NL merely because compatibility could not be established.

NL identifies an eligible evidence record for which no cross-source or cross-basis linkage is invoked in the assessed claim. It is not an availability code or a certification that the record could be linked to other evidence. A blank compatibility entry denotes the absence of an eligible evidence record to which a compatibility classification can be assigned. A context or gap-rationale row does not become eligible evidence merely because it appears in the log. NL, a blank entry, NR, and NA therefore have different meanings and are not interchangeable.

The following hypothetical examples illustrate the proposed rules; they are not additional coded results. Wet- and dry-mass quantities could be linked as R when measured moisture for the relevant material, a documented conversion, and other claim-relevant conditions establish a common basis. Unit conversion alone does not reconcile differences in product grade, scale, or system boundary. The I code would apply to a documented material incompatibility that remains unresolved in a required linkage; missing information would leave compatibility unverified. A calculation based on an analyst-selected realization assumption would be AD rather than a wholly source-reported model output, and the assumption would require an explicit label and rationale.

The Cell Decision Master in Supplementary Workbook S1 records the 170 package–field availability and completeness judgments. The Evidence Record Log connects supporting evidence and explanatory context to source locators, provenance, compatibility, and calculations. The Availability, Provenance, and Compatibility Matrix sheets summarize the associated records at the package–field level. The Claim Path Stop Audit contains 102 package–claim records, including claim-specific conclusions, stopping reasons, and qualitative consideration of auxiliary requirements, as described in Section 2.5. These record counts describe different units and are not interchangeable. Figure-based summaries are presented in Section 4.7.

During revision, availability and completeness codes were tabulated descriptively by recorded access to each package’s route evidence source (Table S6). Source identifiers were linked across the Configuration Index, Cell Decision Master, Evidence Record Log, and Claim Path Stop Audit to the Source Register in Supplementary Workbook S1. Sources recorded as full-text checked or official publisher-hosted unedited full text formed the full-text stratum, with version limits retained. The other stratum comprised abstract- or metadata-based access. General contextual and exclusion-audit sources were not used to classify packages. Each package had one route-source identifier, so no mixed-access stratum was required. Each package contributed one finalized E, P, NR, or NA code per field; applicable counts equaled stratum size minus NA. The strata describe recorded source access, whereas each field code reflects the sufficiency of its documented evidence and review scope. Accordingly, MI-02, MI-03, CC-03, and FC-04 retain their recorded full-text stratum but have F10 = P because sufficient F10 review coverage could not be established. The tabulation does not estimate an effect of access or constitute independent validation.

Within the proposed framework, each package–field summary should identify the claim-relevant output and supporting records. A provenance summary refers to that output rather than to every associated input, scenario description, or contextual statement. When configurations or records differ, the summary should preserve conditions and exceptions relevant to the claim. One favorable record cannot override an unresolved limitation in another record required for the same claim. Context-only and gap-rationale records do not supply positive evidence for the field, and provenance labels are not averaged or combined into a score. Evidence-level records and claim-specific rationales remain necessary for interpreting each summary code.

For the original submission, source selection, data extraction, classification, and appraisal were performed by the sole author. The author then conducted a structured second-pass check of source locators, units, denominators, package and configuration identifiers, author-derived calculations, and reported values across the manuscript, tables, figures, and workbook. This was an internal consistency check by the same author; it did not involve independent duplicate source selection or data extraction, inter-rater calibration, or external validation.

### 2.4. Specification Evidence and Claim-Relevant Appraisal

Product-related evidence was appraised across four distinct product-evidence states: (1) a characterized endpoint; (2) a predeclared acceptance specification; (3) demonstrated conformity of the assessed batch or product stream with that specification; and (4) repeat-batch conformity or documented commercial release. These states organize different kinds of product evidence; they do not form a universal maturity ladder for the six claim classes. A product specification defines the acceptance requirements, whereas conformity concerns whether the assessed product meets those requirements. Neither concept is interchangeable with gross extraction yield, target-specific recovery, or functional equivalence, as distinguished in Section 3.1 and Section 3.2.

Within the proposed framework, a product specification identifies the intended use, claimed grade, product state, relevant attributes, measurement methods and conditions, and acceptance limits or decision rules. Its source and applicability should be stated, distinguishing regulatory or recognized specifications from purchaser, manufacturer, or research-defined criteria. A compositional fingerprint requires an explicit comparison or acceptance rule. Predeclaration establishes the timing of a criterion; it does not, by itself, establish that the criterion is appropriate for the intended use.

Here, predeclared means established before the relevant batch results were evaluated or interpreted for conformity, not necessarily preregistered before experimentation. An external standard’s existence does not establish its adoption before outcome evaluation. If the accessible record does not establish that timing, predeclaration remains unverified. A post hoc comparison with literature values or a commercial product can inform characterization but cannot retrospectively establish a release specification. Conformity with one attribute does not establish conformity with all applicable acceptance criteria.

Analytical replicates are not independent production batches, and single-batch conformity does not establish repeat-batch conformity or routine release. Repeat-batch conformity and documented commercial release remain distinct evidence types within the fourth state. A release record supports only its documented product, batch, and criteria; sales alone do not establish conformity, and release does not establish observed displacement.

Supplementary Workbook S1 distinguishes source-type-specific reference questions in the Critical Appraisal Rules sheet from descriptive source-role checks in the Source Appraisal sheet. The latter document source use and access limitations, not completed domain-by-domain ratings. Field and claim records document reporting completeness and applicability. Neither these checks nor descriptive concern labels constitute formal risk-of-bias or certainty ratings or raise claim ceilings.

An NR or I code, or explicit failure of an acceptance or equivalence criterion required for the evaluated claim, stopped the affected path. Failure to establish conformity because of missing or inaccessible evidence was distinguished from demonstrated nonconformity. P permitted only a directly supported bounded or conditional conclusion when no stopping condition applied; an unverified element could not support a claim that depended on it. NA did not constitute a reporting gap for that path. The architecture is a review-specific audit aid, not a validated risk-of-bias tool, GRADE assessment, certification scheme, reporting standard, or certainty instrument.

### 2.5. Claim Paths, Accounting Bases, and Uncertainty Treatment

Six claim classes were evaluated separately: technical realization, physical resource productivity, economic plausibility, environmental improvement, modeled substitution potential, and observed displacement (Table 1). They define distinct claim paths rather than a maturity ladder. A defined feedstock–process–product route and a traceable, claim-appropriate technical basis were shared prerequisites. Empirical realization was required only when claimed; purely modeled TEA or LCA findings remained model-conditional evidence, not evidence of realized routes. Economic plausibility and environmental improvement were evaluated in parallel, and support for one did not establish the other.

Within the proposed framework, the intended claim, product, and route boundary are specified before applicability is assessed. Requirements are then classified as essential, conditionally essential, or not required. Conditional requirements become necessary when a comparison depends on product grade, function, use level, or another stated condition. The rationale should distinguish requirements outside the assessment scope from applicable requirements lacking evidence. Applicability concerns the question evaluated in this review, not whether each source study intended to support all six classes. Observed displacement was examined across all 17 packages as a cross-cutting review question, not imposed as a universal reporting obligation.

Comparative claims required an explicit comparator and a common comparison basis; claims depending on matched function or use additionally required relevant product or service equivalence. Each conclusion was limited by the first unsupported or incompatible essential link, including required-but-unreported evidence or a failed claim-required criterion. The modeled-substitution implementation proposed here required an explicit comparator, benchmark-equivalent service, a defined supply and demand context, and stated post-cap realization assumptions.

Table 1 summarizes the claim-specific assessment rules proposed in this review. It distinguishes evidence needed for the intended claim from additional conditions for broader interpretations and identifies the scope of the conclusion supported by the reporting and linkage record.

The proposed framework also identifies eight auxiliary requirements that are not represented as separate fields in Section 4.7: the final output state and accounting basis (A01); the named physical input and its quantity (A02); consistency between output and input denominators (A03); the comparator, functional unit, or incumbent appropriate to the claim (A04); baseline handling or use and the counterfactual (A05); supply available for the evaluated use and addressable demand (A06); post-cap realization assumptions (A07); and empirical evidence of incumbent displacement (A08). These requirements specify additional information needed for particular claims; they are not quality scores or eight additional fields completely evaluated in the retrospective matrix.

Their applicability depends on the intended claim. A01–A03 are central to a physical resource-productivity ratio. A04 identifies the comparator and comparison basis appropriate to the specified claim; a functional unit and a named incumbent serve different purposes and are not interchangeable. A05 applies when baseline fate or an alternative use affects the comparison. A06–A07 concern modeled substitution potential, whereas A08 concerns observed displacement. Product conformity and service equivalence are required only where necessary to support the specified claim. The retrospective assessment defined these requirements and considered some of them qualitatively in claim rationales, but it did not complete a separate A01–A08 applicability, evidence-status, and linkage record for every package–claim combination.

TEA results were retained at their reported unit of inference. Interpretation depended, as applicable, on route-matched throughput and utilization, currency and price year, CAPEX/OPEX boundary, coproduct and residual treatment, quality-control and rejection assumptions, and the decision metric. Missing essential information limited the claim to its last supported economic statement. A source-reported deterministic estimate could remain interpretable within its assumptions without establishing robustness, methodological completeness, or compliance with TEA guidance. Stability across conditions or robustness required relevant sensitivity or uncertainty evidence. Deterministic estimates and source-reported probabilistic results were retained as distinct outputs rather than merged into one scenario ranking.

Accounting fields were grouped into feedstock bases, output states, physical resource inputs, and residual or co-product outputs. Physical resource productivity was defined only for a named output state paired with a matched physical input over the same route, mass basis, and system boundary; the numerator was not advanced from stage recovery to specification-conforming output or benchmark-equivalent service without the corresponding evidence. Concentration was not treated as recovery, gross solvent circulation was not treated as fresh-solvent use, and land productivity was considered defensible only when upstream attribution to the audited route was explicit. No package-level physical resource-productivity ratio was calculated in the retrospective case assessment. A ratio was not inferred from gross recovery or the presence of an inventory without an established, route-aligned output numerator and attributable physical-input denominator. Non-calculation by this review does not establish that the source omitted the required quantities or that the route had poor resource productivity. An applicable but unevaluated claim is distinct from NR for a specific required evidence field and from NA for a claim outside the documented scope.

Multi-output systems were handled through a review-specific sequence for internal route consistency, not a universal allocation hierarchy. Subdivision was preferred when operations and inventories were separable; otherwise, the source-reported system-expansion or allocation method was accepted only when explicit and justified. Substituted product and ratio, or the allocation basis, had to be reported. Shared flows and burdens were partitioned or retained within the complete product basket so that avoided baseline fate and displaced function were not credited twice. Mutually exclusive scenarios were not summed or treated as independent evidence [20,23]. Under the proposed framework, product-specific productivity ratios should be reported only when inputs can be assigned consistently; otherwise, the complete product basket and shared input profile should be retained.

Uncertainty was represented only by source-reported ranges, source-reported probabilistic outputs, and explicit scenarios. Author-constructed scenarios were labeled; no new probabilistic analysis was performed. The normalized cumulative-recovery calculation for MI-02 was a point calculation without quantified uncertainty. Sequential recoveries were multiplied only when their denominators were conditionally nested and target identity was preserved. Service adjustment required matched tests and defensible proportionality. Evidence limitations followed Section 2.1 and Section 2.3: partial, inaccessible, and unreported evidence were distinguished, and unresolved essential requirements, confirmed incompatible bases, or failed required criteria limited the affected claim. Preference was described as stable only within a reported range or scenario set. A quantified switching point or bounded interval supported conditional preference, not a route-independent ranking. No unreported distributions, cross-case averages, meta-analysis, or composite score were introduced. Supplementary Workbook S1 records transformations and denominator risks.

Equations (1)–(9) are proposed deterministic accounting relations and operational definitions, not calibrated predictive models. Their use requires matched units, boundaries, and denominator structures, with explicit route assumptions. Retrospective numerical application was limited to normalized cumulative target recovery in MI-02, using Equation (3), source-reported conditional recovery fractions, and an initial target amount of 100%. It neither determined absolute target mass through Equations (1)–(3) nor implemented the full chain. The other equations served as accounting definitions and claim-eligibility boundaries and were not numerically applied. The 2.41-fold retention comparison and price-bounded switching interval were separate case-specific derivations. Equation (1) was not retrospectively calculated where generated mass, route accessibility, and incoming-feed acceptance were not established on a common basis.

## 3. Proposed Conceptual Framework for Specification-Adjusted Route Accounting

This section presents the proposed conceptual framework, including product archetypes, accounting definitions, and claim-specific evidence requirements. The equations are presented together to distinguish the denominators, output states, and comparison bases used across claims. As described in Section 2.5, retrospective numerical application was limited to normalized cumulative target recovery in MI-02 using Equation (3), source-reported conditional recovery fractions, and preserved target identity. The remaining equations define proposed accounting relationships and evidence requirements; they were not numerically applied in these cases. Their inclusion does not establish predictive performance or empirical validation of the full accounting chain.

### 3.1. Product Archetypes and Specification Anchors

The proposed framework distinguishes three product archetypes according to the attributes that define the claimed final product, grade, and intended use. Molecular-identity (MI) products are defined primarily by molecular identity and quantitative purity or assay; composition-constrained (CC) products by specified compositional ranges or profiles; and function-constrained (FC) products by performance under defined conditions. These archetypes organize claim-relevant evidence requirements. They are not mutually exclusive descriptions of every product attribute, an exhaustive taxonomy, quality rankings, or regulatory classifications. Feedstock identity or the measurement of a single marker or activity does not, by itself, determine the archetype.

MI products, including purified compounds and reference substances, may require evidence of identity, assay or purity, stereochemical control, relevant impurities and degradation products, residual solvents, contaminants, and traceability, depending on the claimed grade and intended use [9,10]. For reference materials, purity assignment should identify the measurement basis and associated uncertainty and may integrate mass balance, quantitative nuclear magnetic resonance, thermal methods, and other direct assays [11]. CC products, such as standardized botanical extracts, require predeclared compositional acceptance criteria, including marker ranges or fingerprints with defined decision rules, together with claim-relevant contaminant, stability, and batch-conformity evidence [12,13]. FC products, including pectin and dietary-fiber or polysaccharide fractions, require predeclared functional acceptance criteria assessed under defined conditions; identity, composition, stability, and safety remain relevant where required by the intended use [14,15,20]. The cited standards and guidance serve as intended-use- and grade-specific specification anchors, not universal release criteria or evidence that an assessed product is certified. In particular, specifications issued by the Joint FAO/WHO Expert Committee on Food Additives (JECFA) for pectin apply here only when food-additive-grade pectin is claimed.

For future application, a primary archetype should be assigned according to the attributes that define the claimed final product, with additional identity, composition, or functional requirements recorded when relevant to the claim. An FC pectin product may therefore also require compositional limits, and a CC extract used as a functional replacement requires comparative performance evidence. Quantifying a marker does not make an extract an MI product, and reporting an antioxidant response does not establish that an extract meets an FC specification. The primary label organizes the assessment; it does not exempt a product from other applicable requirements or create additional packages for counting purposes.

Functional equivalence is a claim-specific comparison requiring a named reference product, a defined service and use context, matched or explicitly reconciled test conditions, and a stated criterion for equivalent performance at the relevant use levels. Similar composition, viscosity, or another isolated property—and the absence of a statistically significant difference—does not, by itself, establish equivalence. A standardized extract should not inherit the price basis or substitution credit of a purified compound without evidence supporting the claimed grade and comparison. Likewise, a recovered fiber is not equivalent to a commercial fiber solely because both have the same nominal product label. Satisfaction of one favorable criterion does not establish conformity with all applicable specifications, and evidence for one defined service does not establish efficacy, safety, or equivalence in other applications.

### 3.2. From Generated Feed to Specification-Conforming Output and Benchmark-Equivalent Service

Throughout Section 3, t denotes a target compound or constituent, p a final product stream, s a defined service or application, j a unit operation, i a physical resource input, and h an environmental impact category.

For readability, route-configuration, scale, and temporal indices are suppressed in Equations (1)–(9). Their omission does not permit cross-scale pooling: inputs from laboratory, pilot, modeled industrial, and operating industrial configurations may be transferred or combined only when the load-bearing feedstock, unit-operation, product, utility, residual-fate, temporal, and system-boundary bases are aligned or explicitly reconciled. Calculation of an equation-defined quantity does not by itself raise a claim ceiling; the required inputs and linkages must also satisfy the availability, compatibility, and stopping rules defined in Section 2. Each calculation record should identify the route configuration, batch or feedstock group, scale, period, input sources, and conversions used to establish compatible units and bases. Recovery percentages should be expressed as fractions when used as multiplicative factors, and masses and mass flow rates must be reconciled to a common temporal basis.

Route accounting begins with the mass generated or produced within the defined temporal and system boundaries, rather than only with the mass introduced into a laboratory experiment (Figure 1). Accepted feed is calculated using Equation (1):

(1) $$M_{acc}=M_{gen}\times f_{available}\times f_{in}$$

where $M_{gen}$ is the mass generated within the defined temporal and system boundaries; $f_{available}$ is the fraction accessible to the route after accounting for collection, seasonality, storage, and existing uses or prior commitments that constrain diversion; and $f_{in}$ is the fraction of accessible feed that satisfies the predeclared incoming-feed specification. $M_{acc}$, therefore, denotes accepted feed. The denominator of $f_{in}$ is the accessible feed mass, not the total generated mass.

$M_{gen}$ and $M_{acc}$ must be expressed on the same wet- or dry-mass basis, and $0\leq f_{available},f_{in}\leq 1$. Availability denotes access to the route; it neither confers a zero burden nor eliminates opportunity cost. If diversion foregoes a beneficial use, the foregone function must be represented in the environmental and, where relevant, economic counterfactuals. Any constraint represented in $f_{available}$ must not be applied again through $f_{in}$. Where feedstock groups differ, accepted mass should be calculated for each group and then summed, unless aggregate fractions are weighted to reproduce the same groupwise mass balance.

Gross extraction yield is the mass of recovered extract divided by the corresponding feedstock mass, with the process stage and wet- or dry-mass basis stated. Target-specific recovery instead relates the mass of a defined constituent recovered to the mass of that same constituent in the reference input. A stage recovery uses the target mass entering that operation, whereas cumulative recovery uses the target mass in the initial reference feed. These ratios are not interchangeable, and the denominator of a source-reported “yield” is retained as uncertain when it is not specified.

Concentration relates an analyte amount to a stated sample volume or mass, whereas content and purity describe composition on a specified measurement basis. Changes in concentration, purity, or gross extract mass do not alone establish changes in target recovery. Calculation of recovered target mass requires compatible composition and sample mass or volume measurements for the same stream and stage. Total phenolic content reported in assay-standard equivalents is not automatically the measured mass of a specific constituent. For a route defined by a discrete target compound or constituent, Equations (2) and (3) quantify the initial target mass present in the accepted feed and the final target mass retained after sequential recovery:

(2) $$T_{0,t}=M_{acc}\times c_{0,t}$$

(3) $$T_{final,t}=T_{0,t}\prod_{j=1}^{J_{t}}\eta_{j,t}$$

where $c_{0,t}$ is the mass fraction of target t in accepted feed, expressed as kilograms of target per kilogram of accepted feed on the same wet- or dry-mass basis as $M_{\mathrm{acc}}$; $0\leq c_{0,t}\leq 1$. Other units must be converted before use. The composition estimate must represent the accepted material on the stated sampling and moisture basis. Propagation of uncertainty in initial target mass must retain uncertainty in both $M_{\mathrm{acc}}$ and $c_{0,t}$ and any dependence between them.

The conditional recovery $\eta_{j,t}$ is the fraction of target t entering operation j that is retained after that operation; $0\leq \eta_{j,t}\leq 1$, and $J_{t}$ is the number of nested recovery operations. Recovery fractions may be multiplied only when their denominators are conditionally nested and target identity is preserved. A recovery normalized to the initial target amount is already cumulative and must not be multiplied by preceding stage recoveries. Conditional nesting defines a mass-balance relationship, not statistical independence. Recoveries from different batches or operating conditions must not be combined as one observed sequence. Equation (3) applies to a single nested sequence; parallel target-bearing streams, recycle, and recombination require explicit stream-wise mass balances, with recovered target masses summed only across nonoverlapping streams.

$T_{\mathrm{final},t}$ is the retained mass of target t, whereas $Q_{\mathrm{final},p}$ is the total mass of product stream p at its stated stage before conformity assessment. These quantities have distinct accounting bases.

Uncertainty in cumulative recovery is distinct from its point calculation. When the same intermediate target-mass measurement is shared by the numerator of one stage ratio and the denominator of the next, the ratio errors can be correlated. In a product of ratios constructed from the same sequential mass records, intermediate masses cancel algebraically. Uncertainty propagation should therefore preserve the shared measurements or their covariance rather than count stage errors as independent contributions. The product of stage mean recoveries should not be assumed to equal the mean of batch-level cumulative recovery.

The product stage must be specified when reporting isolated or formulated mass. Isolated-product mass may include co-extracted constituents or impurities and formulated-product mass may also include carriers or other added ingredients; neither is interchangeable with recovered target mass. The product mass used for specification assessment must refer to the same defined stream and stage as the acceptance criteria. For an FC route that is not defined by a discrete target constituent, accounting may proceed from accepted feed to the defined isolated or formulated product and its specification assessment. This alternative does not preclude target-specific recovery analysis for an FC product when the target and its input basis are adequately defined.

Specification-conforming output is calculated using Equation (4):

(4) $$Q_{spec,p}=Q_{final,p}\times f_{grade,p}$$

where $f_{\mathrm{grade},p}$ is the mass fraction of product stream p, at its stated stage, that meets all applicable predeclared acceptance criteria; $0\leq f_{\mathrm{grade},p}\leq 1$. For a homogeneous batch assessed as one unit, $f_{\mathrm{grade},p}$ is 1 when all applicable criteria are demonstrably met, 0 when at least one is demonstrably not met, and unassigned when conformity cannot be determined. Near an acceptance limit, the record should identify the test method, sampling basis, available measurement-uncertainty information, and decision rule.

An observed fractional value requires physically separated and quantified conforming portions or aggregation of batches with established conformity decisions over a defined production period. For aggregation, conforming batch mass is divided by total output mass for the same assessed set, which must also define $Q_{\mathrm{final},p}$. Batches with unresolved conformity must be identified separately, not classified as failures or omitted from a total-production claim. The fraction is neither the proportion of specification items passed nor the unweighted proportion of passing batches; a P code is not a numerical value of $f_{\mathrm{grade},p}$.

A model-assumed or estimated expected fraction must identify its evidential basis and uncertainty and must not be presented as an observed conformity rate. Future estimates should account for any dependence between batch mass and conformity before multiplying their separate means. $Q_{\mathrm{spec},p}$ cannot be established as an observed output without final product mass, an applicable predeclared acceptance rule, and sufficient conformity evidence. Establishing $Q_{\mathrm{spec},p}$ does not establish benchmark-equivalent service or market uptake.

For any product comparison in which equivalent service depends on relative use level or functional performance, benchmark-equivalent service may be calculated using Equation (5) only after specification-conforming output and comparable performance data have been established:

(5) $$S_{eq,p,s}=Q_{spec,p}\times \frac{u_{ref,p,s}}{u_{test,p,s}}$$

where $u_{\mathrm{ref},p,s}$ and $u_{\mathrm{test},p,s}$ are the amounts of reference and recovered products, respectively, needed to provide the same service s at a stated performance criterion under matched test and use conditions. Their ratio gives the reference-product amount represented by a unit amount of recovered product. Both amounts must be strictly positive and use compatible bases; the recovered-product amount must also match the product-mass basis in Equation (4). The ratio applies only to the documented comparator, grade, formulation, application, and use range, with evidence supporting repeated provision of that service.

Proportional scaling does not establish linearity across the full concentration–response range. Saturation, thresholds, formulation interactions, or stability may make the required use level condition-specific. An equal-mass assumption, activity ratio, or nonsignificant difference in one property does not establish the use-level ratio. When nonlinear responses or multiple required functions preclude a single factor, the conditional comparison should be retained without assigning an unsupported coefficient. $S_{\mathrm{eq},p,s}$ is expressed in reference-product-equivalent units for service s; it does not represent recovered-product mass or demonstrate market uptake. This conversion does not supply the functional-unit, boundary, inventory, or counterfactual evidence required for comparative LCA.

Market uptake remains distinct from both specification-conforming manufacturing output and benchmark-equivalent service. Within the broader claim class of modeled substitution potential, Equation (6) provides this review’s proposed definition of scenario-constrained substitution potential. Numerical use would require established benchmark-equivalent service and a defined demand context and realization scenario:

(6) $$S_{sub,scenario,p,s}=min\left(S_{eq,p,s},D_{eq,p,s}\right)\times f_{real,p,s}$$

where $D_{\mathrm{eq},p,s}$ is addressable demand in the same units as $S_{\mathrm{eq},p,s}$, and $f_{\mathrm{real},p,s}$ is the scenario-specific fraction of supply- and demand-capped potential expected to reduce incumbent consumption; $0\leq f_{\mathrm{real},p,s}\leq 1$. Applied after the minimum operator, this coefficient represents only post-cap realization constraints. It must not duplicate the functionality adjustment in Equation (5) or constraints already included in feed availability, supply, or demand. Its source and empirical or assumed basis must be stated for the relevant market and period. Without empirical support, the value remains a scenario assumption; missing realization evidence does not justify assigning either 0 or 1 by default.

Supply and addressable demand must share a service, comparator grade, geographic or market boundary, and temporal basis. Total market size is not necessarily addressable demand. The record should identify the binding cap, service-specific ratios, and allocations across products, services, or alternative routes without counting the same feed or product mass more than once.

$S_{\mathrm{sub},\mathrm{scenario},p,s}$ supports a scenario-conditional modeled-substitution-potential claim. Neither modeled substitution nor an LCA substitution or avoided-burden credit constitutes observed displacement. This review defines observed displacement as empirical evidence linking recovered-product uptake to reduced procurement, production, or consumption of a named incumbent within a defined market or supply relationship. This criterion and the post-cap realization form in Equation (6) are operational choices of this review, informed by but distinct from the broader substitution-accounting principles in [20].

Future sensitivity analyses should compare supported or explicitly assumed realization values and identify changes in the binding cap when supply or demand varies. With fixed supply and demand, Equation (6) varies linearly with $f_{\mathrm{real},p,s}$; joint variation requires mutually consistent scenarios. An output calculated from mean inputs need not equal the mean of scenario-specific outputs. The formal range of 0–1 defines theoretical coefficient bounds, not an empirical uncertainty interval. Sensitivity analysis cannot replace missing service-equivalence or demand evidence.

### 3.3. Physical Resource Productivity and Environmental Counterfactuals

Manufacturing- and service-level physical resource productivity can be quantified only when the output and physical input refer to the same route, product stream, accounting basis, and system boundary. For a named physical resource input *i*, Equation (7) defines specification-conforming product mass per unit of attributable input, whereas Equation (8) defines benchmark-equivalent service per unit of attributable input:

(7) $$RP_{mfg,i,p}=\frac{Q_{spec,p}}{R_{i,p}^{attrib}}$$

(8) $$RP_{svc,i,p,s}=\frac{S_{eq,p,s}}{R_{i,p}^{attrib}}$$

where $R_{i,p}^{attrib}$ denotes physical input i assigned to product stream p over the same route and system boundary as the numerator; for a single-output route, $R_{i,p}^{attrib}=R_{i}$. A product-specific denominator is admissible only when input assignment follows a reported and justified subdivision or allocation rule. Under system expansion, the complete product basket and expanded functions must be retained rather than converted into unsupported product-specific ratios. If shared inputs cannot be defensibly assigned, the complete product basket and shared input profile should be reported, and ratios sharing an unallocated denominator should not be summed. A net input may be used only when the source explicitly defines its components and the corresponding netting procedure. The numerator and denominator must also refer to the same temporal basis, and the attributable input must be greater than zero. A zero or negative net input should be reported as a balance rather than converted to a productivity ratio. For future numerical applications, uncertainty assessment should preserve numerator–denominator dependence arising from shared measurements, batches, or balances and examine sensitivity to small denominators or uncertain net inputs. Comparisons of allocation assumptions should retain the same output definition and accounting scope.

Biomass input, freshwater input or explicitly defined net water use, fresh-solvent makeup, process energy, and attributable land are reported as separate input-specific profiles rather than a composite score. A higher *RP* value indicates more specification-conforming output or benchmark-equivalent service per unit of the named input within the stated boundary; it does not support comparisons across unlike resource categories. Gross solvent circulation is not fresh-solvent makeup, and land attribution to a side stream requires an explicit, defensible upstream rule. Cost, labor, and capital belong to techno-economic assessment, not these physical ratios. Resource productivity alone cannot establish environmental improvement because it does not represent impact pathways or an environmental counterfactual. For impact category *h*, Equation (9) defines the modeled difference between the proposed and counterfactual systems:

(9) $$\Delta I_{h}=I_{proposed,h}-I_{counterfactual,h}$$

where $I_{proposed,h}$ and $I_{counterfactual,h}$ are the category-specific modeled impacts of the complete proposed and counterfactual systems. The systems must provide the same quantified function using a common functional unit and corresponding reference flows, with aligned or explicitly reconciled boundaries, geographic and temporal contexts, and a compatible life cycle impact assessment (LCIA) method and version.

To preserve functional equivalence, the proposed system includes the valorization route and replacement of any beneficial function foregone through feedstock diversion. The counterfactual includes the baseline feedstock fate or use and the incumbent system providing the matched service. Alternative system-expansion or avoided-burden conventions are admissible only when functions, boundaries, and sign conventions are internally consistent and credits are not counted twice.

A negative Δ*I_h_* indicates a lower modeled point estimate for the proposed system only for the stated impact category, functional unit, geographic and temporal context, boundary, allocation or system-expansion rule, and assumptions. It does not establish statistical significance or overall environmental superiority. Uncertainty assessment should preserve dependencies from shared background data and assumptions. Sign stability supports robustness only within the reported sensitivity ranges or scenarios; source-defined probabilistic intervals and probabilities require separate interpretation and do not establish sign invariance over all realizations.

ISO 14040 and ISO 14044 provide life-cycle assessment principles, framework, requirements, and guidance, whereas ISO 59020 addresses circularity measurement and assessment [45,46,48]. Circularity is therefore a distinct analytical dimension, not a substitute for category-specific environmental assessment. Under the rules in Section 2, missing or unverified essential evidence or a confirmed incompatible basis limits the environmental claim to its last supported conclusion, potentially a category-specific source-reported result. Partial evidence supports a bounded conclusion only when it directly supports that conclusion; missing claim-relevant uncertainty evidence precludes a robustness claim.

### 3.4. Claim-Specific Ceilings and Stopping Rules

Specification-adjusted route accounting assigns a separate ceiling to technical realization, physical resource productivity, economic plausibility, environmental improvement, modeled substitution potential, and observed displacement (Table 1; Figure 2). A defined route and traceable technical basis are shared prerequisites; modeled TEA or LCA remains admissible as model-conditional evidence when realization is not claimed. Product or service equivalence is required only for comparisons that depend on matched function or use. Physical, economic, and environmental paths then proceed separately, with TEA and LCA remaining parallel. The modeled-substitution implementation proposed here requires comparator, service-equivalence, supply, demand, and realization assumptions. Observed displacement is independent, requires empirical named-incumbent reduction within a defined relationship, and does not require a prior substitution model.

Under the proposed rules, assessment begins by defining the intended claim, route configuration, product state, and comparison basis. Requirements are then identified as essential, conditionally essential, or outside the scope of that claim. The relevant evidence and linkages are assessed before identifying the first unresolved requirement that prevents the intended conclusion and any narrower conclusion that remains supported. This ordering follows the dependencies of the claim rather than the numerical sequence of F01–F10. When several requirements remain unresolved, the decision record should identify the initial stopping condition and the other outstanding requirements.

Explicit evidence and aligned or explicitly reconciled linkages are necessary for a conclusion with complete reporting and compatible evidence, but they do not guarantee a favorable result or fulfillment of an acceptance criterion. P supports a bounded or conditional conclusion only when that conclusion is directly supported by the accessible evidence; P arising from an unverified essential element leaves the dependent claim unestablished. NR, a confirmed incompatible linkage, or documented failure of a required criterion limits the affected claim. These restrictions do not remove a separately supported result on another path, and claim ceilings are not averaged. Technical realization may therefore be supported without a physical resource-productivity calculation, economic plausibility without environmental improvement, and a modeled environmental result without observed displacement.

## 4. Cross-Stream Synthesis and Route-Level Applications

### 4.1. Cross-Stream Synthesis: Recurring Dependencies and Contested Boundaries

The literature synthesis identifies three cross-stream findings that inform the proposed framework. First, recoverable output depends on the full route, including feedstock identity, availability and acceptance, stage selectivity, downstream separation, formulation, and storage [2,6,49]. A favorable extraction yield or marker response can remain a valid process result without establishing final-product conformity. The final stream, product basis, predeclared acceptance rule, and conformity evidence must refer to the same configuration. At the feedstock end, existing uses, burden attribution, and baseline fate also matter: an accessible side stream is not necessarily burden-free or economically uncommitted. Distinguishing generated, available, and accepted feed, and retaining each downstream output state, makes the accounting basis explicit when moisture adjustment, purification, carrier addition, or rejection changes the reported mass. The corresponding inputs and baseline must remain matched to that output state.

Second, detailed analysis within one evidence stream does not resolve gaps between streams. TEA and LCA may use a scale, product grade, output basis, utilities, residual fate, functional unit, or counterfactual that differs from the experimental route. Their results remain conditional on those assumptions and cannot supply missing conformity, service-equivalence, or market evidence. Conversely, a supported recovery or functional response does not establish economic or environmental performance. Circularity, retained quality, and value measures also address distinct questions and may coexist with category-specific environmental trade-offs [24,38,50,51,52]. Linking such results therefore requires alignment or a documented reconciliation of the relevant route and comparison bases, including the original bases, transformation or bridging assumption, and affected claim. Otherwise, apparently consistent findings may concern different denominators or boundaries.

Third, the evidence required for a broader conclusion depends on the intended claim. The proposed framework translates the synthesis into route-identification fields, distinct output states, and claim-specific requirements and stopping rules (Section 2 and Section 3). Physical resource productivity, economic plausibility, and environmental improvement retain separate evidence requirements after their shared route prerequisites. Benchmark-equivalent service is required when a comparison depends on matched function or use and is a prerequisite for Equation (6); observed displacement remains a separate empirical claim. Partial evidence supports a narrower conclusion only when it directly supports that conclusion and no stopping condition applies. An unresolved essential requirement limits the dependent claim without invalidating a separately supported result or implying that an inapplicable claim failed. These distinctions identify the additional evidence needed for a broader conclusion without converting evidence gaps into a composite sustainability or quality score.

Table 2 relates the three findings to the seven evidence streams and distinguishes literature-based principles, claim-scope boundaries, and the operational treatment proposed here. Section 4.2, Section 4.3, Section 4.4, Section 4.5, Section 4.6 and Section 4.7 illustrate selected elements retrospectively using the author-curated cases; Supplementary Workbook S1 provides the associated package, configuration, evidence, and claim-path records.

### 4.2. Analytical Case Set and Unit of Inference

The retrospective illustration comprised 17 author-curated evidence packages: four registered as MI, seven as CC, and six as FC (Section 3.1). A separate classification assigns 11 core and six boundary-condition roles to the same packages; these counts are not additive. Archetype labels do not exclude additional product requirements, whose proposed treatment is described in Section 3.1. The packages contain 45 registered analytical records (Section 2.2). Their nested organization describes evidence coverage, not independent replication or greater evidentiary weight.

The selected set included agri-food byproduct routes and other plant-derived systems that differed in product archetype, feedstock, process completeness, scale, application context, and TEA and LCA coverage. Core packages supported the main exposition, whereas boundary-condition packages illustrated the application of the same rules to contrasting evidence structures. These roles do not denote evidence-quality grades, representativeness, or validation status. Beyond the four worked cases, core packages included a *Peucedanum* reference-substance route; polyphenol and standardized-extract routes involving red-wine pomace, Ginkgo, and industrial extracts; and citrus-pectin processing and application routes [53,54,55,56,57,58,59].

The nested records distinguish source-defined differences in technology, solvent-to-feed ratio, scale, botanical source, pulsed-electric-field condition, solvent source, and pretreatment, as well as stage-specific information and a within-source comparison. These differences do not all represent alternative routes. In particular, MI-03 includes a downstream purification record, whereas CC-04 contains records for different botanical extracts. Differences in scale were not interpreted as a longitudinal scale-up series. Configuration and scenario records remained distinct, and linkage of their results required alignment or explicit reconciliation of the relevant route, product, and assessment bases. When accessible evidence did not distinguish configuration-specific conditions and outcomes, inference remained at the package level. Supplementary Figure S1 summarizes the case-set architecture, and Supplementary Workbook S1 provides the associated identifiers, organizational links, source locators, and limits of inference.

### 4.3. From Endpoint Characterization to Specification-Conforming Output

All 17 packages reported a terminal chemical or functional endpoint, but evidence for specification-conforming output was uneven. Characterization did not necessarily include a predeclared specification, demonstrated conformity, repeat-batch conformity, or documented commercial release. The *Peucedanum* and artemisinin packages provided detailed identity and purification evidence, while the pilot-scale citrus-pectin package included reported food-grade acceptance criteria [55,59,60,61]. Several standardized extracts lacked complete predeclared compositional ranges or repeat-batch acceptance rules in the assessed records. These distinctions limit the claim supported by the accessible evidence; they do not establish that the products failed unverified criteria. A reported measurement supports characterization, and any additional conformity conclusion is limited to the documented criterion, assessed batch or stream, and evidence that the acceptance rule preceded outcome evaluation.

These findings were interpreted using the claim-specific requirements in Section 2.5 and Table 1. The first unsupported applicable requirement limited the affected conclusion, while compatible technical, TEA, and LCA findings retained their separate scopes. Endpoint characterization alone did not establish release, repeatability, benchmark-equivalent service, or substitution. Cell-level decisions are recorded in Supplementary Workbook S1.

### 4.4. Worked Demonstrations of Route-Level Decision Changes

Four packages were used for detailed worked demonstrations within the broader 17-package illustration (Figure 3; Supplementary Table S3). MI-02 examines sequential target losses; FC-04 contrasts extraction yield with process conditions and product properties; CC-05 illustrates price-dependent economic preference; and CC-02 distinguishes deterministic and probabilistic economic findings from separately scoped environmental evidence. These cases address complementary interpretive problems and do not constitute a representative sample or a basis for ranking the packages. Three concern agri-food byproduct routes. The artemisinin package, MI-02, provides a molecular-identity contrast because its reported sequential recoveries distinguish stage-specific retention from complete-route target recovery.

The artemisinin package (MI-02) contains one registered route configuration, MI-02-C01, comprising maceration, flash chromatography, and crystallization. The source-reported conditional recoveries of 90.0%, 87.08%, and 47.56% refer to the artemisinin entering each stage, not to total plant mass or a common initial denominator [60]. Multiplying the conditionally nested recoveries gives cumulative retention of 90.0%, 78.4%, and 37.3% on an initial artemisinin basis of 100%; this review did not calculate absolute target mass. The 78.4% intermediate is author-derived. The final 37.3% also rounds the source-reported overall recovery of 37.27%. Agreement at this displayed precision is an arithmetic consistency check by the author, not independent validation. Treating maceration recovery as complete-route retention would give an estimate 2.41 times the final retention; this illustrative comparison does not attribute an error to the source authors.

The point reconstruction has no propagated uncertainty interval. It describes retained target mass, not product purity or specification-conforming output, and release evidence remains partial. Physical resource productivity, economic plausibility, environmental improvement, and modeled substitution were outside the MI-02 applicability map; observed displacement remains unestablished because the accessible material and retained F10 review documentation were insufficient for a nonreporting judgment (P). The case illustrates how downstream losses separate initial recovery from complete-route target retention.

The grapefruit-pectin package (FC-04) contains a conventional heating extraction reference (CHE; FC-04-C01), an ultrasound-assisted heating extraction variant (UAHE; FC-04-C02), and their within-source comparison record (FC-04-C03); the comparison is not a third extraction route. For UAHE, the source reported a 27.34% yield on a mass basis that was not explicit in the assessed record, a 16.34% relative increase over CHE, a temperature 13.3 °C lower, and an extraction time 37.78% shorter [62]. The yield increase is relative to CHE, rather than a percentage-point difference. These results compare the source-defined configurations and do not isolate the effect of ultrasound with all other conditions held constant. Intrinsic viscosity and molecular weight were lower. The degree of esterification was slightly lower, but the difference was not statistically significant; reported increases in branching and purity were not uniformly supported by significant contrasts. A nonsignificant difference does not establish equivalence. The value of these property changes depends on the intended application and cannot be inferred from their direction alone. Without documented predeclared release criteria and benchmark-matched gelation or use-level evidence, the supported conclusion remains a configuration-specific relationship among yield, operating conditions, and product properties. It does not establish service equivalence, substitution, displacement, or general route superiority. The case therefore illustrates the need to evaluate process–property trade-offs against a defined application rather than yield alone.

The red-grape-pomace package (CC-05) contains a solid–liquid extraction reference, SLE-01 (CC-05-C01), and four modeled pulsed electric field (PEF) pretreatment scenarios, PEF-02–05 (CC-05-C02–C05) [63]. PEF-02 targets higher yield; PEF-03, PEF-04, and PEF-05 target lower temperature, shorter processing time, and lower ethanol concentration, respectively. The modeled final product is a spray-dried extract formulated with maltodextrin and gum Arabic. The source standardizes its total polyphenol content (TPC) percentage at approximately 46% across scenarios; this model composition does not establish a commercial grade or release decision. The modeled output is 22 kg of formulated product per batch, approximately 168 t year^−1^, with 7920 operating hours per year.

Within that model, PEF-02 was preferred below $249 kg^−1^ and SLE-01 at or above $250 kg^−1^. These reported regions bound a switching interval without identifying an exact switching price [63]. The dollar symbol preserves the source notation; the currency jurisdiction and monetary base year were not verified in the assessed article. At base product prices rounded to approximately $233 kg^−1^, the source reported a 2.72% reduction in unit production cost and an 8.11% increase in return on investment for PEF-02 relative to SLE-01. These are source-reported relative changes, not percentage-point differences. The comparison supports a price-dependent economic preference within the stated product, scale, and configuration assumptions. It does not reproduce the source model or establish robustness to all uncertain inputs, environmental improvement, modeled substitution, or observed displacement.

The microencapsulated pomegranate-peel extract powder (PPEP) package, CC-02, contains three modeled economic scale records: Case 1 at 5 kg day^−1^ (CC-02-C01), Case 2 at 100 kg day^−1^ (CC-02-C02), and Case 3 at 5000 kg day^−1^ (CC-02-C03). Case 1 is the registered parent configuration and Cases 2 and 3 are modeled scenarios; these records do not describe observed stages in a longitudinal scale-up study. Among the modeled cases, Case 3 had the highest reported deterministic net present value (NPV) and the lowest reported levelized cost of production, whereas Case 2 had the highest reported probability of positive NPV, P(NPV > 0) = 91.9%, in the source-reported 10,000-run Monte Carlo analysis [44]. The point estimates and the probability of exceeding zero answer different economic questions under their respective assumptions. The simulation iterations are not experimental replicates or additional analytical records in this review, and the probability is conditional on the modeled inputs and distributions rather than an observed probability of business success.

A separate package-level LCA identified freeze-drying as the dominant hotspot. The assessed record did not establish its attribution to a specific TEA scale, limiting integration of the economic and environmental results at that scale. The absence of a functionally equivalent counterfactual separately limited a claim of comparative environmental improvement. These limitations preserve the narrower hotspot finding and do not establish that the TEA and LCA records are incompatible. The reported model results do not, by themselves, establish conformity of the final formulated powder or justify treating its total mass as recovered constituent mass. Neither branch established benchmark-equivalent service, modeled substitution, or observed displacement. The case therefore separates the choice of economic decision criterion from the evidence needed to combine economic and environmental findings at a specified scale.

### 4.5. Decision Consequences and Claim-Path Audits

Figure 3 and Supplementary Table S3 retain each source metric while examining the route, product state, comparison basis, and intended claim it can support. The resulting interpretations range from complete-route target retention and process–property trade-offs to price-dependent economic preference and parallel model-conditional economic and environmental findings. The broader interpretations considered here are illustrative extensions, not claims attributed to the source authors or outcomes of a head-to-head comparison of assessment methods. The framework preserves the reported results while making their supporting bases and unresolved requirements explicit.

Supplementary Figure S2 contrasts unresolved requirements with claims outside the documented assessment scope. MI-02 supports conditionally nested target recovery, but full release conformity is unestablished. Physical resource productivity, economic plausibility, environmental improvement, and modeled substitution were outside its applicability map; these scope decisions do not indicate route failure. CC-02 supports route definition and distinct deterministic and probabilistic economic results, while full release conformity is also unestablished. Its LCA supports hotspot identification: the missing functionally equivalent counterfactual limits comparative environmental inference, whereas unestablished TEA-scale attribution separately limits integration at that scale. These parallel results do not jointly establish route superiority or modeled substitution. Observed displacement remains unestablished for both packages: MI-02 is coded P because its documented F10 review scope is insufficient for a nonreporting judgment, whereas CC-02 retains NR within its documented review scope. For CC-02 and CC-05, this review did not calculate package-level physical resource-productivity ratios; non-calculation does not establish that the sources omitted the required quantities or that the routes had poor resource productivity.

The rules in Section 2.5 distinguish directly supported narrower conclusions from claims whose essential requirements remain unverified. Availability and provenance codes are categorical records, not numerical weights in Equations (1)–(9). Likewise, a computable point estimate with unquantified uncertainty differs from a quantity that cannot be established because an essential input is unverified. The audit trail links package-level evidence to package–claim decisions and uses configuration-specific findings where separable. It does not provide a complete assessment of every nested record or a systematic A01–A08 evaluation for every package–claim combination; the resulting limits on claim-level reproducibility are described in Section 4.7. The case assessment remains an internal demonstration of the proposed framework.

### 4.6. Boundary-Condition Demonstrations Across Contrasting Evidence Structures

The six boundary-condition packages formed part of the same retrospective assessment as the 11 core packages and were interpreted using the same availability, provenance, compatibility, and stopping rules. They illustrate contrasting recovery bases, process configurations, scales, and assessment structures across the three product archetypes. Their analytical role does not make them an external validation set or support estimates of coding accuracy, reliability, generalizability, or route success. Package-level evidence and claim limitations are summarized in Supplementary Table S4.

The molecular-identity cases illustrate why recovery must retain its target, numerator, denominator, processing stage, and scale. The steviol-glycoside records distinguish crystallization from recovery from the residual stream and from reported overall or scale-up results [64]. The curcuminoid records distinguish target-specific chromatographic recovery from overall recovery for the combined procedure [65]. These quantities cannot be combined or ranked as interchangeable measures of route output. Purity describes the composition of the recovered product and is distinct from recovery of the target mass. Interpretation therefore remains tied to the documented stage and product state; a high conditional recovery does not establish complete-route retention or routine product release.

The composition-constrained cases distinguish model outputs from product conformity and service equivalence. The pine-residue LCA provides comparisons within its source-defined configurations and assessment basis [66]. Interpreting those comparisons as environmental improvement for interchangeable products additionally requires evidence of functional equivalence. Without that evidence, the modeled results retain their stated scope but do not establish a service-equivalent advantage. For spruce-bark tannin, TEA and LCA provide conditional economic and category-specific environmental results in parallel [67]. An absolute impact estimate or a hotspot result does not establish improvement relative to an unspecified counterfactual. Neither case, by itself, establishes routine product release, benchmark-equivalent service, the modeled-substitution implementation proposed here, or observed displacement.

The function-constrained cases illustrate different limits on interpretation. The prospective rhamnogalacturonan-I pectin package supports conditional economic findings and category-specific environmental results within the reported scenarios [68]. Its heat-recovery credit assumes displacement of natural-gas heat, and its economic discussion compares pectin production with commercial prebiotic prices. These provide assessment context, not a model of recovered-pectin substitution. For Kinnow-peel pectin, commercial-product comparisons support conclusions about measured properties under the reported test conditions, while the LCA supports hotspot identification [69]. No assessment against predeclared release criteria was identified in the reviewed record; the property comparisons also did not establish equivalence at a matched service and use level. Supplementary Table S4 summarizes these package-specific limits. Across the three archetypes, the cases illustrate selected applications of the proposed rules, not their accuracy or the frequency of successful industrial translation.

### 4.7. Evidence Availability, Provenance, Compatibility, and Interpretive Limits

Figure 4 summarizes 170 package–field availability and completeness decisions (17 evidence packages × 10 fields). Each field contributes one summary per package, regardless of the number of nested analytical records. The applicable count is 17 minus the number coded NA; E, P, and NR partition that count. These denominators describe coverage within the selected set, not independent observations or product pass rates.

Feedstock identity and route configuration were explicit in all 17 packages, whereas stage recovery was explicit in three and partial or bounded in 14. Acceptance specifications were coded E in one package, P in ten, and NR in six; specification-conformity evidence was coded E in one, P in nine, and NR in seven. Benchmark-equivalent service had no E codes, with three P, ten NR, and four NA. TEA had five E, five NR, and seven NA, whereas LCA had 11 E, one NR, and five NA. Modeled substitution potential had no E or P codes, with 13 NR and four NA. These distributions distinguish documented route information from the evidence needed for product, service, and substitution claims; they do not rank route performance or research quality.

Observed displacement was examined across all 17 packages as a cross-cutting question, not as a reporting obligation imposed on every source study. No selected package provided verifiable observed-displacement evidence at the documented access level. Twelve packages were coded NR within their documented review scope. Five packages—MI-01, MI-02, MI-03, CC-03, and FC-04—were coded P because the accessible material and retained field-specific review documentation were insufficient to support a complete F10 nonreporting judgment. Earlier full-text access recorded for the latter four packages did not, by itself, establish sufficient F10 review coverage. These access-limited P codes do not indicate partial empirical displacement. Access-limited P judgments and P judgments supported by incomplete evidence require their distinct rationales to be retained. Neither P nor NR establishes that displacement did not occur.

The access-stratified summary includes 16 packages with recorded full-text access, including CC-02’s publisher-hosted unedited source, and one limited-access package, MI-01 (Table S6). Within the recorded full-text stratum, acceptance specifications were E = 1, P = 9, and NR = 6; specification-conformity evidence was E = 1, P = 8, and NR = 7. Observed displacement was NR in 12 packages and P in four—MI-02, MI-03, CC-03, and FC-04—because sufficient F10 review coverage could not be established from the accessible material and retained records. MI-01 remained P in the limited-access stratum, giving 12 NR and five P overall. These P codes do not indicate partial empirical displacement. Recorded full-text access and sufficient review of a specific field are distinct. With only one limited-access package, this descriptive breakdown cannot estimate an effect of access or establish robustness.

The worked cases illustrate why these summaries require claim-specific interpretation (Section 4.4 and Section 4.5). MI-02 combines explicit recovery evidence with an SR/AD cumulative-retention calculation; neither establishes conformity or release, and its physical resource-productivity path was outside the applicability map. CC-02 retains distinct deterministic and probabilistic economic outputs and an LCA hotspot finding. Their coexistence does not establish joint economic and environmental superiority.

The ten-field matrix is distinct from the 102 package–claim records in the Claim Path Stop Audit (17 packages × six claim paths). Those records include scope decisions and qualitative rationales; their number is not a count of supported claims. Matched inputs, multifunctionality, uncertainty, and counterfactual considerations were addressed in the rationales, but auxiliary requirements A01–A08 were not systematically coded for every package–claim combination. The records therefore support structured qualitative interpretation rather than a complete risk-of-bias, certainty, or independently reproducible claim-decision assessment. Agreement on field codes alone would not establish reproducibility of the claim-specific stopping decisions.

Supplementary Figure S3 and Supplementary Workbook S1 separately summarize provenance and linkage compatibility. Provenance describes the claim-relevant output, not every associated input or contextual statement. Compatibility concerns an invoked linkage within its documented boundary. NL identifies eligible evidence for which no linkage is invoked; a blank entry indicates no eligible evidence record for compatibility classification. Neither is evidence that all possible linkages are compatible. An invoked but unevaluable linkage remains unresolved, as specified in Section 2.3. Context-only and gap-rationale records do not supply positive evidence. These summaries describe the selected records at their documented access levels and do not establish coding accuracy, external validity, or field-wide reporting frequencies.

### 4.8. Incremental Contribution Relative to Adjacent Assessment Methods

Integration is already addressed by related assessment approaches. How et al. [39] discuss the integration of fractionation, functionality, and environmental–economic trade-offs for plant-based ingredients, while ISO/TS 14076 addresses combined environmental and economic assessment of process systems [40]. Early-stage TEA and LCA also address shared assessment choices and scale-dependent assumptions [18,19]. Functionality-driven product design links processing choices to intended performance [15], and recycling-quality assessment can combine substitution potential, long-term material use, and environmental impacts [38]. Vadenbo et al. [20] organize substitution around physical resource potential, recovery efficiency, relative functionality, and expected changes in the consumption of competing products. ISO 59020 also recognizes complementary assessment of environmental, social, and economic effects [48]. These approaches differ in purpose and analytical scope. Their use of common bases, functional comparisons, and uncertainty analysis is not a new contribution of this review. The question examined here is how results from such approaches can be linked to a specified route-level claim while retaining their original scope and assumptions. Table 3 compares these approaches by analytical unit, scope, cross-scale linkage, and the role of specification conformity, and identifies the evidence organization proposed here without implying that integration or functional comparison is new.

The proposed framework organizes these inherited principles through a common route key, distinct output states, and claim-specific evidence requirements and stopping rules. Under this procedure, the route key identifies the configuration to which a record refers; the output state distinguishes retained target mass, isolated or formulated product mass, specification-conforming output, and benchmark-equivalent service. Supporting records are examined for the particular claim, and any necessary linkage requires aligned bases or a documented reconciliation. An unresolved prerequisite, an incompatible required linkage, or failure of a required criterion limits the affected conclusion to its last supported scope. This record structure does not replace the calculations or quality criteria of the underlying methods. Economic and environmental results may share inventories or be integrated in a model while retaining separate evidential requirements and conclusions. Likewise, this review’s observed-displacement claim requires an empirical link to reduced incumbent activity; it is not inferred from a modeled substitution result or attributed as a universal requirement to existing substitution frameworks. Table 2 relates the synthesis to the proposed treatment of evidence, and Table 1 presents the claim-specific rules proposed in this review.

The selected cases illustrate this interpretive role: MI-02 retains conditional recoveries and the author-derived cumulative retention; CC-05 retains price-dependent economic preference; and CC-02 retains distinct deterministic and probabilistic economic results alongside a separately scoped LCA hotspot. The framework identifies the route, output state, and claim supported by each result and the additional evidence required for broader conclusions. These interpretations neither attribute unsupported broader claims to the source authors nor demonstrate errors in their methods.

Supplementary Workbook S1 allows inspection of route keys, supporting records, linkage decisions, transformations, and claim rationales. The incomplete implementation of A01–A08 limits full reproduction of claim-specific decisions, as discussed in Section 4.7. The cases provide no head-to-head benchmark or evidence of framework uniqueness, superiority, reliability, or decision benefits. Establishing such benefits requires independent assessment of coding and claim interpretation, independently selected cases, and comparative evaluation using common data and explicit criteria.

## 5. Discussion

### 5.1. Cross-Stream Synthesis and Route-Level Evidence Assessment

The central implication is that a useful measurement or model result may support a narrower conclusion than the route-level claim under consideration. Linking extraction, characterization, TEA, LCA, and market evidence therefore requires attention to the product, scale, and comparison each record supports. The cases illustrate the interpretive consequences of unresolved links, without establishing their field-wide prevalence or importance relative to other translation barriers. Detailed case findings remain in Section 4.4 and Section 4.5. The proposed contribution is this explicit organization of evidence (Section 4.8); it does not pool or strengthen source results, and its reproducibility and decision benefits remain untested.

### 5.2. Product Archetypes and Claim-Specific Evidence Requirements

The archetypes make context-dependent product quality explicit. Circular-economy literature recognizes technical properties, functionality, durability, market value, and environmental attributes as distinct dimensions that no single metric fully captures [50,52]. MI, CC, and FC therefore serve as specification anchors, not quality rankings or regulatory classifications: identity, purity and impurities, compositional acceptance rules, and functional criteria may all be relevant to one product.

For study design, the implication is to define the intended product, use, and acceptance criteria before interpreting favorable measurements. Chemical characterization helps define the product grade and an appropriate comparator; conformity must then be assessed against the applicable predeclared criteria. Repeatable production, commercial release, and benchmark-equivalent service require additional evidence (Section 2.4 and Section 3.1).

TEA and LCA may retain conditional findings based on assumed product properties without confirming those properties empirically. Likewise, substitution frameworks distinguish resource potential and recovery from relative functionality and changes in competing-product consumption [20], supporting separate interpretation of modeled substitution and observed displacement (Section 3.4).

### 5.3. Distinguishing Physical Resource Productivity, Circularity Performance, and Environmental Improvement

The choice of output and input determines what a resource-productivity result means. Replacing gross extract mass with specification-conforming product mass changes the question, while greater output per accepted feed can coexist with higher solvent, water, or energy demand. Study design should therefore identify the claimed efficiency and report other resource demands separately. Definitions are given in Section 3.3; this review calculated no package-level physical resource-productivity ratios.

Circularity, economic performance, and environmental improvement also inform different decisions. ISO 59020:2024 addresses circularity, whereas ISO 14040:2006 and ISO 14044:2006 address LCA [45,46,48]. Greater circulation can add drying or solvent-recovery burdens, and financial preference need not coincide with improvement across environmental impact categories. Environmental interpretation consequently depends on the function, comparator, boundary, location, time, and modeling assumptions [26,45,46].

A hotspot can guide process improvement but does not establish comparative benefit. A stable direction applies only within reported sensitivity ranges or scenarios; probabilistic results retain their own interpretation. No new probabilistic analysis was performed. Multi-output comparisons also require consistent treatment of shared burdens and avoided functions, without duplicate credits or summing mutually exclusive scenarios [20,23]. The operational rules remain in Section 2.5, Section 3.3 and Section 3.4.

### 5.4. Implications for Study Design, Cross-Assessment Linkage, and Laboratory-to-Industrial Translation

Scale transitions change both the evidence needed and the assumptions requiring reassessment [18,19]. Table 4 organizes proposed claim-specific records around laboratory observations, pilot observations, modeled industrial configurations, and operating industrial records. These settings distinguish scale from evidence type and do not constitute a universal maturity sequence or longitudinal validation.

The common route record should preserve feedstock origin and condition, generated versus accessible versus accepted feed, mass basis, process sequence, throughput, time, and final product state and grade. Traceable batch-to-hourly or annual conversions and a defined unit of inference make changes between settings interpretable. Detailed reporting items remain in Section 6 and Supplementary Table S2.

A change record should identify what remains comparable and what requires remeasurement or modeling. Feed moisture, drying, solvent recovery, equipment, formulation, utilities, transport, and residual treatment can change outputs and assessment inputs. Reprocessing requires records of final output and additional inputs without duplicate product accounting. Batch measurements, annual TEA output, and LCA reference flows need traceable product, mass, and time conversions. Evidence does not transfer automatically across scales, but scale differences alone do not establish incompatibility; reconciliation documents a linkage within stated limits without supplying missing evidence (Section 2.3).

TEA requires reassessment of throughput, utilization, output quantity and grade-matched price, input requirements, and capital and operating costs. LCA requires corresponding inventory changes and an aligned functional comparison. These assessments may share inventories or be integrated under ISO/TS 14076:2025 [40], while retaining distinct conclusions. The route record identifies shared and differing assumptions without replacing assessment guidance [19,47].

Operating or sales records support displacement only when they link recovered-product uptake to reduced incumbent activity within a defined period and relationship. The separate modeled-substitution requirements remain in Section 3.4; an observed displacement claim does not require a prior substitution model.

The practical next step depends on the unresolved claim: product measurements, operating and balance records, or a defined service and counterfactual. Users should establish whether the information already exists before selecting further work. The first unresolved requirement is not necessarily the highest investment priority; decision importance, uncertainty, cost, safety, and feasibility also matter. These proposed uses require evaluation of their practical benefits.

### 5.5. Limitations, Appropriate Use, and Priorities for Independent Evaluation

The 17 author-curated packages are nonrepresentative; their 45 analytical records are nested, not independent observations. Database searches served as coverage and gap checks rather than as the basis for exhaustive screening (Section 2.1). The set cannot establish field-wide reporting prevalence, the distribution of omitted counterexamples, or scale-up success rates. Differences in products, scales, access, and purposes limit ranking; no longitudinal follow-up was conducted. Use beyond the selected non-energy plant-derived products and publicly verifiable records requires reassessment.

One author performed selection, extraction, classification, and assessment. No independent reassessment or inter-assessor agreement analysis was added during revision; the same-author second pass checked internal consistency only (Section 2.3). Applicability, partial evidence, reconciliation, and stopping decisions remain assessor-dependent. P rationales must distinguish accessible partial evidence from unverified information, and abstract omission alone cannot justify NR. Coverage checks, access-stratified counts, and boundary-condition cases do not remove these limitations or provide external validation.

The 170 package–field decisions and 102 package–claim records did not fully implement the 42-item checklist. A01–A08 were defined and partly considered qualitatively, but separate applicability, evidence-status, and linkage records were incomplete across package–claim combinations. Claim limits are therefore qualitative interpretations, not outputs of a complete requirement-level matrix. Numerical application of Equations (1)–(9) was limited to normalized cumulative recovery using Equation (3), with no package-level physical resource-productivity calculation (Section 2.5). The assessment does not establish study-level risk of bias, certainty of evidence, or underlying effect accuracy.

The MI-02 cumulative-recovery estimate has no uncertainty interval. Quantification requires replicate-level data or reported uncertainty information preserving dependence among sequential recoveries. Measurement uncertainty, between-batch variation, and modeled-scenario differences must remain distinguishable.

The framework and checklist are proposed aids, not a consensus standard, certification instrument, validated guideline, predictive model, or composite score. Their applicability and completeness remain to be evaluated. Independent assessors should assess agreement in item interpretation, applicability, evidence status, reconciliation, and claim ceilings, including reasons for disagreement and sensitivity to defensible alternative decisions. Independently selected cases should test transferability; complete requirement-level records should address the implementation gap. Prospective user studies should separately evaluate clarity, feasibility, time and effort, and decision effects. A formal reporting guideline would also require stakeholder participation.

## 6. Claim-Specific Reporting Implications

Supplementary Table S2 presents a proposed 42-item checklist in eight reporting domains. Table 5 summarizes each domain’s central question, rationale, and applicability. The seven evidence streams organize the synthesis; the eight domains organize reporting, with scale and multi-output requirements drawing on several streams. Related substitution and circular-quality frameworks inform the links among items, archetypes, output states, and claims [20,52]. The domain summary makes the existing organization explicit; it does not document a formal item-development or redundancy-testing procedure, assign weights, or establish usability or external validity. Citations support the underlying principles rather than every proposed restriction.

The procedure is proposed for retrospective assessment and prospective study design; implementation in this review was limited as described in Section 2.2, Section 2.3, Section 2.4 and Section 2.5 and Section 5.5. Users first define the claim, product and intended use, route, scale, period, observed or modeled basis, and unit of inference. Comparative claims require the relevant comparator, service, functional unit, or counterfactual. Configurations remain separate, and requirements of overlapping archetypes remain applicable. Users justify applicability, including conditional requirements and components of compound items, before assigning evidence status; uncertainty does not justify NA. Applicable items form a claim-specific reporting set, not universal obligations for every study. Prospective use calls for claims, specifications, and comparison criteria to be set before interpreting outcomes. New evidence or changes in the claim, configuration, scale, or comparator require documented reassessment and version control.

Each applicable requirement should be linked to a source record identifying its version, access level, exact locator, conditions, units, product state, observed or modeled result, and judgment rationale. Records may support several items, but summaries must preserve claim-relevant differences and conflicting evidence. Apply the separate availability, provenance, and compatibility rules in Section 2.3. Partial evidence supports only its documented scope; access-limited P supplies no positive evidence for an unverified element, and NR requires sufficient access to assess nonreporting. R requires documented bases, transformations, assumptions, and limits. NL denotes eligible evidence with no invoked linkage, whereas a blank compatibility entry denotes no eligible evidence; neither establishes alignment.

The checklist assigns no weights, scores, pass thresholds, or rankings. Compound items require component-level rationales: explicit evidence for one component does not satisfy another, and one nonapplicable component does not make the whole item NA. Explicit reporting is distinct from criterion satisfaction; characterization, specification, batch conformity, and repeated production require different evidence. E for a TEA or LCA field does not establish complete comparison or uncertainty evidence. An unresolved prerequisite, confirmed incompatible linkage, or unmet required criterion limits the affected claim without being offset by other evidence (Section 3.4). The six paths retain separate conclusions, including parallel economic and environmental findings. Observed displacement does not universally require a prior substitution model, TEA, or LCA; conformity or service equivalence is required when the claimed identity, function, or replacement amount depends on it. Additional sources can fill a gap only with the required information on aligned or explicitly reconciled bases; reconciliation alone cannot supply missing evidence.

The cases illustrate how one supported result can leave another requirement unresolved. MI-02 supports technical recovery findings without establishing a predeclared product specification or batch conformity. CC-02 retains model-conditional TEA results and an LCA hotspot but lacks an established functionally equivalent counterfactual for comparative environmental improvement (Section 4.4). These examples illustrate the reporting logic; they add neither item-level coding nor a complete checklist application.

The proposed output should identify the claim and evaluated unit, supported conclusion and conditions, unmet requirements or criterion failures, and next evidence needed. Other unresolved prerequisites should remain visible alongside the first limiting requirement, with gaps distinguished from demonstrated failures. The ten summary fields do not replace item-level records, and one requirement may inform several paths. Section 5.5 describes the retrospective scope and incomplete A01–A08 implementation. A completed reporting record supports only the documented claim; it does not itself establish conformity, comparative benefit, or industrial success.

## 7. Conclusions

Gross extraction yield is informative on its stated basis; broader claims require evidence about the product, process, and comparison. This critical integrative review synthesizes these relationships and proposes specification-adjusted route accounting to link chemical identity, composition, product state, and route-identification fields to claim-specific requirements and stopping rules. Laboratory, pilot, modeled industrial, and operating industrial evidence requires compatible or explicitly reconciled bases. Characterization, conformity, functional equivalence, modeled substitution, and observed displacement remain distinct conclusions.

The selected cases illustrate these distinctions: downstream separation altered artemisinin retention; higher grapefruit-pectin yield did not establish functional equivalence; and red-grape-pomace economic preference depended on price. For microencapsulated pomegranate-peel extract powder, the highest deterministic NPV and highest probability of positive NPV occurred at different modeled scales, while a separately scoped LCA identified a freeze-drying hotspot without establishing comparative improvement. These findings retain value within their stated conditions. No package provided verifiable observed-displacement evidence at the documented access level.

The 17 purposively selected packages constitute a retrospective illustration, not a longitudinal scale-up cohort or basis for field-wide prevalence and success rates. Single-author assessment and limited source access constrain interpretation. Numerical application was limited to normalized cumulative target recovery; the remaining equations were proposed definitions. Neither all configuration–claim requirements nor the full 42-item checklist were implemented. The review did not establish independent reproducibility or formally assess study-level risk of bias or certainty of effects.

The framework offers a structure for recording supported conclusions and identifying further evidence needs. Independent reassessment should examine classifications and stopping decisions; independently selected cases should test transferability. Prospective studies should define routes, product states, claims, and decision criteria before assessment, then evaluate decision clarity, consistency, and workload. An unresolved requirement identifies a need for documentation or measurement, not evidence of poor route performance.

## Figures and Tables

**Figure 1:**
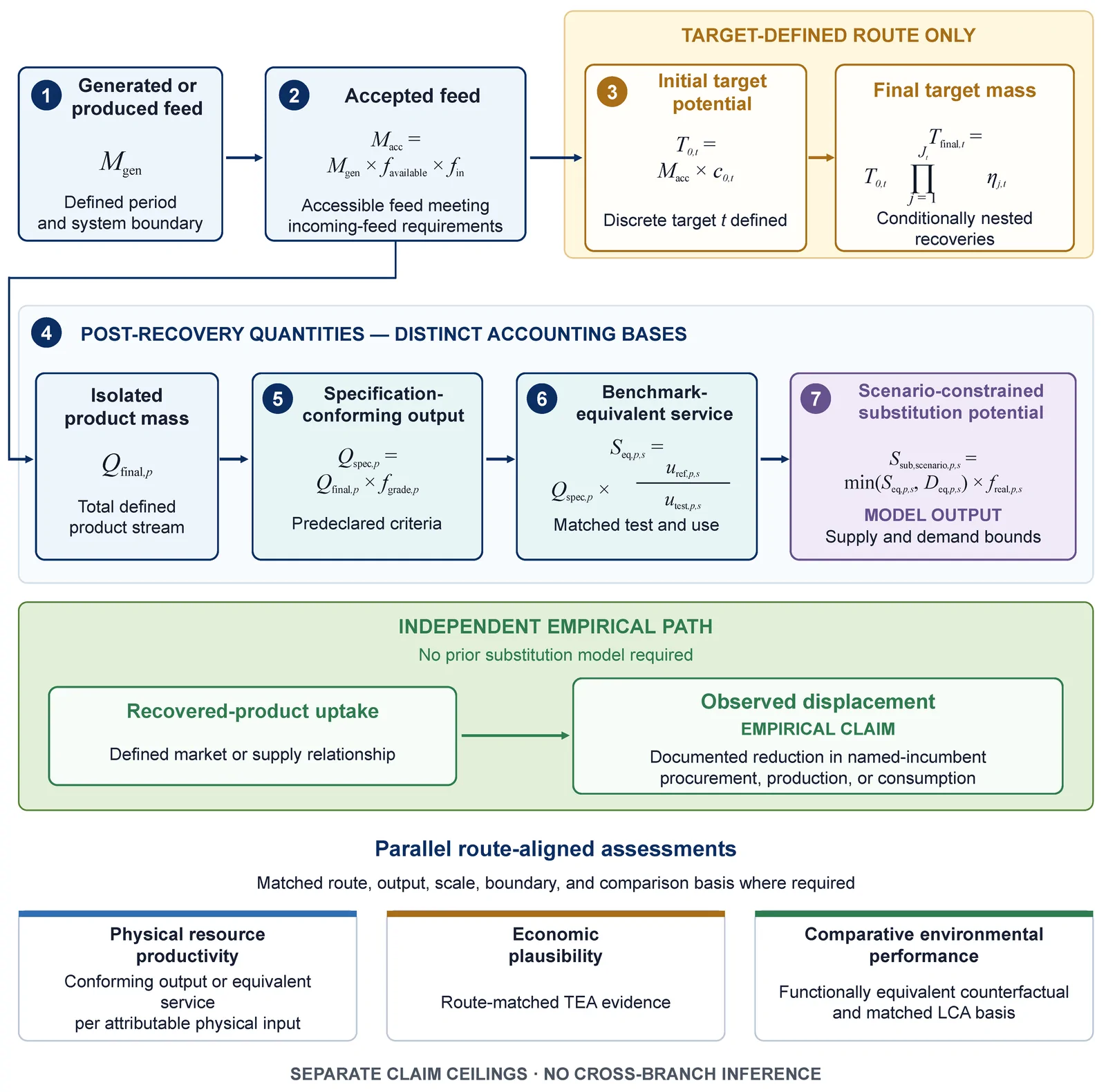
Proposed specification-adjusted route-accounting chain. Generated feed is adjusted for genuine availability and incoming-feed acceptance. For a target-defined route, the initial target mass is based on accepted feed and target composition, and conditionally nested recoveries describe cumulative target retention. Final target mass and total isolated or formulated product mass have distinct accounting bases; this distinction does not require their numerical values to differ. Assessment against applicable predeclared criteria identifies specification-conforming output when conformity is demonstrated. When conformity cannot be determined, the conformity fraction remains unassigned. Benchmark-equivalent service applies only when equivalence is claimed under matched test and use conditions. Scenario-constrained substitution potential combines compatible supply and demand with an explicit post-cap realization assumption. It is a model output, not observed displacement. Observed displacement is a separate empirical claim requiring documented reduction in a named incumbent within a defined market or supply relationship; a prior substitution model is not required. Physical resource productivity, economic plausibility, and comparative environmental performance remain parallel, route-aligned assessments with separate evidence requirements and interpretation limits. The diagram summarizes proposed accounting definitions and claim relationships; it does not imply that all equations were numerically applied in the retrospective cases. Symbols and conditions follow Equations (1)–(6) in Section 3.2. TEA, techno-economic assessment; LCA, life cycle assessment.

**Figure 2:**
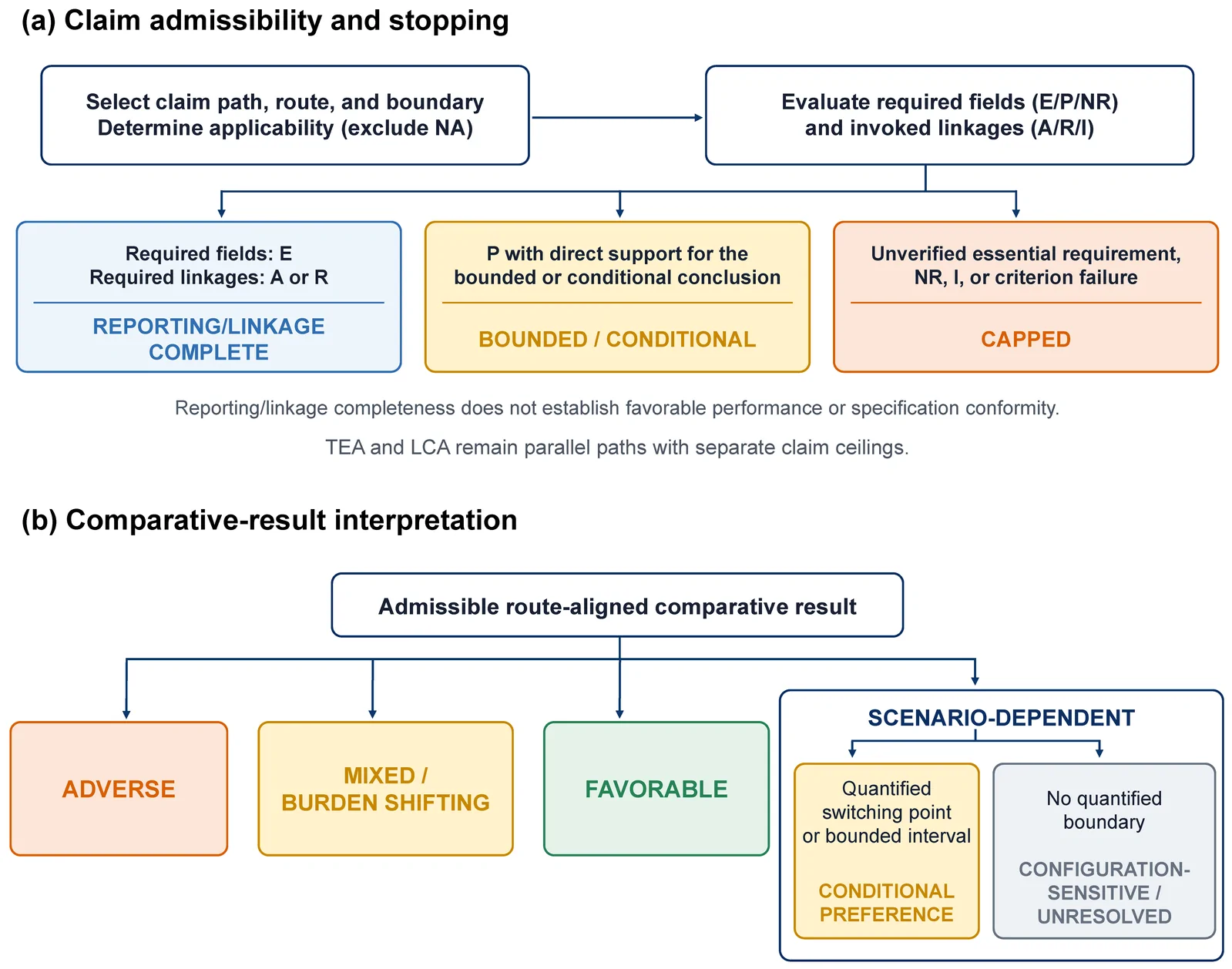
Proposed claim-specific decision logic for evidence requirements and comparative-result interpretation. (**a**) Applicability is determined before the required evidence and linkages are evaluated. Explicit evidence for the required fields (E) and aligned or explicitly reconciled required linkages (A or R) establish reporting completeness and linkage compatibility within the proposed rules. They do not establish favorable performance or fulfillment of an acceptance criterion. Compatibility is assessed for linkages invoked in the specified claim; NL denotes that no linkage was invoked for an eligible evidence record. P permits a bounded or conditional conclusion only when the accessible evidence directly supports that conclusion and no stopping condition applies. An unverified essential requirement, NR, I, or failure of a required criterion limits the affected claim to its last supported conclusion, if any. Economic plausibility and environmental improvement remain parallel paths. (**b**) An admissible comparative result may be adverse, mixed with potential burden shifting, favorable within the stated boundary, or scenario-dependent. A quantified switching point or bounded interval supports conditional preference; without such evidence, preference remains configuration-sensitive or unresolved. Blue indicates reporting/linkage completeness; amber indicates a bounded, mixed, or conditional interpretation; vermilion indicates a capped claim or an adverse result, as labeled; green indicates a favorable comparative result; and gray indicates a configuration-sensitive or unresolved result. E, explicit; P, partial or bounded; NR, required but not reported; NA, not applicable; A, aligned; R, explicitly reconciled; I, incompatible. TEA, techno-economic assessment; LCA, life cycle assessment.

**Figure 3:**
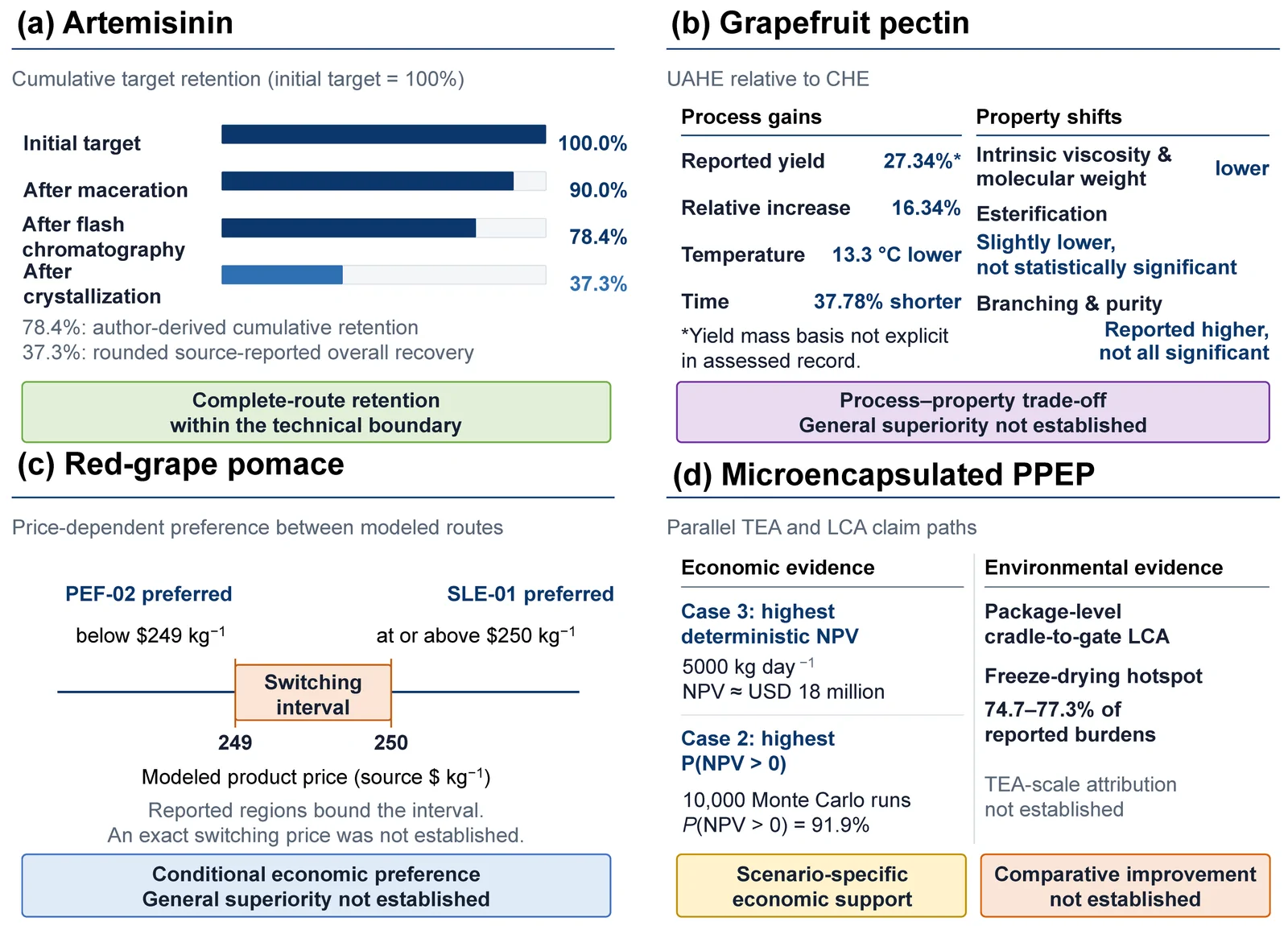
Retrospective illustration of claim interpretation in four selected evidence packages. (**a**) Artemisinin (MI-02): on an initial artemisinin basis of 100%, cumulative retention was 90.0% after maceration, 78.4% after flash chromatography, and 37.3% after crystallization. The intermediate retention is author-derived. The final value rounds the source-reported overall recovery of 37.27% and agrees, at the displayed precision, with the product of the reported conditional recoveries [60]. This arithmetic consistency check does not constitute independent validation. (**b**) Grapefruit pectin (FC-04): ultrasound-assisted heating extraction (UAHE) had a reported yield of 27.34%, a 16.34% relative increase over conventional heating extraction (CHE), with a temperature 13.3 °C lower and an extraction time 37.78% shorter. The yield mass basis was not explicit in the assessed record. Property changes were nonuniform, and not all reported contrasts were statistically significant [62]. (**c**) Red-grape pomace (CC-05): within the source-defined model for a formulated extract at 22 kg per batch (approximately 168 t year^−1^), PEF-02 was preferred below $249 kg^−1^ and SLE-01 at or above $250 kg^−1^. These reported regions bound a switching interval, not an exact switching price. The source dollar notation is retained; currency jurisdiction and monetary base year were not verified in the assessed article [63]. (**d**) Microencapsulated pomegranate-peel extract powder (PPEP; CC-02): Case 3 (5000 kg day^−1^) had the highest reported deterministic net present value (NPV; approximately USD 18 million), whereas Case 2 (100 kg day^−1^) had the highest reported probability of positive NPV, 91.9%, in the source-reported 10,000-run Monte Carlo analysis. These outputs answer different economic questions. The separately scoped life cycle assessment (LCA) identified freeze-drying as a hotspot, contributing 74.7–77.3% of the reported burdens. The lack of a functionally equivalent counterfactual limits comparative environmental inference; the lack of attribution to a specific techno-economic assessment (TEA) scale separately limits integration at that scale [44]. Panels preserve source results while distinguishing author calculations and conditional interpretations. They do not provide a cross-case ranking or independent replicates. SLE-01, solid–liquid extraction reference; PEF-02, pulsed electric field pretreatment scenario.

**Figure 4:**
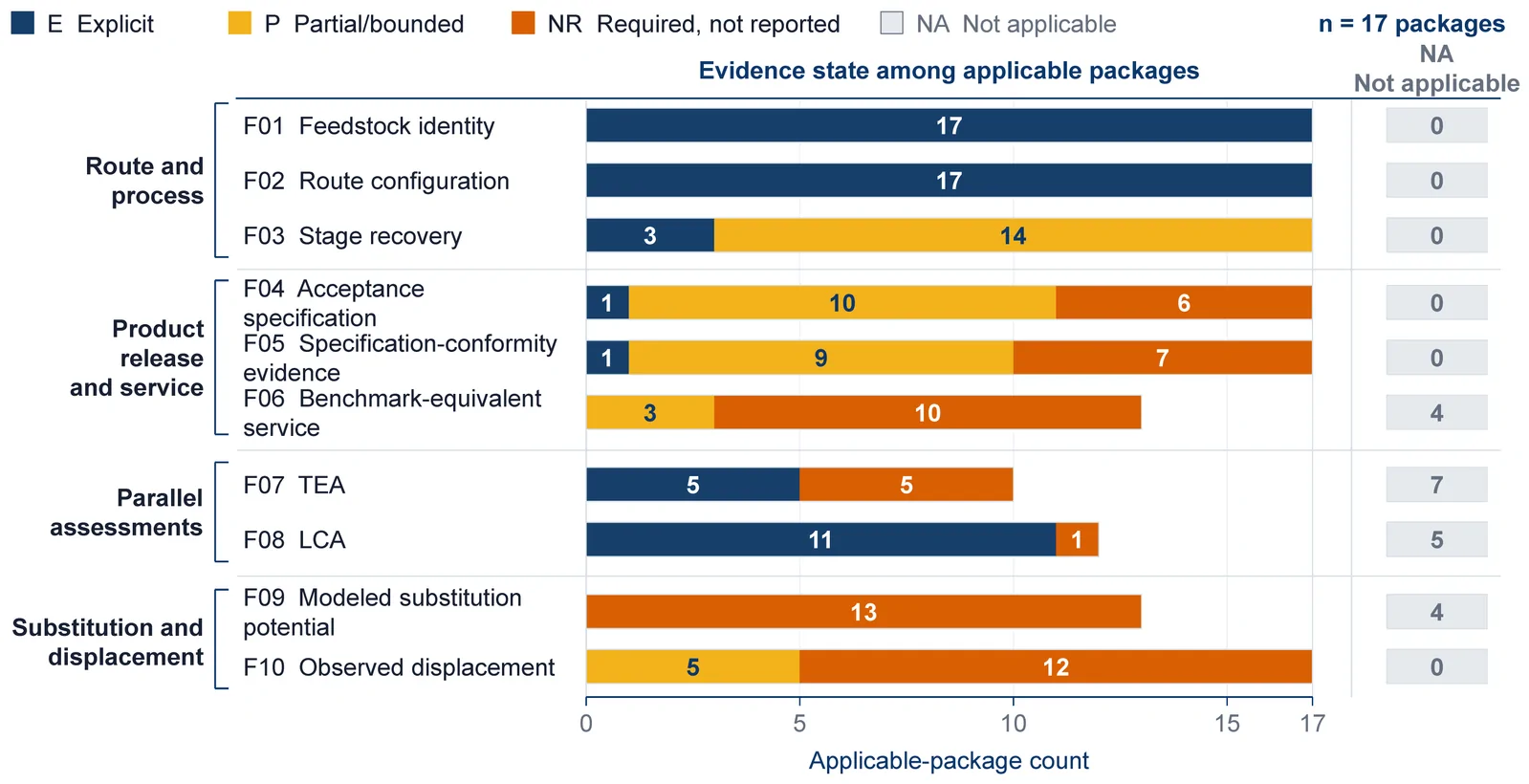
Evidence availability and completeness across 17 author-curated evidence packages. The ten fields comprise 170 package–field decisions. E, explicit; P, partial or bounded, including limitations arising from restricted source access; NR, required for the evaluated claim but not reported in a sufficiently complete accessible record; NA, not applicable to the documented claim scope. Bar lengths are counts, not percentages: each E/P/NR stack totals the field-specific applicable count (17 − NA), with NA shown separately. F05 is displayed as specification-conformity evidence and refers to evidence concerning conformity, not the proportion of products that passed a specification. Explicit evidence can document an unfavorable result. Techno-economic assessment (TEA) and life cycle assessment (LCA) are parallel assessment domains. Observed displacement was examined across all 17 packages as a cross-cutting question. No selected package provided verifiable evidence of displacement at the documented access level: 12 were coded NR within their documented review scope, and five (MI-01, MI-02, MI-03, CC-03, and FC-04) were coded P because accessible material or retained F10 review documentation was insufficient for a definitive nonreporting judgment. These P codes do not indicate partial empirical displacement. Counts describe evidence availability and completeness within the selected set, not product pass rates, route performance, field-wide prevalence, or framework validation. Missing or inaccessible evidence does not establish product failure or absence of displacement.

**Table 1:** Proposed claim-specific evidence requirements and limits on interpretation.

Claim Path	Evidence Required for the Specified Claim	Scope of the Supported Conclusion	Conditions Limiting the Claim
Technical realization	Defined feedstock and route; a reported final chemical or functional endpoint; and evidence that it was achieved under stated conditions. Specification-conforming output additionally requires predeclared acceptance criteria and demonstrated conformity. Methodological background: functionality-based product design [15].	Achievement of the named endpoint or function under the stated conditions; specification-conforming output only for the defined product stream, batch, and configuration when all applicable predeclared acceptance criteria are demonstrably met.	For the named technical endpoint: an undefined measurement basis, unsupported achievement, or incompatible linkage. For specification-conforming output: an undefined product stream or claimed grade; absent final-product mass; missing predeclared acceptance criteria or conformity evidence; or a failed applicable criterion. A post hoc comparison or one favorable attribute does not establish full conformity. These limitations do not erase a separately supported stage-level result.
Physical resource productivity	A defined output state paired with a positive, attributable physical input on matched route, mass, temporal, and system-boundary bases. Equations (7) and (8) specifically use specification-conforming product mass and benchmark-equivalent service, respectively. Product-specific ratios require defensible input assignment; otherwise, retain the complete product basket and shared input profile. Methodological background: functional-unit and boundary principles [19,45,46].	Productivity for the stated output–input pair and boundary. Stage-level ratios retain their stated output basis and do not establish specification-conforming or service-based productivity. Resource inputs remain separate profiles rather than components of an aggregate score.	An unsupported numerator state; mismatched units, moisture bases, periods, or scales; concentration treated as recovery; gross circulation treated as fresh input; missing attribution needed for the named physical input; or unsupported assignment of shared inputs. A zero or negative net input is reported as a balance, not a productivity ratio.
Economic plausibility	Configuration-specific throughput, utilization, balances, CAPEX/OPEX boundary, currency and price year, product-price basis, and decision metric. Quality-control, rejection, waste, residual, and coproduct assumptions are included as applicable. Claims about preference across conditions or robustness additionally require relevant sensitivity or uncertainty evidence. Methodological background: TEA scope and interpretation [19,47].	A deterministic estimate within stated assumptions, a source-reported probabilistic result with its defined interpretation, or a conditional preference within a supported range, break-even condition, or switching interval.	Unsupported grade-dependent pricing; unreconciled scale, currency, or price-year bases; or missing cost assumptions essential to the stated conclusion. Missing sensitivity or uncertainty evidence limits claims of stability or robustness; it does not automatically invalidate a supported point estimate.
Environmental improvement	Functionally equivalent proposed and counterfactual systems with a common functional unit and corresponding reference flows; route-aligned inventories; relevant baseline fate and foregone functions; and stated boundary, multifunctionality treatment, geography, time, and impact-assessment method. Robustness claims additionally require claim-relevant sensitivity or uncertainty evidence. Methodological background: LCA comparison principles [19,45,46].	A lower modeled impact for named categories within the stated comparison and assumptions. A noncomparative inventory or hotspot result remains within its own scope and does not establish environmental improvement.	Unestablished functional equivalence; incompatible inventories or comparison bases; omission of a claim-relevant baseline or foregone function; duplicated credits; unsupported substitution; or extension from one favorable category to overall superiority. Missing uncertainty evidence precludes a robustness claim without necessarily negating a supported point comparison.
Modeled substitution potential	Within the implementation proposed in Equations (4)–(6): specification-conforming output, benchmark-equivalent service, a named comparator and grade, compatible available supply and addressable demand, and an explicit post-cap realization scenario. Baseline fate is included when it affects the modeled replacement amount or an associated environmental comparison. Methodological background: resource potential, recovery, and relative functionality [20].	Scenario-constrained substitution potential under the stated functional, supply, demand, and realization assumptions. This implementation does not define a universal equation for all substitution methods.	Unsupported equal-mass equivalence; total market size used as addressable demand without justification; an unstated or unjustified realization assumption; an unspecified comparator; or a modeled result interpreted as observed displacement. A ratio of one or full realization is not excluded when supported for its stated role; an assumed realization value remains a scenario assumption.
Observed displacement	Defined route and product, named incumbent, and empirical evidence linking recovered-product uptake to reduced incumbent procurement, production, or consumption within a defined relationship and period. Product conformity or service equivalence is additionally required when the claimed identity, grade, function, or replacement amount depends on it. A prior substitution model is unnecessary. Operational definition proposed in this review.	Observed displacement specific to the named product, incumbent, market or supply relationship, and documented period.	Sales, intention, recovered-product volume, addressable demand, modeled substitution, or an LCA credit without an empirical link to reduced incumbent activity.

Notes: CAPEX, capital expenditure; OPEX, operating expenditure; TEA, techno-economic assessment; LCA, life-cycle assessment. The claim classes, conditional requirements, and interpretation limits are proposals of this review. Applicability is determined for the specified claim before evidence status is assigned. The methodological-background citations within the rows support the named principles, not every rule in the corresponding row. Observed displacement is operationalized separately in this review. An unresolved requirement limits the conclusion that depends on it without invalidating a separately supported technical or model-conditional result. Retaining such a result does not establish methodological completeness or compliance with the cited guidance. Explicit evidence and compatible linkages do not establish favorable performance or independent reproducibility.

**Table 2:** Literature-based principles, claim-scope boundaries, and proposed evidence-linkage rules across seven evidence streams.

Evidence Stream	Literature Basis and Claim-Scope Boundary	Proposed Treatment in This Review
Biological source and feedstock identity	Literature basis: Feedstock composition, handling, storage, and safety affect its suitability for processing [6]. Existing uses and the treatment of residual biomass affect the relevant environmental comparison [21,22]. Claim-scope boundary: Generated mass need not equal the mass available to a proposed route; a zero-burden assumption within a defined boundary does not, by itself, account for the consequences of diverting material from another use.	Distinguish generated, genuinely available, and accepted feed on a stated wet- or dry-mass basis. Record incoming-feed criteria, existing uses, and baseline fate; identify burden attribution and any foregone function relevant to the claim.
Green recovery and chemical profiling	Literature basis: Extraction yield, compound-specific measurements, and assay responses characterize different aspects of an extraction process; extraction conditions can affect recovery, selectivity, and composition [17]. Claim-scope boundary: A yield or assay result supports its stated output or attribute; extrapolation to a different product state or to specification conformity requires the corresponding evidence.	Retain the stated basis of extract yield, compositional measurements, and assay responses. Evaluate target-specific recovery when a target constituent is defined. Distinguish recovered target mass, isolated or formulated product mass, and specification-conforming output without requiring a single-target balance for every mixture or function-based product.
Integrated processing, stabilization, and formulation	Literature basis: Separation, drying, formulation, and scale-up can change material recovery, product properties, and process inputs [16,17,18,19]. Claim-scope boundary: Target loss and environmental burden are different outcomes. A stage recovery difference alone does not quantify its environmental consequences or demonstrate performance at another scale.	Link stage-specific outputs, including target retention where applicable and product mass including carriers, to the associated utilities, residuals, and any rejection or reprocessing under the same route key. Record changes in unit operations and scale; require explicit reconciliation when those changes affect the intended linkage.
Product quality, functionality, value, and readiness	Literature basis: Quality and value are multidimensional and context-dependent [50,51]; food formulation can be guided by functional properties rather than purity alone [15]. Claim-scope boundary: A marker, purity value, or functional response supports a specified attribute but does not alone establish all applicable product requirements, equivalent service, or broader economic or societal value.	Identify the product, intended use, grade, and primary archetype while retaining additional applicable requirements. Distinguish characterization, a specification, and demonstrated conformity. For a service-equivalence claim, define the comparator, relevant function, and compatible test and use conditions. Apply the product and output-state distinctions in Section 3.1 as the organizing scheme proposed in this review.
Circularity, recycling quality, and substitution	Literature basis: Circulation, retained function, replacement, and environmental effects have distinct meanings, although existing frameworks can integrate several of these dimensions [20,38]. Claim-scope boundary: Equal-mass or full replacement can be a stated modeling assumption, but the assumption alone does not establish equivalent service or observed displacement.	Retain separate conclusions for circularity, category-specific impacts, modeled substitution, and observed displacement. In the proposed substitution calculation, specify equivalent service, the comparator, available supply, addressable demand, and post-cap realization. Assess observed displacement through the empirical link to reduced incumbent activity, as defined in Table 1.
Techno-economic assessment	Literature basis: TEA guidance addresses scale, utilization, material and energy balances, costs, prices, and uncertainty [19,47]; integrated economic and environmental assessment is also established [40]. Claim-scope boundary: Model conclusions remain conditional on the represented configuration and product grade. A deterministic optimum and a probabilistic risk measure can answer different decision questions.	Check that experimental, product-grade, and economic inputs refer to the modeled configuration or are explicitly reconciled. Record applicable quality-control, rejection, reprocessing, and residual-handling costs, together with price currency and year. Retain reported sensitivity results and supported break-even points or switching intervals without inferring an unreported threshold.
Life-cycle and counterfactual assessment	Literature basis: LCA supports hotspot identification and, when designed for comparison, assessment of environmental differences under defined functional units, boundaries, and modeling choices [19,21,45,46]. Claim-scope boundary: A hotspot result is not a comparative improvement claim; the latter requires a functionally equivalent comparison and an appropriate counterfactual.	State the assessment purpose and check the compatibility of route, function, inventory, and comparison bases. Retain the stated multifunctionality and credit/debit treatment, category-specific results, and relevant uncertainty. Apply the proposed linkage and stopping rules without duplicating credits or combining mutually exclusive scenarios.

Notes: LCA, life-cycle assessment; TEA, techno-economic assessment. The seven streams follow Section 2.1. Row-level citations support the summarized literature basis. The claim-scope boundaries express this review’s interpretation of when a result requires additional evidence to support a broader claim; they do not imply that every cited study made that broader claim. The final column presents the operational treatment proposed in this review. This synthesis is not a comparative performance test or a certification of compliance with the cited standards.

**Table 3:** Purpose-specific comparison of selected review and assessment approaches with the proposed route-level evidence structure.

Approach/References	Primary Unit	Assessment Scope	Treatment of Cross-Scale Linkage	Role of Specification Conformity	Proposed Addition in This Review
Literature-review methodology [41,42,43]	Studies, concepts, and evidence	Question-directed synthesis and conceptual development.	Comparability and transferability follow the chosen appraisal and synthesis.	Product requirements may inform the review question.	Organizes records by route, output state, and claim, with explicit linkage and stopping rules.
Specifications and functionality-driven design [10,15]	Drug substance or product [10]; food formulation [15]	Product-specific acceptance [10]; functionality-based formulation [15].	Processing–performance relationships inform design; acceptance retains its product context.	Tests and criteria define specifications [10]; target function guides formulation [15].	Separates characterization, specification, conformity, and benchmark-equivalent service across the proposed product archetypes.
Scale-up and early-stage assessment [18,19]	Measured or modeled process configuration	Industrial inventory estimates and assessment under maturity and data constraints.	Process-dependent extrapolations link laboratory data to larger-scale scenarios.	Function and performance inform the comparison [19].	Registers scale and evidence type; checks alignment or documented reconciliation of claim-relevant links.
TEA guidance [19,47]	Specified process or product system	Economics conditional on process, cost, price, and uncertainty assumptions.	Scale, utilization, equipment, and balances enter the model and costs.	Application and product basis inform valuation.	Links output grade and quantity to costs and prices; retains distinct deterministic and probabilistic conclusions.
LCA principles and guidance [19,45,46]	Function-defined product system	Inventories, category-specific impacts, and interpretation within goal and scope.	Inventories and assumptions represent scale; ref. [19] addresses early-stage extrapolation.	Function and relevant performance inform comparison.	Checks route, output, inventory, and counterfactual links; distinguishes hotspots from comparative improvement.
Circularity and recycling quality [38,48]	Resource system or recycling pathway	Circularity [48]; substitutability, material use, and environmental dimensions [38].	System boundaries and levels are specified [48]; pathways link material flows [38].	Quality and retained function inform selected indicators [38].	Keeps circularity, conforming output, modeled replacement, and environmental findings separately identifiable.
Substitution reporting [20]	Recovered resource and potentially displaced product	Resource potential, recovery, relative functionality, and expected consumption changes.	Recovery assumptions are linked to end use and displacement scenario.	Relative functionality and applicable constraints inform substitutability.	Specifies service, supply, demand, and realization assumptions; treats observed displacement as a separate empirical claim.
Integrated assessment [39,40]	Ingredient-processing model [39]; process system [40]	Functionality and environmental–economic trade-offs [39]; combined assessment [40].	Linked models share assumptions [39]; ref. [40] applies across production scales.	Functionality is explicit in [39]; requirements follow the assessed system and purpose.	Adds common records and claim-specific interpretation limits while retaining the underlying methods and quality criteria.

Notes: TEA, techno-economic assessment; LCA, life-cycle assessment. This purposive qualitative synthesis is not an exhaustive benchmark, standards-compliance assessment, or performance test. Grouped sources retain their original domains; circularity system level is distinct from process scale. The final column describes proposed evidence organization and operational rules, not demonstrated superiority or features universally absent from other methods. Product-specific guidance is not extended beyond its intended domain.

**Table 4:** Proposed claim-specific records and interpretation limits across scale and evidence settings.

Evidence Setting	Records Needed for the Intended Claim	Supported Scope and Additional Evidence
Laboratory observations	Actual feedstock and conditions; measurement methods, replication and uncertainty; stage denominators and recoveries. Claims about a final product require the relevant separation, concentration, drying or formulation records; conformity claims also require predeclared criteria and batch-level findings.	Measured performance under the reported conditions, or conformity of the assessed batch where documented. A stage result remains useful within that scope; repeated production, larger-scale operation, and routine release require further evidence.
Pilot-scale observations	Actual equipment and effective scale, throughput, operating mode and duration, and feed conditions; linked material and energy balances where relevant. Record scale-sensitive transfer or mixing, recycle, solvent recovery, fouling, cleaning or shutdown, and product variability as applicable.	Operation within the observed conditions and period. Claims of stability or repeatability require corresponding duration or batch records; industrial operation and economic or environmental comparisons require their own evidence.
Modeled industrial configurations	Distinguish measured or literature inputs from assumptions and extrapolations. Specify target capacity and utilization, flowsheet, material and energy balances, equipment scaling, recycle, product losses and off-specification treatment, product and assessment bases, and relevant uncertainty.	Conditional findings for the stated scenario and tested uncertainty range. An industrial-scale model does not document actual plant operation, product release, sales, or observed displacement. Report available model checks and remaining validation limits.
Operating industrial records	Facility, process, period, and throughput; claim-relevant feed, product quality, production, downtime, rejection, reprocessing, utility, and residual records. Distinguish measurements and production or accounting records from estimates and external background data.	The operating or product outcome documented for the observed period. Repeatable quality, sustained profitability, comparative environmental benefit, and incumbent displacement each require evidence specific to that claim.

These author-proposed records are claim-specific, not a validated minimum set, consensus standard, or test of industrialization. The common route record identifies feedstock, process, product state and grade, scale, time basis, and comparison. Scale and evidence type are separate: a pilot-scale model remains modeled evidence, and industrial feedstock does not establish industrial operation of the recovery process. Distinguish observed and modeled components within a source. The settings are illustrative, not a maturity ranking or mandatory progression; no universal throughput cutoff, batch count, or operating duration is proposed. The cases do not longitudinally follow the same route through all four settings. Detailed reporting fields and claim-specific rules remain in Section 2, Section 3 and Section 6 and Supplementary Table S2.

**Table 5:** Domain-level rationale and applicability of the proposed 42-item reporting checklist.

Domain/Items	Central Question	Why the Domain Is Needed	Applicability
D1. Feedstock basis and status (5)	What feed is generated, accessible, and accepted, on which mass and time bases?	Composition, existing uses, and baseline fate affect route inputs and comparisons [2,6,21].	Identify the feed for every route; report mass, time, availability, diversion, and burden bases as required by the claim.
D2. Product specification and conformity (6)	What product is claimed, which criteria apply, and is conformity demonstrated?	Identity, characterization, a specification, and demonstrated conformity support different conclusions [10,12,14].	Identify product state and intended use for every route; assess predeclared acceptance and conformity requirements for the relevant product claim.
D3. Manufacturing configuration (5)	Which operations connect the feed to the stated output?	Separation, formulation, recycling, and scale-sensitive conditions determine what can be linked across records [18,19].	Identify the configuration for every route; specify additional process and balance details needed for the claim.
D4. Scale and reproducibility (4)	At what scale and over which batches or period was the result obtained or modeled?	Scale-up and technology-maturity assumptions affect modeled economic and environmental results [18,19,40].	Record scale and evidence type for every route; repeatability and transfer claims require the corresponding additional evidence.
D5. Techno-economic assessment (5)	Which costs, prices, utilization, and uncertainty assumptions support the economic finding?	Economic conclusions depend on the configuration, product basis, and decision metric [19,47].	Apply to economic claims and economically constrained modeled-substitution analyses; sensitivity or uncertainty requirements depend on the claimed preference or robustness.
D6. Life-cycle assessment (6)	What function, inventories, boundaries, and counterfactual support the environmental conclusion?	A hotspot and a comparative improvement answer different questions; comparison requires compatible systems [20,45,46].	Apply to environmental claims; comparative and robustness claims require evidence beyond a noncomparative result.
D7. Market, substitution, and displacement (6)	What service, available supply, demand, and realization or empirical replacement relationship is supported?	Substitution and recycling-quality frameworks address function and modeled replacement [20,38,52]; this review additionally distinguishes empirical incumbent reduction.	Apply to service-based environmental, modeled-substitution, and observed-displacement claims as relevant; observed displacement does not universally require a prior model.
D8. Multi-output counterfactuals (5)	How are shared inputs, coproduct functions, foregone uses, and credits treated?	Unclear allocations or duplicated replacement credits can change the meaning of a route comparison [21,23,46].	Apply when shared outputs, allocation, baseline fate, or foregone functions affect the claim.

Notes: The item counts sum to 42 and are not weights or scores. Route identification provides a common reporting basis; individual requirements remain claim-dependent, including within the same domain. A domain is not wholly mandatory or wholly inapplicable solely because of its label. Compound items require component-level rationales, and missing information does not justify NA. The references support the named principles; they do not validate this checklist or prescribe all of its rules. Supplementary Table S2 retains the complete items and source mapping.

## Data Availability

No new primary experimental data were generated in this review. Supplementary Table S1 reports the search strategies and documented search execution details. Supplementary Workbook S1 provides candidate-selection and source-access records, literature-derived evidence coding, author-derived calculations, and figure-source data.

## Supplementary Materials

The following supporting information can be downloaded at: https://www.mdpi.com/article/10.3390/molecules31203689/s1, Figure S1: Case-set architecture and levels of inference across 17 author-curated evidence packages and 45 registered analytical records; Figure S2: Claim-specific conclusions in two worked evidence packages; Figure S3: Evidence provenance and linkage compatibility across 17 author-curated evidence packages and ten audit fields; Figure S4: Documented candidate dispositions and the separate role of database coverage checks; Table S1: Database search strategies and retrieval audit used for coverage verification, route-completion audit, and source-status verification; Table S2: Proposed 42-item claim-specific route-reporting checklist; Table S3: Claim-specific decision consequences in four worked cases; Table S4: Boundary-condition evidence packages and claim-specific limitations; Table S5: Glossary of manuscript-specific terms and principal abbreviations; Table S6: Descriptive distribution of recorded package–field availability and completeness codes stratified by documented access to route evidence sources; Supplementary Workbook S1: Evidence-audit workbook containing candidate-selection and source-access records, package and configuration records, claim-path assessments, evidence coding, author-derived calculations, and figure-source data.
