# Supplementary material for: Laboratory-to-Industrial Translation in Plant-Derived Bioproduct Valorization: A Critical Integrative Review and Proposed Framework for Route-Level Evidence Assessment
Source: Molecules. 2026 Oct 15;31(20):3689. doi: 10.3390/molecules31203689 (PMC13707718; doi:10.3390/molecules31203689)
Supplement: Supplementary file 1 [file molecules-31-03689-s001.zip › Supplementary_Figure_Captions.pdf]

Supplementary figure captions

Supplementary Figure S1. Case-set architecture and levels of inference across 17 author-curated evidence packages and 45 registered analytical records. (a) Package distribution by product archetype and analytical role; the classifications describe the same 17 packages. The molecular-identity (MI), composition-constrained (CC), and function-constrained (FC) groups comprise 4, 7, and 6 packages and 5, 27, and 13 analytical records, respectively. Core and boundary-condition roles comprise 11 and 6 packages. (b) Registered analytical records within each package. Counts describe evidence organization, not evidence strength or independent replication. (c) Package-level summaries are distinguished from inference based on separately reported conditions and outcomes. The register contains 17 parent records, 15 variant records, 12 scenario records, and 1 within-source comparison. These are register classes: a parent record can itself describe a modeled case, and the 12 scenario records do not represent all modeled evidence. A package or parent–child link does not establish equivalent feedstocks, products, scales, or system boundaries. In FC-04, two extraction records are linked by a within-source comparison record; that comparison is neither a third extraction route nor evidence of a paired statistical design. All three economic scale records in CC-02, including its parent record, describe modeled cases. Configuration-specific inference requires identifiable source conditions and outcomes. Core and boundary-condition labels indicate analytical roles, not representativeness, evidence quality, validation status, or route superiority.

Supplementary Figure S2. Claim-specific conclusions in two worked evidence packages. MI-02 (artemisinin purification) supports stage-specific recovery and an author-derived intermediate cumulative retention of 78.4%. The final 37.3% rounds the source-reported overall recovery of 37.27% and agrees, at the displayed precision, with the product of the reported conditional recoveries [60]. This is an arithmetic consistency check by the author, not independent validation. Release evidence remains partial. Physical resource productivity, economic plausibility, environmental improvement, and modeled substitution are outside the documented applicability map for this package; observed displacement was evaluated separately and remains unestablished because the available F10 review scope is insufficient for a nonreporting judgment. CC-02 (microencapsulated pomegranate-peel extract powder; PPEP) supports route definition and model-conditional economic findings, while full release conformity remains unestablished. Case 3 has the highest reported deterministic net present value (NPV), whereas Case 2 has the highest reported probability of positive NPV. This review did not calculate a package-level physical resource-productivity ratio; noncalculation does not establish source nonreporting or poor route performance. The package-level life cycle assessment (LCA) supports freeze-drying hotspot identification [44]. The absence of an established functionally equivalent counterfactual limits comparative environmental inference. Separately, unestablished attribution to a specific techno-economic assessment (TEA) scale limits integration at that scale. Modeled substitution was not established. Observed displacement is a separate empirical claim and remains unestablished for both packages. MI-02 is coded P because F10 could not be fully assessed, whereas CC-02 retains NR within its documented review scope. Blue indicates support for the stated result; amber indicates partial release evidence or model-conditional findings; vermilion indicates an unestablished claim or an unevaluated ratio, as labeled; and gray indicates a claim outside documented applicability (NA). These categories describe evidence and claim scope, not route quality.

Supplementary Figure S3. Evidence provenance and linkage compatibility across 17 author-curated evidence packages and ten audit fields. Each panel contains 170 package–field summaries. (a) Provenance is summarized as SR (source-reported information), SR/AD (an author-derived value calculated from traceable source-reported inputs), or MS (a source-reported modeled output); no standalone AD (author-derived evidence involving additional assumptions or constructions) code occurs at the package–field matrix level. A model input or scenario setting is distinguished from a modeled output. Blank provenance cells indicate that no eligible evidence record was included in the matrix. (b) Compatibility is summarized as NL when no cross-source or cross-basis linkage is invoked for eligible evidence, or A when an invoked linkage is aligned within its documented boundary. No R (explicitly reconciled) or I (incompatible) summary codes occur. Blank compatibility cells indicate that no eligible evidence record was available for compatibility classification. NL and a blank entry are not evidence that all possible linkages are compatible. Summaries refer to the claim-relevant output and linkage; they do not assign one code to every underlying input or contextual record. Context-only and gap-rationale records are excluded from positive evidence summaries. F01, feedstock identity; F02, route configuration; F03, stage recovery; F04, acceptance specification; F05, specification-conformity evidence; F06, benchmark-equivalent service; F07, techno-economic assessment; F08, life cycle assessment; F09, modeled substitution potential; F10, observed displacement. F10 remains blank for MI-01, MI-02, MI-03, CC-03, and FC-04 in both panels because their P status in Figure 4 reflects limits in accessible material or retained F10 review documentation rather than verifiable evidence of displacement. F09 is also blank for CC-04 and FC-05 because their contextual or gap-rationale records do not provide eligible evidence for the evaluated substitution claim. Provenance and compatibility are distinct from availability and completeness and do not measure study quality, risk of bias, coding accuracy, or external validity.

Supplementary Figure S4. Documented candidate dispositions and the separate role of database coverage checks. The 30 recorded candidate entries comprise 17 retained evidence packages, five contextual or methodological sources, and eight excluded candidates. The exclusion groups summarize recorded reasons for display; candidate-specific rationales remain in Supplementary Workbook S1. The contextual sources contribute to the discussion and are not counted as exclusions. Later database searches are shown separately because their result sets were neither pooled nor screened as the eligibility corpus. The flow represents documented dispositions, not

an exhaustive discovery or screening history. Verification dates are not initial-discovery dates, and complete candidate-specific discovery histories are unavailable. The architecture of the 17 retained packages and 45 registered analytical records is shown separately in Figure S1.
