# Supplementary material for: Laboratory-to-Industrial Translation in Plant-Derived Bioproduct Valorization: A Critical Integrative Review and Proposed Framework for Route-Level Evidence Assessment
Source: Molecules. 2026 Oct 15;31(20):3689. doi: 10.3390/molecules31203689 (PMC13707718; doi:10.3390/molecules31203689)
Supplement: Supplementary file 1 [file molecules-31-03689-s001.zip › Supplementary_Table_S1.pdf]

Supplementary Table S1. Database search strategies and retrieval audit used for coverage verification, route-completion audit, and source-status verification

Query ID	Database/platform and collection	Evidence stream and audit purpose	Actual search date	Restrictions and limits	Recorded database result count	Export scope and duplicate-handling notes	Audit status / final verification date
**PART A. Direct database searches**							
S1-Scopus-01	Scopus<br>Advanced document search<br>Field: TITLE-ABS	Evidence stream: All substantive streams<br>Audit purpose: Coverage verification, route-completion audit, and source-status verification	11 August 2026	Publication year: None<br>Language: None<br>Document type: None<br>Subject area: None<br>Access status: None<br>Full-text rule: None	A: 1,440<br>B: 57,730<br>C: 3,038<br>D2: 2,179<br>Combined (A OR B OR C OR D2): 63,465	Full 63,465-result export: Not performed<br>Targeted exports: Relevance Top-50 CSV; Mandatory-16 CSV/RIS; Desirable-8 CSV/RIS<br>Cross-database pooling/deduplication: Not applicable; no post-deduplication count calculated	Status: Recorded Scopus audit snapshot<br>Mandatory: 16/16 PASS<br>Desirable: 8/8 PASS<br>Recorded live-run date: 11 August 2026
Exact search string and field code(s)	Field code: TITLE-ABS<br>Query blocks: A OR B OR C OR D2<br>Exact final combined query:<br>(<br>TITLE-ABS(<br>(<br>"reference substance*" OR artemisinin OR "steviol glycoside*" OR curcuminoid*<br>)<br>AND<br>(<br>recover* OR extract* OR isolat* OR purif* OR chromatograph* OR crystalliz* OR "process synthesis"<br>)<br>AND<br>(<br>yield OR recover* OR purity OR preparative OR specification* OR scale-up<br>)<br>)<br>)<br>OR<br>(<br>TITLE-ABS(<br>(<br>pectin OR phenolic* OR polyphenol* OR flavonoid* OR tannin* OR "botanical extract*" OR limonene<br>)<br>AND						

Query ID	Database/platform and collection	Evidence stream and audit purpose	Actual search date	Restrictions and limits	Recorded database result count	Export scope and duplicate-handling notes	Audit status / final verification date

```
  (
    extract* OR recover* OR produc* OR process* OR purif* OR formulat* OR encapsulat* OR valorization OR scale-up
  )
  AND
  (
    "life cycle assessment" OR LCA OR "techno-economic" OR "environmental assessment" OR
    "economic sustainability" OR sustainab* OR functionality OR "functional propert*" OR
    techno-functionality OR specification* OR purity OR yield OR "pilot scale"
  )
)
)
OR
(
TITLE-ABS(
  (
    "agri-food byproduct*" OR "agri-food by-product*" OR "agri-food waste*" OR
    "food byproduct*" OR "food by-product*" OR "food waste*" OR
    "fruit byproduct*" OR "fruit by-product*" OR "fruit waste*" OR
    "side stream*" OR "crop residue*" OR "agricultural residue*" OR
    pomace OR peel* OR bark
  )
  AND
  (
    valorization OR recover* OR extract* OR process* OR fractionat* OR
    formulat* OR scale-up
  )
  AND
  (
    circularity OR "resource productivity" OR "life cycle assessment" OR
    LCA OR "techno-economic" OR "functional unit" OR counterfactual OR
    "baseline fate" OR "system boundary" OR allocation OR substitution OR
    displacement
  )
)
)
OR
(
TITLE-ABS(
  (
```

Query ID	Database/platform and collection	Evidence stream and audit purpose	Actual search date	Restrictions and limits	Recorded database result count	Export scope and duplicate-handling notes	Audit status / final verification date

```
      (
        ("plant material*" OR "natural source*" OR "plant biomass")
        AND
        ("advanced extraction" OR "green extraction" OR (extract* AND "novel method*"))
        AND
        (yield OR purity OR "functional propert*" OR scale-up)
      )
      OR
      (
        ("agri-food byproduct*" OR "agri-food by-product*" OR
         "agrifood byproduct*" OR "agrifood by-product*" OR
         "food by-product*" OR "fruit by-product*" OR
         "fruit waste*" OR "food waste*")
        AND
        (valorization OR utilisation OR utilization OR "integrated processing")
        AND
        (extract* OR fractionat* OR formulat* OR
         "value-added product*" OR "functional ingredient*" OR
         "bioactive ingredient*")
      )
      OR
      (
        ("plant-based ingredient*" OR "food ingredient*")
        AND
        (fractionat* OR functionality OR formulation)
        AND
        (environmental OR economic OR sustainab*)
      )
    )
  )
)
```

Query ID	Database/platform and collection	Evidence stream and audit purpose	Actual search date	Restrictions and limits	Recorded database result count	Export scope and duplicate-handling notes	Audit status / final verification date
S1-WoS-01	Web of Science Core Collection Advanced Search > Fielded Search Query Builder > Session Queries<br>Editions: All<br>Fields: Title OR Abstract	Evidence stream: All substantive streams<br>Audit purpose: Coverage verification, route-completion audit, and source-status verification	12 August 2026	Publication year: None<br>Language: None<br>Document type: None<br>Subject area: None<br>Access status: None<br>Full-text rule: None	A: 1,022<br>B: 44,913<br>C: 2,041<br>D2: 1,382 (Route 1: 66; Route 2: 970; Route 3: 356)<br>Combined: 48,706	Full 48,706-result export: Not performed<br>Targeted export: Mandatory 13 Full Record export; canonical screenshots and logs retained<br>Desirable full-record export: Not present (non-blocking)<br>Cross-database pooling/deduplication: Not applicable	Status: Recorded Web of Science audit snapshot<br>Mandatory: 13/13 PASS among WoS-retrievable anchors; 3 documented Core Collection coverage exceptions<br>Desirable: 8/8 PASS<br>Recorded live-run date: 12 August 2026
Exact search string and field code(s)	Fields: Title OR Abstract (TI/AB); Editions: All<br>Architecture: A OR B OR C OR D2<br>Exact final expanded query:<br>(((TI=("reference substance*" OR artemisinin OR "steviol glycoside*" OR curcuminoid*) OR AB=("reference substance*" OR artemisinin OR "steviol glycoside*" OR curcuminoid*))<br>AND<br>(TI=(recover* OR extract* OR isolat* OR purif* OR chromatograph* OR crystalliz* OR "process synthesis") OR AB=(recover* OR extract* OR isolat* OR purif* OR chromatograph* OR crystalliz* OR "process synthesis"))<br>AND<br>(TI=(yield OR recover* OR purity OR preparative OR specification* OR scale-up) OR AB=(yield OR recover* OR purity OR preparative OR specification* OR scale-up)))<br>OR<br>((TI=(pectin OR phenolic* OR polyphenol* OR flavonoid* OR tannin* OR "botanical extract*" OR limonene) OR AB=(pectin OR phenolic* OR polyphenol* OR flavonoid* OR tannin* OR "botanical extract*" OR limonene))<br>AND<br>(TI=(extract* OR recover* OR produc* OR process* OR purif* OR formulat* OR encapsulat* OR valorization OR scale-up) OR AB=(extract* OR recover* OR produc* OR process* OR purif* OR formulat* OR encapsulat* OR valorization OR scale-up))<br>AND<br>(TI=("life cycle assessment" OR LCA OR "techno-economic" OR "environmental assessment" OR "economic sustainability" OR sustainab* OR functionality OR "functional propert*" OR techno-functionality OR specification* OR purity OR yield OR "pilot scale") OR AB=("life cycle assessment" OR LCA OR "techno-economic" OR "environmental assessment" OR "economic sustainability" OR sustainab* OR functionality OR "functional propert*" OR techno-functionality OR specification* OR purity OR yield OR "pilot scale")))<br>OR<br>((TI=("agri-food byproduct*" OR "agri-food by-product*" OR "agri-food waste*" OR "food byproduct*" OR "food by-product*" OR "food waste*" OR "fruit byproduct*" OR "fruit by-product*" OR "fruit waste*" OR "side stream*" OR "crop residue*" OR "agricultural residue*" OR pomace OR peel* OR bark) OR AB=("agri-food byproduct*" OR "agri-food by-product*" OR "agri-food waste*" OR "food byproduct*" OR "food by-product*" OR "food waste*" OR "fruit						

Query ID	Database/platform and collection	Evidence stream and audit purpose	Actual search date	Restrictions and limits	Recorded database result count	Export scope and duplicate-handling notes	Audit status / final verification date
	byproduct*" OR "fruit by-product*" OR "fruit waste*" OR "side stream*" OR "crop residue*" OR "agricultural residue*" OR pomace OR peel* OR bark))<br>AND<br>(TI=(valorization OR recover* OR extract* OR process* OR fractionat* OR formulat* OR scale-up) OR AB=(valorization OR recover* OR extract* OR process* OR fractionat* OR formulat* OR scale-up))<br>AND<br>(TI=(circularity OR "resource productivity" OR "life cycle assessment" OR LCA OR "techno-economic" OR "functional unit" OR counterfactual OR "baseline fate" OR "system boundary" OR allocation OR substitution OR displacement) OR AB=(circularity OR "resource productivity" OR "life cycle assessment" OR LCA OR "techno-economic" OR "functional unit" OR counterfactual OR "baseline fate" OR "system boundary" OR allocation OR substitution OR displacement)))<br>OR<br>(((TI=("plant material*" OR "natural source*" OR "plant biomass") OR AB=("plant material*" OR "natural source*" OR "plant biomass"))<br>AND<br>((TI=("advanced extraction" OR "green extraction") OR AB=("advanced extraction" OR "green extraction")) OR ((TI=(extract*) OR AB=(extract*)) AND (TI=("novel method*") OR AB=("novel method*"))))<br>AND<br>(TI=(yield OR purity OR "functional propert*" OR scale-up) OR AB=(yield OR purity OR "functional propert*" OR scale-up)))<br>OR<br>((TI=("agri-food byproduct*" OR "agri-food by-product*" OR "agrifood byproduct*" OR "agrifood by-product*" OR "food by-product*" OR "fruit by-product*" OR "fruit waste*" OR "food waste*") OR AB=("agri-food byproduct*" OR "agri-food by-product*" OR "agrifood byproduct*" OR "agrifood by-product*" OR "food by-product*" OR "fruit by-product*" OR "fruit waste*" OR "food waste*"))<br>AND<br>(TI=(valorization OR utilisation OR utilization OR "integrated processing") OR AB=(valorization OR utilisation OR utilization OR "integrated processing"))<br>AND<br>(TI=(extract* OR fractionat* OR formulat* OR "value-added product*" OR "functional ingredient*" OR "bioactive ingredient*") OR AB=(extract* OR fractionat* OR formulat* OR "value-added product*" OR "functional ingredient*" OR "bioactive ingredient*")))<br>OR<br>((TI=("plant-based ingredient*" OR "food ingredient*") OR AB=("plant-based ingredient*" OR "food ingredient*"))<br>AND<br>(TI=(fractionat* OR functionality OR formulation) OR AB=(fractionat* OR functionality OR formulation))<br>AND<br>(TI=(environmental OR economic OR sustainab*) OR AB=(environmental OR economic OR sustainab*)))))						

Query ID	Database/platform and collection	Evidence stream and audit purpose	Actual search date	Restrictions and limits	Recorded database result count	Export scope and duplicate-handling notes	Audit status / final verification date
S1-PubMed-01	PubMed (NCBI) Advanced Search<br>Main field: Title/Abstract [tiab]<br>Identifier gate: [aid]<br>Coverage diagnostic: Title [ti]	Evidence stream: Biomedical/life-science coverage<br>Audit purpose: Coverage verification, route-completion audit, and source-status verification	12 August 2026	Publication date: None<br>Language: None<br>Article type: None<br>Species/sex/age: None<br>Full-text/access rule: None<br>Other filters: None	A: 744<br>B: 17,744<br>C: 694<br>D2: 727<br>Combined: 19,592	Full 19,592-result export: Not performed<br>Targeted exports: Mandatory gate 7-record CSV; Desirable gate 5-record CSV; complete Search History CSV<br>Cross-database pooling/deduplication: Not applicable	Status: Recorded PubMed audit snapshot<br>Mandatory: 7/7 PASS among PubMed-retrievable anchors; 9 AID-set non-retrievals with validated zero-result batch title-signature diagnostic; individual exact-title screenshots not separately archived<br>Desirable: 5/5 PASS among PubMed-retrievable anchors; 3 documented PubMed coverage exceptions
Exact search string and field code(s)	Main field: Title/Abstract [tiab]<br>Architecture: A OR B OR C OR D2<br>Exact final expanded query:<br>(<br>(("reference substance*"[tiab] OR artemisinin[tiab] OR "steviol glycoside*"[tiab] OR curcuminoid*[tiab]) AND (recover*[tiab] OR extract*[tiab] OR isolat*[tiab] OR purif*[tiab] OR chromatograph*[tiab] OR crystalliz*[tiab] OR "process synthesis"[tiab]) AND (yield[tiab] OR recover*[tiab] OR purity[tiab] OR preparative[tiab] OR specification*[tiab] OR "scale-up"[tiab]))<br>)<br>OR<br>(<br>((pectin[tiab] OR phenolic*[tiab] OR polyphenol*[tiab] OR flavonoid*[tiab] OR tannin*[tiab] OR "botanical extract*"[tiab] OR limonene[tiab]) AND (extract*[tiab] OR recover*[tiab] OR produc*[tiab] OR process*[tiab] OR purif*[tiab] OR formulat*[tiab] OR encapsulat*[tiab] OR valorization[tiab] OR "scale-up"[tiab]) AND ("life cycle assessment"[tiab] OR LCA[tiab] OR "techno-economic"[tiab] OR "environmental assessment"[tiab] OR "economic sustainability"[tiab] OR sustainab*[tiab] OR functionality[tiab] OR "functional propert*"[tiab] OR "techno-functionality"[tiab] OR specification*[tiab] OR purity[tiab] OR yield[tiab] OR "pilot scale"[tiab]))<br>)<br>OR<br>(<br>(("agri-food byproduct*"[tiab] OR "agri-food by-product*"[tiab] OR "agri-food waste*"[tiab] OR "food byproduct*"[tiab] OR "food by-product*"[tiab] OR "food waste*"[tiab] OR "fruit byproduct*"[tiab] OR "fruit by-product*"[tiab] OR "fruit waste*"[tiab] OR "side stream*"[tiab] OR "crop residue*"[tiab] OR "agricultural residue*"[tiab] OR pomace[tiab] OR peel*[tiab] OR bark[tiab]) AND (valorization[tiab] OR recover*[tiab] OR extract*[tiab] OR process*[tiab] OR fractionat*[tiab] OR formulat*[tiab] OR "scale-up"[tiab]) AND (circularity[tiab] OR "resource productivity"[tiab] OR "life cycle assessment"[tiab] OR LCA[tiab] OR "techno-						

Query ID	Database/platform and collection	Evidence stream and audit purpose	Actual search date	Restrictions and limits	Recorded database result count	Export scope and duplicate-handling notes	Audit status / final verification date
	economic"[tiab] OR "functional unit"[tiab] OR counterfactual[tiab] OR "baseline fate"[tiab:~0] OR "system boundary"[tiab] OR allocation[tiab] OR substitution[tiab] OR displacement[tiab]))<br>)<br>OR<br>(<br>((("plant material*"[tiab] OR "natural source*"[tiab] OR "plant biomass"[tiab]) AND ("advanced extraction"[tiab] OR "green extraction"[tiab] OR (extract*[tiab] AND "novel method*"[tiab])) AND (yield[tiab] OR purity[tiab] OR "functional propert*"[tiab] OR "scale-up"[tiab])) OR (("agri-food byproduct*"[tiab] OR "agri-food by-product*"[tiab] OR "agrifood byproduct*"[tiab] OR "agrifood by-product*"[tiab] OR "food by-product*"[tiab] OR "fruit by-product*"[tiab] OR "fruit waste*"[tiab] OR "food waste*"[tiab]) AND (valorization[tiab] OR utilisation[tiab] OR utilization[tiab] OR "integrated processing"[tiab]) AND (extract*[tiab] OR fractionat*[tiab] OR formulat*[tiab] OR "value-added product*"[tiab] OR "functional ingredient*"[tiab] OR "bioactive ingredient*"[tiab])) OR (("plant-based ingredient*"[tiab] OR "food ingredient*"[tiab]) AND (fractionat*[tiab] OR functionality[tiab] OR formulation[tiab]) AND (environmental[tiab] OR economic[tiab] OR sustainab*[tiab]))))<br>)						
PubMed Search Details translation	(("reference substance*"[Title/Abstract] OR "artemisinin"[Title/Abstract] OR "steviol glycoside*"[Title/Abstract] OR "curcuminoid*"[Title/Abstract]) AND ("recover*"[Title/Abstract] OR "extract*"[Title/Abstract] OR "isolat*"[Title/Abstract] OR "purif*"[Title/Abstract] OR "chromatograph*"[Title/Abstract] OR "crystalliz*"[Title/Abstract] OR "process synthesis"[Title/Abstract]) AND ("yield"[Title/Abstract] OR "recover*"[Title/Abstract] OR "purity"[Title/Abstract] OR "preparative"[Title/Abstract] OR "specification*"[Title/Abstract] OR "scale-up"[Title/Abstract])) OR (("pectin"[Title/Abstract] OR "phenolic*"[Title/Abstract] OR "polyphenol*"[Title/Abstract] OR "flavonoid*"[Title/Abstract] OR "tannin*"[Title/Abstract] OR "botanical extract*"[Title/Abstract] OR "limonene"[Title/Abstract]) AND ("extract*"[Title/Abstract] OR "recover*"[Title/Abstract] OR "produc*"[Title/Abstract] OR "process*"[Title/Abstract] OR "purif*"[Title/Abstract] OR "formulat*"[Title/Abstract] OR "encapsulat*"[Title/Abstract] OR "valorization"[Title/Abstract] OR "scale-up"[Title/Abstract]) AND ("life cycle assessment"[Title/Abstract] OR "LCA"[Title/Abstract] OR "techno-economic"[Title/Abstract] OR "environmental assessment"[Title/Abstract] OR "economic sustainability"[Title/Abstract] OR "sustainab*"[Title/Abstract] OR "functionality"[Title/Abstract] OR "functional propert*"[Title/Abstract] OR "techno-functionality"[Title/Abstract] OR "specification*"[Title/Abstract] OR "purity"[Title/Abstract] OR "yield"[Title/Abstract] OR "pilot scale"[Title/Abstract])) OR (("agri food byproduct*"[Title/Abstract] OR "agri food by product*"[Title/Abstract] OR "agri food waste*"[Title/Abstract] OR "food byproduct*"[Title/Abstract] OR "food by product*"[Title/Abstract] OR "food waste*"[Title/Abstract] OR "fruit byproduct*"[Title/Abstract] OR "fruit by product*"[Title/Abstract] OR "fruit waste*"[Title/Abstract] OR "side stream*"[Title/Abstract] OR "crop residue*"[Title/Abstract] OR "agricultural residue*"[Title/Abstract] OR "pomace"[Title/Abstract] OR "peel*"[Title/Abstract] OR "bark"[Title/Abstract]) AND ("valorization"[Title/Abstract] OR "recover*"[Title/Abstract] OR "extract*"[Title/Abstract] OR "process*"[Title/Abstract] OR "fractionat*"[Title/Abstract] OR "formulat*"[Title/Abstract] OR "scale-up"[Title/Abstract]) AND ("circularity"[Title/Abstract] OR "resource productivity"[Title/Abstract] OR "life cycle assessment"[Title/Abstract] OR "LCA"[Title/Abstract] OR "techno-economic"[Title/Abstract] OR "functional unit"[Title/Abstract] OR "counterfactual"[Title/Abstract] OR "baseline fate"[Title/Abstract:~0] OR "system boundary"[Title/Abstract] OR "allocation"[Title/Abstract] OR "substitution"[Title/Abstract] OR "displacement"[Title/Abstract])) OR ((("plant material*"[Title/Abstract] OR "natural source*"[Title/Abstract] OR "plant biomass"[Title/Abstract]) AND ("advanced extraction"[Title/Abstract] OR "green extraction"[Title/Abstract] OR ("extract*"[Title/Abstract] AND "novel method*"[Title/Abstract])) AND ("yield"[Title/Abstract] OR "purity"[Title/Abstract] OR "functional propert*"[Title/Abstract] OR "scale-up"[Title/Abstract])) OR (("agri food byproduct*"[Title/Abstract] OR "agri food by product*"[Title/Abstract] OR "agrifood byproduct*"[Title/Abstract] OR "agrifood by product*"[Title/Abstract] OR "food by product*"[Title/Abstract] OR "fruit by product*"[Title/Abstract] OR "fruit waste*"[Title/Abstract] OR "food waste*"[Title/Abstract]) AND ("valorization"[Title/Abstract] OR "utilisation"[Title/Abstract] OR "utilization"[Title/Abstract] OR "integrated processing"[Title/Abstract]) AND ("extract*"[Title/Abstract] OR "fractionat*"[Title/Abstract] OR "formulat*"[Title/Abstract] OR "value added product*"[Title/Abstract] OR "functional ingredient*"[Title/Abstract] OR "bioactive ingredient*"[Title/Abstract])) OR (("plant based ingredient*"[Title/Abstract] OR						

Query ID	Database/platform and collection	Evidence stream and audit purpose	Actual search date	Restrictions and limits	Recorded database result count	Export scope and duplicate-handling notes	Audit status / final verification date
	"food ingredient*"[Title/Abstract]) AND ("fractionat*"[Title/Abstract] OR "functionality"[Title/Abstract] OR "formulation"[Title/Abstract]) AND ("environmental"[Title/Abstract] OR "economic"[Title/Abstract] OR "sustainab*"[Title/Abstract])))						
**PART B. Complementary citation and source-status audit**							
S1-SourceVerification-01	Final source verification and targeted source-resolution checking	Evidence stream: Load-bearing and supporting references across all seven streams<br>Audit purpose: Bibliographic identity, claim–source alignment, numerical fidelity, evidence locators, and publication-status verification	Final verification date: 26 August 2026	Access rule: Official DOI, publisher, Crossmark, PubMed/PMC, and lawful full-text sources where accessible; inaccessible Tier 1 full texts documented as verification gaps	Not applicable	Duplicate handling: DOI/title normalization; withdrawn duplicate records distinguished from current cited articles; no pooled citation-network corpus	Status: 15 full-text checked; 1 official unedited full text; 22 access-limited.
Audit procedure and field code(s)	Official DOI, publisher, Crossmark, PubMed/PMC, and available full-text verification of retained sources. Targeted reference-list or cited-by checks were used only where necessary to resolve a specific claim–source gap; no exhaustive seed-by-seed citation-network search was undertaken. Field code(s): Not applicable.						
S1-Status-01	Official publisher and DOI landing pages	Evidence stream: Publication-status verification<br>Audit purpose: Final source-status verification	Recorded verification date: 2026-08-13	Restrictions/access rule: Official/public publisher and DOI pages	Not applicable	Duplicate handling: Not applicable	Audit status: Web metadata and publication status were checked on 13 August 2026; the recorded status applies to that verification date.
Audit procedure and field code(s)	DOI landing-page and official publisher-status check. Field code(s): Not applicable.<br>Note: at the recorded verification date, one case-evidence record was retained as an official publisher-hosted unedited version; no final edited Version of Record was claimed; the recorded status applies to the stated verification date.						
**PART C. Cross-database record-pooling and duplicate-handling boundary**							
Total records exported from Scopus	Not performed (full Scopus result set not exported). Targeted audit exports only: Relevance Top-50; Mandatory-16 CSV/RIS; Desirable-8 CSV/RIS.						
Total records exported from Web of Science Core Collection	Not performed (full Web of Science result set not exported). Targeted Mandatory 13 Full Record export and canonical provenance evidence retained.						
Total records exported from PubMed	Not performed (full PubMed result set not exported). Targeted Mandatory 7 CSV, Desirable 5 CSV, and complete Search History CSV retained.						

Query ID	Database/platform and collection	Evidence stream and audit purpose	Actual search date	Restrictions and limits	Recorded database result count	Export scope and duplicate-handling notes	Audit status / final verification date
Combined exported records before duplicate reconciliation			Not calculated; the full platform-specific result sets were not pooled.				
Duplicate-handling tool or method			Not applicable at the pooled cross-database record level. The searches were retained separately as platform-specific coverage-verification and route-audit snapshots.				
Within-database duplicates removed			Not calculated; no pooled record-level reconciliation was performed.				
Cross-database duplicates removed			Not calculated; no pooled cross-database corpus was created.				
Unresolved possible duplicates			Not assessed as a pooled set because the complete result sets were not combined.				
Final unique records after duplicate reconciliation			Not calculated; no post-deduplication eligibility denominator was created.				
Reconciliation date			Not applicable for record-level reconciliation. Search-documentation synchronization completed on 12 August 2026.				

1. The searches were used for coverage verification, route-completion audit, and source-status verification within a critical integrative review. They were not intended to define a PRISMA screening universe, an exhaustive field-wide denominator, or the chronological origin of every source considered in the synthesis.
2. The recorded reproducibility searches verified predefined concepts and coverage. Retrieval counts are platform-specific audit snapshots recorded on the stated search dates and are not interpreted as estimates of field-wide prevalence or as an eligibility-screening denominator.
3. The original searches and source-status checks were conducted on 11–13 August 2026; the access status of CC-01 and MI-01 was reverified on 26 August 2026. Fifteen sources were checked in full text, one additional source was checked in official publisher-hosted unedited full text, and 22 sources were constrained to official abstracts, metadata, or other accessible records. The unedited source was treated as citable early-sharing content subject to further editorial change, not as a Version of Record. Records without accessible full text were not classified as fully verified, and coding was limited to what the accessible record directly supported. Targeted reference-list or cited-by checks resolved specific gaps; no exhaustive seed-by-seed citation-network search was undertaken.
4. "None" indicates that no user-applied restriction was used for the specified category. Database coverage, institutional access, open-access status, and full-text availability are not interchangeable concepts.
5. Cross-database duplicate reconciliation was not applicable because the complete Scopus, Web of Science Core Collection, and PubMed result sets were not pooled into a record-level screening corpus. No combined pre-deduplication total, duplicate count, or post-deduplication unique-record count was calculated.
6. Platform-specific search fields and processing differ across Scopus, Web of Science Core Collection, and PubMed. Exact search strings, field codes or tags, settings, dates, and retrieval counts were therefore copied from the final provenance packages rather than harmonized into a common syntax.
