# Supplementary material for: Laboratory-to-Industrial Translation in Plant-Derived Bioproduct Valorization: A Critical Integrative Review and Proposed Framework for Route-Level Evidence Assessment
Source: Molecules. 2026 Oct 15;31(20):3689. doi: 10.3390/molecules31203689 (PMC13707718; doi:10.3390/molecules31203689)
Supplement: Supplementary file 1 [file molecules-31-03689-s001.zip › Supplementary_Table_S2.pdf]

Supplementary Table S2. Proposed 42-item claim-specific route-reporting checklist. The checklist is intended for use according to product archetype and the specified claim, rather than as a uniform requirement for every study. Applicable items form a candidate reporting set, not a consensus-derived standard, certification instrument, or validated guideline. The retrospective assessment reported in this review did not constitute complete application of all 42 items to every configuration or nested analytical record; its implementation scope is described in Sections 2.2–2.5 and 5.5.

Domain	Item ID	Reporting item	Information to report	Applicability by archetype and claim path	Preferred basis, unit, or evidence form	Audit implication if absent, partial, or incompatible	Basis / sources
**Domain 1. Feedstock basis and status**							
D1	D1.1	**Feedstock identity and generation point**	Report the biological source, species or taxon, tissue or material fraction, dedicated-crop or processing-side-stream status, geographic or organizational source, generation point, and primary-processing history. Assign a lot, batch, or source identifier sufficient to distinguish materially different feedstocks.	All routes; CL01–CL06.	Species or taxon; tissue or fraction; source location; generation-point description; lot, batch, or source ID.	P limits conclusions to the described material. NR stops route-level claims that require defined feedstock identity. I prevents linkage to experimental, TEA, LCA, or market evidence for a different material.	[2,21].
D1	D1.2	**Feedstock condition and preprocessing**	Specify moisture or solids content, physical condition, storage time and temperature, stabilization, cleaning, size reduction, prior extraction or fermentation, and relevant safety or contamination status before the assessed route begins. Identify any condition that changes composition, recoverability, or usability.	All routes; CL01–CL06.	Moisture or dry matter (%); storage time and temperature; preprocessing sequence; contaminant or safety test record.	P confines conclusions to the reported condition. NR limits reproducibility and may cap downstream route linkage. I applies when experimental and modeled sources use materially different feedstock states without reconciliation.	[2,6].
D1	D1.3	**Mass basis and system boundary**	State whether every feed and output quantity is reported on a wet, dry, as-received, target-mass, or other basis. Define the temporal and system boundaries used to quantify generated, available, accepted, and processed feed, and document all conversions between bases. Convert any initial target concentration to kilograms of target per kilogram of accepted feed on the declared wet- or dry-mass basis before route accounting.	All routes; CL01–CL05; conditional for CL06 only when the mass basis or temporal boundary is load-bearing.	kg wet basis; kg dry basis; dry-matter fraction; defined period; system-boundary diagram; conversion record.	A documented, unresolved wet/dry-mass or boundary mismatch is I when the combined claim depends on that basis. A traceable conversion may support R; insufficient information leaves the linkage unverified rather than establishing I. An unverified required basis limits the dependent resource-productivity, economic, environmental, or modeled-substitution claim. It does not by itself stop CL06 when that basis is not essential to the documented empirical displacement claim.	[21,46,48].

Domain	Item ID	Reporting item	Information to report	Applicability by archetype and claim path	Preferred basis, unit, or evidence form	Audit implication if absent, partial, or incompatible	Basis / sources
**Domain 1. Feedstock basis and status**							
D1	D1.4	**Availability and incoming-feed acceptance**	Quantify generated feed, genuinely available (route-accessible) feed, and accepted feed separately. Report binding existing uses or commitments, collection losses, seasonality, storage and transport constraints, incoming-feed criteria, and the basis used to avoid double counting. State whether diversion foregoes a beneficial function and whether route accessibility carries environmental or economic opportunity cost.	All routes when generation or supply is extrapolated; CL01–CL05; conditional for CL06 only when feed supply is load-bearing.	Mass generated per period; availability fraction; incoming acceptance fraction; collection and storage assumptions; acceptance rule.	P permits only a bounded supply or route conclusion. NR stops claims based on accepted-feed quantity, scale, physical resource productivity, addressable supply, or modeled substitution potential; it does not by itself stop CL06 when empirical incumbent reduction is documented.	[21,22].
D1	D1.5	**Existing uses, baseline fate, and burden origin**	Identify current beneficial uses, disposal or residual-management pathways, the fraction assigned to each fate, and whether the feed carries upstream burdens, collection burdens, or avoided-disposal credits. State the counterfactual used if diversion changes an existing function or management obligation.	Processing side streams and comparative claims; CL02 and CL04–CL05; conditional for CL06 only when baseline fate is load-bearing.	Baseline-fate shares; existing-use evidence; burden-allocation rule; counterfactual description; displaced-function record.	NR or I prevents comparative environmental and modeled-substitution claims. A zero-burden assumption is not defensible solely because the material is called waste, residue, or by-product. This item is not a universal prerequisite for CL06.	[20,23,46].

Domain	Item ID	Reporting item	Information to report	Applicability by archetype and claim path	Preferred basis, unit, or evidence form	Audit implication if absent, partial, or incompatible	Basis / sources
**Domain 2. Product specification and conformity**							
D2	D2.1	**Product archetype, intended use, and grade**	Classify the product as molecular-identity (MI), composition-constrained (CC), or function-constrained (FC). State the intended use, claimed grade, regulatory or commercial context, and the attributes that are load-bearing for that use. Do not use the archetype as a maturity or value ranking.	All routes; CL01–CL06.	MI, CC, or FC code; intended-use statement; claimed grade; applicable specification anchor.	P limits interpretation to the reported use or grade. NR prevents assignment of a defensible comparator, price, functional unit, or substitution credit. I applies when linked evidence concerns a different grade or use.	[10,12,14].

Domain	Item ID	Reporting item	Information to report	Applicability by archetype and claim path	Preferred basis, unit, or evidence form	Audit implication if absent, partial, or incompatible	Basis / sources
**Domain 2. Product specification and conformity**							
D2	D2.2	**Output numerator and product state**	Name and quantify each decision-relevant output state, distinguishing target mass, isolated-product mass, final formulated-product mass, specification-conforming output, and benchmark-equivalent service. Identify the numerator used in each yield, productivity, TEA, LCA, or substitution calculation.	All routes; CL01–CL06.	Mass of target; mass of isolated product; mass of final product; conforming mass; service-equivalent unit.	P permits only the explicitly supported output state. NR stops claims that require a later output state. I prevents aggregation when experimental, economic, environmental, or market evidence uses different numerators without reconciliation.	[9,20].
D2	D2.3	**Identity, composition, or functional endpoint**	Report the analytical or performance evidence that defines the product: target identity and purity for MI products; marker ranges, fingerprints, composition, and contaminants for CC products; or benchmarked function under defined test conditions for FC products. Report the method, uncertainty, and sample state.	MI, CC, or FC as applicable; CL01–CL05; conditional for CL06 only when the technical endpoint is load-bearing for product identity or the claimed displacement.	Assay or purity; fingerprint or marker profile; composition; functional test result; analytical method; uncertainty.	P supports only a bounded technical-realization claim. NR stops the corresponding technical or product claim. I applies when methods, sample states, or performance endpoints are not comparable across linked sources; this item limits CL06 only when load-bearing for the defined product-displacement claim.	[11,12,14].
D2	D2.4	**Predeclared acceptance specification**	Report the numerical range, limit, grade, fingerprint criterion, functional requirement, or pass/fail rule that was defined before the batch result was interpreted. Identify every applicable criterion and distinguish an intended-use-specific specification from a post hoc literature or commercial comparison.	Routes claiming specification-conforming output; claim-specific for CL01–CL05 and conditional for CL06 only when product grade or specification is load-bearing.	Predeclared limits or ranges; specification document; test method; decision rule; intended-use basis.	A post hoc benchmark is P at most and is not a declared specification. NR stops specification-conforming-output claims and any later claim for which released product grade is load-bearing.	[10,12,13].

Domain	Item ID	Reporting item	Information to report	Applicability by archetype and claim path	Preferred basis, unit, or evidence form	Audit implication if absent, partial, or incompatible	Basis / sources
**Domain 2. Product specification and conformity**							
D2	D2.5	**Batch conformity and material disposition**	Report the measured result for each predeclared criterion, the batch-level pass/fail decision, and the mass of conforming and nonconforming material. Document rejection, downgrading, reprocessing, blending, or other disposition and show how each disposition affects the reported output numerator.	Routes claiming specification-conforming output; claim-specific for CL01–CL05 and conditional for CL06 only when product grade or specification is load-bearing.	Batch result versus criterion; conforming fraction; rejected or reprocessed mass; disposition record.	P identifies incomplete conformity evidence, not a partial batch pass or a numerical conforming fraction. A criterion-specific result supports only that criterion; it does not establish conformity with all applicable acceptance criteria. Missing or inaccessible required evidence leaves batch conformity unestablished and is distinct from demonstrated failure. I applies only to a documented, unresolved incompatibility in an essential linkage. A model-assumed conformity rate remains conditional and cannot be presented as observed batch conformity.	[10,12,13].
D2	D2.6	**Repeat release, stability, and safety**	Report the number of independent batches assessed against the same specification, lot-to-lot results, stability protocol and time points, safety or contaminant evidence, and any documented commercial or operational release. Keep stability and safety conclusions separate from identity, composition, or function.	Conditional for routine-release and scale-up claims; not a universal prerequisite for CL06.	Independent batch count; lot results; stability time points; contaminant or safety record; release documentation.	One conforming batch does not establish repeatability or routine release. NR caps repeat-release and scale-up claims; it limits CL06 only when routine release is load-bearing for the defined displacement claim.	[12–14].

Domain	Item ID	Reporting item	Information to report	Applicability by archetype and claim path	Preferred basis, unit, or evidence form	Audit implication if absent, partial, or incompatible	Basis / sources
**Domain 3. Manufacturing configuration**							
D3	D3.1	**Complete unit-operation sequence**	Report the ordered route from accepted feed to final product, including extraction, separation, purification, concentration, drying, formulation, packaging, recycle, cleaning, and quality-control steps where applicable. Assign stable step and configuration identifiers that can be reused across experiments, TEA, LCA, and market models.	All routes; CL01–CL06.	Process-flow diagram; step IDs; configuration ID; input-output links; source cross-reference.	P permits only a configuration-specific endpoint. NR prevents complete-route yield, cost, impact, and substitution claims. I applies when linked sources omit, add, or replace load-bearing unit operations without reconciliation.	[18,19].
D3	D3.2	**Operating conditions and equipment**	Specify equipment type and scale, solvent or reagent identity, feed-to-solvent ratio, temperature, pressure, pH, time, power or field conditions, mixing, separation settings, drying conditions, and other parameters needed to identify and reproduce each unit operation.	All routes; CL01–CL06.	Equipment model or type; operating ranges; solvent or reagent; temperature; time; pH; pressure; power.	P limits conclusions to the reported settings. NR reduces reproducibility and may make configuration linkage impossible. I applies when modeled and experimental routes use materially different operating conditions without documented conversion.	[18,19].
D3	D3.3	**Stage recovery and denominator**	For each recoverable target or constituent, report the mass entering and leaving every recovery operation, the conditional recovery relative to the target entering that stage, and the evidence that target identity is preserved. Multiply sequential recoveries only when denominators are conditionally nested and compatible.	MI and target-defined CC; conditional for FC; CL01–CL05; conditional for CL06 only when recovery is load-bearing.	Target mass by stage; conditional recovery (%); mass balance; denominator statement; calculation record.	P supports only the reported stage or a bounded cumulative value. NR stops complete-route recovery claims. I prevents multiplication or aggregation when target identity or stage denominators are incompatible. Recovery reporting is not a universal prerequisite for CL06.	[9,11].
D3	D3.4	**Final product mass and formulation effects**	Report the isolated-product mass before release assessment and the final formulated-product mass after carrier addition, dilution, blending, concentration, or other formulation changes. Quantify added carrier or diluent and retain separate records for target mass, total solids, and final product mass.	All routes for output-based claims; CL01–CL05; conditional for CL06 only when final-product mass is load-bearing.	kg isolated product; kg carrier or diluent; kg final product; target concentration; formulation record.	P limits conclusions to the reported mass state. NR prevents specification-conforming-output, physical-resource-productivity, TEA, LCA, and modeled-substitution calculations that require final-product mass. I applies when carrier mass is treated as recovered target. This item is not a universal prerequisite for CL06.	[19,20].

Domain	Item ID	Reporting item	Information to report	Applicability by archetype and claim path	Preferred basis, unit, or evidence form	Audit implication if absent, partial, or incompatible	Basis / sources
**Domain 3. Manufacturing configuration**							
D3	D3.5	**Utilities, recycle streams, and residual fates**	Report fresh and recycled water or solvent separately, process energy and utilities, recycle rates and purge losses, rejected material, wastewater, solid residues, co-products, and their treatment or destination. Link these records to the same configuration and product state used in downstream assessments.	All routes; principally CL02–CL05; conditional for CL06 only when the downstream configuration is load-bearing.	kg fresh water; kg fresh-solvent makeup; recycle fraction; kWh or MJ; residual mass and fate; configuration ID.	NR stops physical resource productivity and weakens TEA, LCA, and counterfactual claims. I applies when downstream inventories correspond to a different recycle, utility, or residual-management configuration. This item is not a universal prerequisite for CL06.	[18,19,46].
**Domain**	**Item ID**	**Reporting item**	**Information to report**	**Applicability by archetype and claim path**	**Preferred basis, unit, or evidence form**	**Audit implication if absent, partial, or incompatible**	**Basis / sources**
**Domain 4. Scale and reproducibility**							
D4	D4.1	**Batch size, capacity, and utilization**	Report experimental batch size, modeled or installed capacity, throughput, operating period, number of operating days, capacity utilization, and any ramp-up or downtime assumptions. Distinguish measured throughput from design capacity and from an author-constructed or source-modeled scenario.	All routes; CL01–CL05; conditional for CL06 only to define the empirical route, market, or supply period.	kg per batch; kg per day or year; operating hours; days per year; utilization factor; scenario label.	P supports only the directly documented scale-specific interpretation. An unverified scale or throughput basis limits any claim that depends on it. Different scales, or the absence of a scaling record, do not by themselves establish I; I requires a documented incompatibility in an essential linkage that remains unresolved. A separately scoped model result retains its stated assumptions. CL06 requires scale detail only when it is essential to the empirical claim.	[18,19,40].
D4	D4.2	**Replication, batches, and variability**	Report technical and biological replicate counts, independent batch counts, sampling design, mean and dispersion, observed range, and any batch-to-batch variation. Distinguish repeated measurements from independent production batches and identify the unit used for statistical inference.	Experimental and pilot routes; CL01–CL05; conditional for CL06 only when repeatability is load-bearing.	n; independent batch count; mean; SD, SE, CI, or range; sampling unit; analysis plan.	P supports a bounded single-batch or limited-repeat conclusion. NR prevents claims of reproducibility, repeat conformity, or stable process performance but may leave proof-of-concept or independently documented empirical displacement assessable.	[18,40].

Domain	Item ID	Reporting item	Information to report	Applicability by archetype and claim path	Preferred basis, unit, or evidence form	Audit implication if absent, partial, or incompatible	Basis / sources
**Domain 4. Scale and reproducibility**							
D4	D4.3	**Scale-up basis and changed operations**	State the scaling method, empirical data, engineering correlations, equipment analogues, and assumptions used to move between laboratory, pilot, demonstration, and industrial scales. Identify every unit operation, inventory, product grade, yield, or utility basis changed during scale-up.	Scale-up or modeled routes; CL01–CL05; conditional for CL06 only when scaled-route identity is load-bearing.	Scale factor; scaling equation; equipment basis; changed-step register; transformed inventory; uncertainty range.	P permits only a conditional scale-up conclusion. NR stops route-aligned TEA or LCA claims at the larger scale. I applies when scale-up silently substitutes a different route, drying method, grade, or inventory. This item is not a universal prerequisite for CL06.	[18,19].
D4	D4.4	**Configuration, scenario, and unit of inference**	Identify the parent route configuration, each configuration variant, and each modeled scenario. State the decision variable changed, the source-reported or author-constructed status, and whether inference is supported at package, configuration, scenario, batch, or source level. Do not count nested scenarios as independent studies.	Multiple configurations or scenarios; CL01–CL05; conditional for CL06 only when configuration identity is needed to interpret empirical displacement evidence.	Configuration ID; parent ID; scenario ID; changed variable; provenance; permitted unit of inference.	NR obscures the denominator for coverage and claim decisions. P limits conclusions to the identified configuration or scenario. I applies when nested or materially different configurations are treated as independent or directly interchangeable; this item limits CL06 only when configuration identity is load-bearing.	[19,40].

Domain	Item ID	Reporting item	Information to report	Applicability by archetype and claim path	Preferred basis, unit, or evidence form	Audit implication if absent, partial, or incompatible	Basis / sources
**Domain 5. Techno-economic assessment**							
D5	D5.1	**TEA goal, boundary, scale, and utilization**	State the decision question, analysis boundary, reference production basis, capacity, scale, utilization, operating period, geography, technology readiness, and whether results are source-reported or scenario-modeled. Identify the experimental or process configuration to which the TEA applies.	Routes supporting CL03; conditional for economically constrained CL05 analyses. Not required for CL06.	kg product or service per period; capacity; utilization; geography; study year; configuration ID.	P supports only the economic conclusion directly supported within the documented model scope. Unverified goal, boundary, scale, or utilization information limits the claim that depends on it. I applies when an invoked connection to experimental or other evidence has a documented, unresolved incompatibility in an essential basis. A scale difference alone does not establish I or invalidate a separately scoped model result. TEA is not a universal prerequisite for CL06.	[40,47].

Domain	Item ID	Reporting item	Information to report	Applicability by archetype and claim path	Preferred basis, unit, or evidence form	Audit implication if absent, partial, or incompatible	Basis / sources
**Domain 5. Techno-economic assessment**							
D5	D5.2	**Route-aligned mass and energy balances**	Provide mass and energy balances for the TEA configuration, including feed acceptance, yield, stage losses, recycle, solvent recovery, drying, rejection, co-products, waste, and utilities. Reconcile modeled balances with experimental values and identify values transferred, scaled, or assumed.	Routes supporting CL03.	kg or mol per batch; kg product basis; kWh or MJ; yield and loss fractions; balance closure; source linkage.	P permits only a conditional cost result. NR prevents route-aligned economic plausibility. I applies when costs are based on a mass or energy balance that is incompatible with the reported route or product state.	[19,40,47].
D5	D5.3	**CAPEX, OPEX, and omitted-cost controls**	Report capital and operating cost categories, labor, maintenance, utilities, consumables, quality control, testing, rejection, reprocessing, waste and residual handling, replacement materials, overhead, and contingency. State whether each item is included, excluded, or represented by a factor.	Routes supporting CL03.	Currency and price year; installed capital; annual OPEX; cost per product or service unit; inclusion/exclusion table.	P permits only a bounded estimate. NR for load-bearing cost categories caps CL03. I applies when costs are transferred from a route with a different quality-control, rejection, waste, or utility burden.	[40,47].
D5	D5.4	**Product grade, price basis, and metrics**	Define the product grade and quantity to which the selling price or value is applied. Report currency, price year, geography, market basis, taxes or subsidies if included, and formulas for LCOP, MSP, NPV, IRR, payback, or other metrics. Do not assign purified-product prices to crude outputs.	Routes supporting CL03; comparative economic claims.	Grade-matched price; currency; price year; LCOP, MSP, NPV, IRR, or payback formula; product basis.	P supports only a grade- and scenario-specific conclusion. NR prevents defensible profitability or break-even claims. I applies when the price basis or numerator does not match the modeled product.	[40,47].

Domain	Item ID	Reporting item	Information to report	Applicability by archetype and claim path	Preferred basis, unit, or evidence form	Audit implication if absent, partial, or incompatible	Basis / sources
**Domain 5. Techno-economic assessment**							
D5	D5.5	**Sensitivity, uncertainty, and switching conditions**	Report parameter ranges or distributions, scenario definitions, sensitivity method, uncertainty treatment, and the variables controlling preference. Where direction changes, quantify the break-even condition, switching point, or bounded interval and state the configurations and assumptions on each side of that boundary.	Comparative TEA and conditional preferences; CL03.	Low/base/high scenarios; probability distribution; sensitivity coefficient; break-even value; switching interval.	Assess the evidence for sensitivity, uncertainty, and switching conditions separately from the explicitness of the economic point estimate. An explicit TEA result does not establish that this item is complete, and a point estimate alone is not partial uncertainty evidence. Assign the item's evidence-status code according to the accessible evidence, applicability, and common coding rules. A stable-preference claim requires support across the stated conditions; an unresolved switching boundary limits that claim without invalidating a separately supported model-specific estimate.	[19,40,47].
**Domain 6. Life-cycle assessment**							
D6	D6.1	**Goal, scope, functional unit, and reference flow**	State the LCA goal, intended application, audience, comparative status, functional unit, reference flow, product or service grade, and reason the unit represents the assessed function. Identify any limitation on using equal mass as a proxy for equal service.	Routes supporting CL04 and environmental comparisons linked to CL05. Not required for CL06.	Functional unit; reference flow; product grade; service description; comparative purpose.	P permits only the stated category- and unit-specific environmental result. NR or a non-equivalent functional unit stops comparative environmental-improvement and LCA-based credit claims. This item does not stop CL06.	[20,45,46].

Domain	Item ID	Reporting item	Information to report	Applicability by archetype and claim path	Preferred basis, unit, or evidence form	Audit implication if absent, partial, or incompatible	Basis / sources
**Domain 6. Life-cycle assessment**							
D6	D6.2	**Proposed and counterfactual system boundaries**	Define functionally equivalent proposed and counterfactual systems. Under the direct-difference convention, place the valorization route and any replacement of a foregone beneficial function in the proposed system, and place the baseline feedstock fate or use and incumbent service in the counterfactual. If another credit/debit convention is used, state all assignments and signs explicitly. Show boundary differences.	Comparative LCA; CL04 and LCA-dependent CL05 analyses. Not required for CL06.	Boundary diagram; included and excluded processes; baseline-fate record; incumbent-service system.	NR or I stops CL04 and other LCA-dependent comparative claims. A favorable result under one boundary cannot be generalized to another boundary, baseline fate, or incumbent service. This item does not stop CL06.	[21,23,46].
D6	D6.3	**Route-aligned inventory and data context**	Report the life-cycle inventory for the same feedstock condition, unit-operation sequence, scale, product grade, utilities, recycle, residual fate, and output numerator as the assessed route. Identify primary and secondary data sources, data quality, geography, time period, electricity mix, and technology context.	Routes supporting CL04.	LCI per functional unit; source and year; geography; electricity mix; data-quality assessment; configuration ID.	P permits only a bounded category-specific conclusion. NR prevents route-aligned environmental interpretation. I applies when the inventory is borrowed from an incompatible configuration or scale.	[18,23,46].
D6	D6.4	**Subdivision, allocation, and system expansion**	Report whether subdivision, physical allocation, economic allocation, another allocation rule, or system expansion was used. State allocation factors and their causal or economic basis. For system expansion, identify the substituted product, product grade, substitution ratio, and avoided process.	Multi-output or substitution-sensitive LCA; CL04 and LCA-dependent CL05 analyses. Not required for CL06.	Allocation factors; subdivision record; named substituted product; substitution ratio; avoided-process inventory.	NR for a required allocation or system-expansion rule prevents the dependent comparative environmental claim. P supports only the allocation-specific result directly supported by accessible evidence, subject to the common stopping rules. Unsupported or duplicated credits cannot support the dependent claim. Missing information alone does not establish I; I requires a documented, unresolved incompatibility in an essential comparison or linkage basis. This item does not impose LCA as a prerequisite for CL06.	[20,23,46].

Domain	Item ID	Reporting item	Information to report	Applicability by archetype and claim path	Preferred basis, unit, or evidence form	Audit implication if absent, partial, or incompatible	Basis / sources
**Domain 6. Life-cycle assessment**							
D6	D6.5	**Impact method, category results, and hotspots**	Identify the life-cycle impact-assessment method and version, characterization models, impact categories, normalization or weighting if used, and category-specific results per functional unit. Report principal contributing processes or life-cycle stages and retain favorable and adverse categories separately.	Routes supporting CL04.	LCIA method and version; category-specific impact per functional unit; contribution or hotspot analysis.	NR prevents CL04. P supports only the reported categories and boundary. One favorable category or circularity indicator does not establish overall environmental superiority.	[26,45,46].
D6	D6.6	**Sensitivity, uncertainty, and burden shifting**	Report sensitivity and uncertainty for load-bearing inventory, allocation, counterfactual, substitution, electricity, yield, rejection, and scale assumptions. Identify whether the preferred route changes across scenarios and whether improvements in one impact category or life-cycle stage coincide with adverse changes elsewhere.	Comparative LCA; CL04 and LCA-dependent CL05 analyses. Not required for CL06.	Scenario ranges; uncertainty intervals; alternative allocation or counterfactual; category-and stage-specific direction.	Assess sensitivity, uncertainty, and burden-shifting evidence separately from the explicitness of a deterministic LCA result. Assign the item’s evidence-status code according to the accessible evidence, applicability, and common coding rules. Robustness is limited to the reported sensitivity ranges or scenarios over which the direction remains unchanged; probabilistic outputs require separate interpretation. Direction changes restrict the conclusion to the supported conditions, and an unassessed burden shift cannot support overall superiority. This item does not impose LCA as a prerequisite for CL06.	[23,26,46].

Domain	Item ID	Reporting item	Information to report	Applicability by archetype and claim path	Preferred basis, unit, or evidence form	Audit implication if absent, partial, or incompatible	Basis / sources
**Domain 7. Market, substitution, and displacement**							
D7	D7.1	**Comparator, grade, and defined service**	Name the incumbent or reference product, its grade, intended use, geography, market or supply context, and the service being compared. Explain why the recovered and reference products belong in the same comparison and identify any quality or function difference that affects use.	Comparative claims (CL04–CL05). For CL06, the route and product, named incumbent, relationship, and period must be defined. Service-equivalence evidence is conditional on the function or replacement amount claimed; a prior substitution model is not required.	Named comparator; grade; service definition; use context; geography and period.	NR or I stops service-based comparison and modeled substitution. For CL06, absence of a named incumbent stops the claim, but a prior substitution model is not required.	[20,38,52].
D7	D7.2	**Matched tests and required use levels**	Report the test method, conditions, performance criterion, and amounts of recovered and reference product needed to deliver the same defined service. State whether proportional performance is defensible across the relevant use range and document any nonlinearity, matrix effect, or application limitation.	FC routes and comparative MI or CC routes; CL04–CL05. Conditional for CL06 when the claimed function or replacement amount depends on matched performance or use levels.	Matched test protocol; recovered-product use level; reference-product use level; performance curve; application matrix.	P supports only the application-specific comparison directly supported by accessible evidence. NR or an unresolved required comparison prevents the dependent benchmark-equivalent-service or modeled-substitution claim. I applies to a documented, unresolved incompatibility in a required test or use basis. For CL06, this item limits the claim only when matched function or use levels are essential to the stated displacement amount or interpretation.	[14,20,52]

Domain	Item ID	Reporting item	Information to report	Applicability by archetype and claim path	Preferred basis, unit, or evidence form	Audit implication if absent, partial, or incompatible	Basis / sources
**Domain 7. Market, substitution, and displacement**							
D7	D7.3	**Benchmark-equivalent service and service-equivalence ratio**	Express specification-conforming output in reference-product-equivalent service units and report the recovered-to-reference use ratio or another explicit service-equivalence ratio. Describe the calculation, units, proportionality assumption, and the product and service boundary to which the ratio applies.	CL05 and service-based CL04. Conditional for CL06 when interpreting the claimed replacement amount requires a service-equivalence ratio; a prior substitution model is not required.	Reference-product-equivalent service unit; recovered/reference use ratio; service-equivalence ratio; calculation record.	A service-based claim requires an explicitly supported equivalence basis. P supports only the directly documented scope and does not overcome an unresolved essential requirement. NR for a required equivalence basis prevents the dependent CL05 claim. Equal-mass replacement without the necessary functional evidence does not establish service equivalence. For CL06, this item limits only the displacement claim that depends on that equivalence basis.	[20,38].
D7	D7.4	**Available supply and addressable demand**	Report genuinely available supply after existing uses and acceptance losses, addressable demand expressed in the same benchmark-equivalent service unit, geography, period, market boundary, and data source. Distinguish total theoretical market size from demand that the defined grade and service could address.	Modeled substitution potential; CL05 only. Not required for CL06.	Service-equivalent supply per period; addressable demand per period; geography; market boundary; data source.	P limits modeled substitution potential to the reported market and period. NR stops scenario-constrained substitution potential. A theoretical market size is not evidence of replacement and is not required to establish CL06 from empirical records.	[20,25].
D7	D7.5	**Post-cap realization assumptions and modeled substitution potential**	State the post-cap realization fraction, scenario logic, constraints, time horizon, and whether values are source-modeled or author-constructed. Apply this fraction only after benchmark-equivalent supply and addressable demand have been constrained by the minimum operator. Do not use it to repeat relative functionality, supply, or demand constraints.	Modeled substitution potential; CL05 only. Not a precursor or prerequisite for CL06.	Post-cap realization fraction; modeled period; scenario ID; min(supply, demand) basis; provenance and sensitivity.	P supports only the scenario-constrained result directly supported by accessible evidence and does not overcome an unresolved essential requirement. NR for required realization assumptions prevents the dependent CL05 conclusion. A realization coefficient may be evidence-based or explicitly assumed for a stated scenario; its basis and limits must be documented. Neither zero nor full realization may be assigned by default. An assumed value does not establish observed displacement, and an LCA substitution credit is not observed displacement. This item is not a prerequisite for CL06.	[20,25,28].

Domain	Item ID	Reporting item	Information to report	Applicability by archetype and claim path	Preferred basis, unit, or evidence form	Audit implication if absent, partial, or incompatible	Basis / sources
**Domain 7. Market, substitution, and displacement**							
D7	D7.6	**Observed displacement**	Document a defined route and product and a named incumbent, plus empirical evidence—such as procurement, production, contract, or sales records—linking recovered-product uptake to reduced incumbent procurement, production, or consumption within a defined market or supply relationship and period. Report leakage, additional demand, and rebound where relevant. A prior substitution model is not required.	Observed displacement only; CL06.	Named incumbent; empirical procurement, production, contract, or sales record linking recovered-product uptake to incumbent reduction; defined market or supply relationship and period.	Sales, purchase intention, recovered-product procurement volume, market potential, modeled substitution potential, or an LCA credit alone does not establish CL06. Without an empirical link to reduced incumbent procurement, production, or consumption, observed displacement is not established.	[20,25,28].

Domain	Item ID	Reporting item	Information to report	Applicability by archetype and claim path	Preferred basis, unit, or evidence form	Audit implication if absent, partial, or incompatible	Basis / sources
**Domain 8. Multi-output counterfactuals**							
D8	D8.1	**Complete output and residual inventory**	Identify and quantify all intended products, co-products, intermediates, rejected material, wastewater, emissions, solid residues, recovered utilities, and final treatment pathways. Link every output to the unit operation, mass basis, product state, and configuration from which it arises.	Multi-output routes; CL02–CL05; conditional for CL06 only when multi-output identity is load-bearing.	Mass balance by output; product and residual IDs; quality or grade; final fate; configuration linkage.	P limits conclusions to reported outputs. NR prevents complete resource, economic, environmental, and counterfactual accounting. I applies when outputs are omitted or assigned to a different route basis. This item is not a universal prerequisite for CL06.	[27,46].

Domain	Item ID	Reporting item	Information to report	Applicability by archetype and claim path	Preferred basis, unit, or evidence form	Audit implication if absent, partial, or incompatible	Basis / sources
**Domain 8. Multi-output counterfactuals**							
D8	D8.2	**Subdivision and allocation basis**	Use subdivision where operations and inventories are separable. Otherwise, state the physical, economic, or other allocation rule, allocation factors, causal or economic rationale, price year where relevant, and sensitivity to alternative allocation choices. Keep physical allocation distinct from economic allocation. Product-specific physical resource-productivity ratios require a defensible denominator assignment; otherwise, retain the complete product basket and shared input profile without summing ratios that use the same unallocated denominator.	Multi-output routes; CL02–CL04 and related CL05.	Subdivided inventory; allocation factor; causal physical parameter; economic value and price year; sensitivity case.	NR prevents reproducible route comparison. P permits only an allocation-specific conclusion. I applies when the allocation basis is unrelated to the physical or economic relationship or changes across linked models without reconciliation.	[23,46].
D8	D8.3	**System expansion and substitution details**	When system expansion is used, name the substituted product or service, product grade, production system, geographic and temporal context, substitution ratio, and avoided inventory. Demonstrate service or grade equivalence and report any limit on the quantity that can reasonably displace the incumbent.	Multi-output comparative routes; CL04–CL05. Not required for CL06.	Named substituted product; service or grade; substitution ratio; avoided-process inventory; displacement limit.	P supports only a bounded expanded-system result. NR or I prevents the affected substitution credit. Unsupported full substitution must not establish environmental improvement; neither a credit nor its absence establishes observed displacement.	[20,46].

Domain	Item ID	Reporting item	Information to report	Applicability by archetype and claim path	Preferred basis, unit, or evidence form	Audit implication if absent, partial, or incompatible	Basis / sources
**Domain 8. Multi-output counterfactuals**							
D8	D8.4	**Existing-use and displaced-function counterfactual**	Report the existing use of each diverted feedstock or co-product and the system required to replace that function. Under the direct-difference convention, assign replacement of the foregone function to the proposed expanded system; under another convention, state the alternative debit or credit assignment and its sign. Include changes in residual management, transport, treatment, or upstream production caused by diversion, and state whether the modeled consequence is attributional, consequential, or scenario-specific.	Side-stream and multi-output routes; CL04–CL05. Conditional for CL06 only when baseline fate is part of the empirical claim.	Existing-use share; displaced-function system; replacement inventory; management consequence; scenario type.	NR or I prevents a defensible comparative environmental counterfactual. A side stream with an existing beneficial use cannot receive full avoided-disposal credit without accounting for the displaced function. This item is not a universal prerequisite for CL06.	[21–23].
D8	D8.5	**Credit and burden double-counting control**	Provide a credit-and-burden ledger showing where feedstock burdens, avoided-disposal credits, co-product credits, substitution credits, and residual-treatment burdens are assigned. Within each product-system comparison, partition shared inventory flows and burdens among outputs or retain them within the complete product basket under the declared multifunctionality treatment. Do not credit the same avoided baseline fate or displaced function more than once, and do not sum mutually exclusive scenarios or treat them as independent evidence.	Multi-output and comparative routes; CL02–CL05; conditional for CL06 only when burden or credit accounting is part of the claim.	Credit/burden ledger; allocation or system-expansion map; alternative scenario; sensitivity result.	Any unresolved duplicate assignment is I for the affected resource, economic, environmental, or modeled-substitution claim. NR caps only conclusions for which the burden-and-credit ledger is load-bearing; it does not by itself stop independently documented CL06.	[20,23,46].

Notes: MI, molecular-identity; CC, composition-constrained; FC, function-constrained. CL01, technical realization; CL02, physical resource productivity; CL03, economic plausibility; CL04, environmental improvement; CL05, modeled substitution potential; CL06, observed displacement.

E, explicit evidence for the assessed field or requirement; P, partial or bounded evidence, including limitations arising from restricted source access; NR, required for the evaluated claim but not reported in a sufficiently complete accessible record; NA, not applicable to the defined claim. Explicit reporting does not establish a favorable result or criterion satisfaction. SR, source-reported information, including observed measurements or directly reported conditions; MS, source-reported modeled outputs; SR/AD, an author derivation from traceable source-reported inputs; AD, an author-derived scenario, classification, or other construction introducing author-specified assumptions. A model input or scenario description is not itself a modeled output. Provenance describes the origin or derivation of information, not its quality.

A, aligned; R, explicitly reconciled; I, a documented mismatch in a claim-essential basis that remains unresolved; NL, an eligible evidence record for which no cross-source or cross-basis linkage is invoked. R requires a traceable record of the original and converted bases, transformation, assumptions, and limits. Insufficient information to evaluate an invoked linkage leaves it unresolved rather than establishing I or NL. A blank compatibility entry denotes the absence of an eligible evidence record; neither NL nor a blank entry establishes alignment. ISO, International Organization for Standardization; ICH, International Council for Harmonisation; EMA, European Medicines Agency; JECFA, Joint FAO/WHO Expert Committee on Food Additives.

Applicability is determined before assigning E, P, NR, or NA. P supports a narrower or conditional conclusion only when the accessible evidence directly supports that conclusion and no stopping condition applies. Access-limited P does not establish partial fulfillment of an unverified requirement. An unresolved essential requirement, NR for an applicable requirement, a confirmed incompatible linkage, or failure of a claim-required acceptance or equivalence criterion limits the affected claim to its last supported conclusion, if any. Missing information alone does not justify NA. Evidence for other requirements cannot compensate for an unresolved prerequisite of the same claim.

For CL06, a defined feedstock and route, an identified recovered product and named incumbent, and empirical evidence linking recovered-product uptake to reduced incumbent procurement, production, or consumption within a defined relationship and period are required. A prior substitution model, TEA, or LCA is not a universal prerequisite. Recovery, specification, conformity, and service-equivalence evidence are required when the claimed product identity, grade, function, or replacement amount depends on them. Empirical displacement must not be inferred solely from modeled substitution potential or an LCA credit.

A later TEA, LCA, market model, or adjacent source can fill an evidence gap only if it supplies the information required for the specified claim and that information is linked on aligned or explicitly reconciled bases. Matching route fields or performing a reconciliation cannot create missing feedstock-definition, product-conformity, or service-equivalence evidence. A separately scoped technical or model-conditional finding may remain interpretable within its own documented limits.

The cited sources are representative and non-exhaustive and provide methodological context rather than defining or validating the checklist.

Formalization as a reporting guideline would require stakeholder involvement, independent assessment, prospective application, and evaluation of item clarity, feasibility, decision impact, and user burden.

References cited in Supplementary Table S2

2. Fernandes, F.; Delerue-Matos, C.; Grosso, C. Unveiling the potential of agrifood by-products: A comprehensive review of phytochemicals, bioactivities and industrial applications. Waste and Biomass Valorization 2025, 16, 2715–2748. https://doi.org/10.1007/s12649-024-02622-0

6. Hasan, M.M.; Islam, M.R.; Haque, A.R.; Kabir, M.R.; Khushe, K.J.; Hasan, S.M.K. Trends and challenges of fruit by-products utilization: insights into safety, sensory, and benefits of the use for the development of innovative healthy food: a review. Bioresour. Bioprocess. 2024, 11, 10. https://doi.org/10.1186/s40643-023-00722-8

9. Duewer, D.L.; Parris, R.M.; White, E.; May, W.E.; Elbaum, H. An approach to the metrologically sound traceable assessment of the chemical purity of organic reference materials; NIST Special Publication 1012; National Institute of Standards and Technology: Gaithersburg, MD, USA, 2004. https://doi.org/10.6028/NIST.SP.1012

10. International Council for Harmonisation. ICH Q6A: Specifications: Test procedures and acceptance criteria for new drug substances and new drug products: Chemical substances; ICH: Geneva, Switzerland, 1999.

11. Westwood, S.; Lippa, K.; Shimuzu, Y.; Lalerle, B.; Saito, T.; Duewer, D.; Dai, X.; Davies, S.; Ricci, M.; Baldan, A.; Lang, B.; Sarge, S.; Wang, H.; Pratt, K.; Josephs, R.; Mariassy, M.; Pfeifer, D.; Warren, J.; Bremser, W.; Ellison, S.; Toman, B.; Nelson, M.; Huang, T.; Fajgelj, A.; Gören, A.; Mackay, L.; Wielgosz, R. Methods for the SI-traceable value assignment of the purity of organic compounds (IUPAC Technical Report). Pure Appl. Chem. 2023, 95, 1–77. https://doi.org/10.1515/pac-2020-0804

12. European Medicines Agency. Guideline on quality of herbal medicinal products/traditional herbal medicinal products, Revision 3; EMA: Amsterdam, The Netherlands, 2022.

13. U.S. Food and Drug Administration. Botanical drug development: Guidance for industry; U.S. Department of Health and Human Services: Silver Spring, MD, USA, 2016.

14. Joint FAO/WHO Expert Committee on Food Additives (JECFA). Compendium of food additive specifications, 82nd meeting; FAO JECFA Monographs 19; Food and Agriculture Organization of the United Nations: Rome, Italy, 2016.

18. Piccinno, F.; Hischier, R.; Seeger, S.; Som, C. From laboratory to industrial scale: A scale-up framework for chemical processes in life cycle assessment studies. J. Clean. Prod. 2016, 135, 1085–1097. https://doi.org/10.1016/j.jclepro.2016.06.164

19. Zimmermann, A.W.; Langhorst, T.; Moni, S.; Schaidle, J.A.; Bensebaa, F.; Bardow, A. Life-cycle and techno-economic assessment of early-stage carbon capture and utilization technologies—A discussion of current challenges and best practices. Front. Clim. 2022, 4, 841907. https://doi.org/10.3389/fclim.2022.841907

20. Vadenbo, C.; Hellweg, S.; Astrup, T.F. Let's be clear(er) about substitution: A reporting framework to account for product displacement in life cycle assessment. J. Ind. Ecol. 2017, 21, 1078–1089. https://doi.org/10.1111/jiec.12519

21. Olofsson, J.; Börjesson, P. Residual biomass as resource—Life-cycle environmental impact of wastes in circular resource systems. J. Clean. Prod. 2018, 196, 997–1006. https://doi.org/10.1016/j.jclepro.2018.06.115

22. Salemdeeb, R.; zu Ermgassen, E.K.H.J.; Kim, M.H.; Balmford, A.; Al-Tabbaa, A. Environmental and health impacts of using food waste as animal feed: A comparative analysis of food waste management options. J. Clean. Prod. 2017, 140, 871–880. https://doi.org/10.1016/j.jclepro.2016.05.049

23. Ranundeniya, R.M.N.S.; Stasinopoulos, P.; Shiwakoti, N.; Lockrey, S. A critical review of methodological aspects influencing life cycle assessment results of food waste reduction strategies. J. Environ. Manag. 2025, 393, 127152. https://doi.org/10.1016/j.jenvman.2025.127152

25. Geyer, R.; Kuczenski, B.; Zink, T.; Henderson, A. Common misconceptions about recycling. J. Ind. Ecol. 2016, 20, 1010–1017. https://doi.org/10.1111/jiec.12355

26. Haupt, M.; Hellweg, S. Measuring the environmental sustainability of a circular economy. Environ. Sustain. Indic. 2019, 1–2, 100005. https://doi.org/10.1016/j.indic.2019.100005

27. Moraga, G.; Huysveld, S.; Mathieux, F.; Blengini, G.A.; Alaerts, L.; Van Acker, K.; De Meester, S.; Dewulf, J. Circular economy indicators: What do they measure? Resour. Conserv. Recycl. 2019, 146, 452–461. https://doi.org/10.1016/j.resconrec.2019.03.045

28. Zink, T.; Geyer, R. Circular economy rebound. J. Ind. Ecol. 2017, 21, 593–602. https://doi.org/10.1111/jiec.12545

38. Roosen, M.; Tonini, D.; Albizzati, P.F.; Caro, D.; Cristóbal, J.; Lase, I.S.; Ragaert, K.; Dumoulin, A.; De Meester, S. Operational framework to quantify 'quality of recycling' across different material types. Environ. Sci. Technol. 2023, 57, 13669–13680. https://doi.org/10.1021/acs.est.3c03023

40. International Organization for Standardization. ISO/TS 14076:2025 Environmental management—Environmental techno-economic assessment—Principles, requirements and guidance; ISO: Geneva, Switzerland, 2025.

45. International Organization for Standardization. ISO 14040:2006 Environmental management—Life cycle assessment—Principles and framework; ISO: Geneva, Switzerland, 2006.

46. International Organization for Standardization. ISO 14044:2006 Environmental management—Life cycle assessment—Requirements and guidelines; ISO: Geneva, Switzerland, 2006.

47. Zimmermann, A.W.; Wunderlich, J.; Müller, L.; Buchner, G.A.; Marxen, A.; Michailos, S.; Armstrong, K.; Naims, H.; McCord, S.; Styring, P.; Sick, V.; Schomäcker, R. Techno-economic assessment guidelines for CO_2 utilization. Front. Energy Res. 2020, 8, 5. https://doi.org/10.3389/fenrg.2020.00005

48. International Organization for Standardization. ISO 59020:2024 Circular economy—Measuring and assessing circularity performance; ISO: Geneva, Switzerland, 2024.

52. Vulsteke, K.; Huysveld, S.; Thomassen, G.; Rechberger, H.; Dewulf, J. Quality in the circular economy: What does it mean, and how is it measured? Resour. Conserv. Recycl. 2026, 227, 108739. https://doi.org/10.1016/j.resconrec.2025.108739
