# Supplementary material for: Laboratory-to-Industrial Translation in Plant-Derived Bioproduct Valorization: A Critical Integrative Review and Proposed Framework for Route-Level Evidence Assessment
Source: Molecules. 2026 Oct 15;31(20):3689. doi: 10.3390/molecules31203689 (PMC13707718; doi:10.3390/molecules31203689)
Supplement: Supplementary file 1 [file molecules-31-03689-s001.zip › Supplementary_Table_S3.pdf]

Supplementary Table S3. Claim-specific decision consequences in four worked cases.

Evidence package / worked case	Inference from focal metric alone	Specification-adjusted interpretation	Evidence limitations by claim path	Maximum defensible conclusion	Sources
**MI-02 Artemisinin purification**	A maceration recovery of 90.0% could be interpreted as high route-level recovery.	The source-reported conditional recoveries are displayed here as 90.0%, 87.1%, and 47.6%. Using the numerical precision recorded in Supplementary Workbook S1, the first two conditional recoveries give author-derived cumulative retention of 78.4% after chromatography, and all three give 37.3% after the complete sequence on the same initial artemisinin basis. The final value agrees, to one decimal place, with the source-reported overall recovery of 37.27%. This is an arithmetic consistency check, not independent validation. The 2.41-fold comparison between maceration-stage and final retention is author-derived.	Predeclared release criteria and repeat-batch conformity were incompletely reported. Physical resource productivity, economic plausibility, environmental improvement, and modeled substitution potential were outside the documented MI-02 package–claim scope. Observed displacement was evaluated as a separate empirical claim and remains unestablished; the available F10 review scope was insufficient for a nonreporting judgment (F10 = P).	The reported stage recoveries, author-derived intermediate retention, and final normalized target retention are supported within the stated technical boundary. They do not establish specification-conforming output. Evidence for release remains partial; the four broader claim paths were outside documented applicability, and observed displacement remains unestablished because the available F10 review scope is insufficient (P).	[60].
**FC-04 Grapefruit-peel pectin extraction**	The source reported an optimized UAHE yield of 27.34% on a mass basis that was not explicit in the accessible record; this value was 16.34% higher than that reported for CHE and could be interpreted as overall route superiority.	UAHE increased yield and reduced extraction temperature and time. Intrinsic viscosity and molecular weight were significantly lower than for CHE, while degree of esterification was slightly lower but not significantly different. The source reported greater branching and purity for UAHE pectin, although not every corresponding table-level contrast was statistically significant. Route preference therefore remains endpoint- and application-dependent.	The assessed source set did not report a predeclared commercial release specification, benchmark-matched gelation or use-level performance, route-aligned TEA or LCA, or modeled substitution potential. Observed displacement remains unestablished; the accessible material and retained F10 review documentation were insufficient to support a source-wide nonreporting judgment (F10 = P). Statistical significance should not be inferred for every property direction.	A process–property trade-off is supported; benchmark-equivalent service and general route superiority are not established.	[62].

Evidence package / worked case	Inference from focal metric alone	Specification-adjusted interpretation	Evidence limitations by claim path	Maximum defensible conclusion	Sources
**CC-05 PEF-assisted red-grape-pomace extraction**	A favorable PEF-assisted scenario could be interpreted as general economic superiority over baseline SLE.	At a modeled output of 22 kg of formulated product per batch (approximately 168 t year^{-1}), economic preference was product-price dependent: PEF-02 was favored below \$249 kg^{-1}, whereas SLE-01 was favored at \$250 kg^{-1} or above. The modeled spray-dried formulation contained maltodextrin and gum arabic, with its reported TPC percentage standardized at approximately 46%. At base product prices rounded to approximately \$233 kg^{-1}, PEF-02 lowered unit production cost by 2.72% and increased ROI by 8.11% relative to SLE-01.	Product-release evidence was partial; the modeled TPC composition does not establish a commercial grade. The source dollar notation is retained because the currency jurisdiction and monetary base year were not verified in the assessed article. The approximately \$233 kg^{-1} value is rounded from the source abstract; Table 3 of the source study gives \$232.60 kg^{-1} for SLE-01 and \$233.31 kg^{-1} for PEF-02. Route-aligned LCA, benchmark-equivalent substitution, and observed displacement were not reported in the assessed package.	A conditional economic preference is supported within the modeled product, scale, price, and configuration assumptions; environmental and displacement claims were not established.	[63].
**CC-02 Microencapsulated pomegranate-peel extract powder (PPEP)**	The deterministic Case 3 scenario at 5,000 kg day^{-1} could be interpreted as favorable overall performance from its point estimates alone, while the probabilistic result could be incorrectly attributed to the same scenario.	Among the reported modeled cases, Case 3 at 5,000 kg day^{-1} had the highest reported deterministic NPV (approximately USD 18 million) and the lowest reported LCOP (USD 7.08 kg^{-1}); its reported IRR was 53.9%. In the separate source-reported 10,000-run Monte Carlo analysis, Case 2 had the highest reported probability of positive NPV, $P(NPV > 0)$ = 91.9%. These outputs answer different economic questions. The package-level cradle-to-gate LCA identified freeze-drying as the dominant hotspot (74.7–77.3% of reported burdens; GWP 84.17 kg CO_2-eq kg^{-1} PPEP), but the assessed record did not establish attribution of that LCA to an individual TEA scale scenario.	Formal release evidence was partial. Benchmark-equivalent service, a functionally equivalent comparative basis for environmental improvement, recovered-product modeled substitution potential, and observed displacement were not established. Deterministic and probabilistic rankings must not be merged, and attribution of the package-level LCA to either Case 2 or Case 3 remains unestablished in the assessed record.	Case 3 supports the highest reported deterministic NPV and lowest LCOP, whereas Case 2 supports the highest reported probability of positive NPV, each within its model boundary. The separately scoped LCA supports hotspot identification, not comparative environmental improvement. Its attribution to an individual TEA scale scenario remains unestablished in the assessed record.	[44].

Notes: CHE, conventional heating extraction; CO_2-eq, carbon dioxide equivalent; GWP, global-warming potential; IRR, internal rate of return; LCA, life-cycle assessment; LCOP, levelized cost of production; NPV, net present value; PEF, pulsed electric field; ROI, return on investment; SLE, solid-liquid extraction; TEA, techno-economic assessment; UAHE, ultrasound-assisted heating extraction. Each row represents a case-specific evidence package. Case-specific numerical values and source-derived findings in the specification-adjusted interpretation column are linked to the case-specific source identified in the final column. Evidence limitations and maximum defensible conclusions reflect the specification-adjusted interpretation applied in this review. Publication status is a source-verification issue and is not treated as claim-specific limiting evidence.

References cited in Supplementary Table S3

44. Fajimi, L.I.; Ajala, O.O.; Silue, Y.; Oke, E.O.; Fawole, O.A. Encapsulation of pomegranate peel bioactive phenolic extract: laboratory proof-of-concept experiment, scale-up computer-aided batch process design, techno-economic feasibility and life cycle analysis. Sci. Rep. 2026, in press. https://doi.org/10.1038/s41598-026-63833-0

60. Malwade, C.R.; Qu, H.; Rong, B.-G.; Christensen, L.P. Conceptual process synthesis for recovery of natural products from plants: A case study of artemisinin from Artemisia annua. Ind. Eng. Chem. Res. 2013, 52, 7157–7169. https://doi.org/10.1021/ie302495w

62. Wang, W.; Ma, X.; Xu, Y.; Cao, Y.; Jiang, Z.; Ding, T.; Ye, X.; Liu, D. Ultrasound-assisted heating extraction of pectin from grapefruit peel: Optimization and comparison with the conventional method. Food Chem. 2015, 178, 106–114. https://doi.org/10.1016/j.foodchem.2015.01.080

63. Soltanipour, F.; Donsì, F.; Ferrari, G. Techno-economic evaluation of pulsed electric field technology in polyphenol extraction from red grape pomace. Food Bioprod. Process. 2025, 153, 185–199. https://doi.org/10.1016/j.fbp.2025.06.012
