# Supplementary material for: Laboratory-to-Industrial Translation in Plant-Derived Bioproduct Valorization: A Critical Integrative Review and Proposed Framework for Route-Level Evidence Assessment
Source: Molecules. 2026 Oct 15;31(20):3689. doi: 10.3390/molecules31203689 (PMC13707718; doi:10.3390/molecules31203689)
Supplement: Supplementary file 1 [file molecules-31-03689-s001.zip › Supplementary_Table_S4.pdf]

Supplementary Table S4 Boundary-condition evidence packages and claim-specific limitations.

Evidence package / boundary-condition case	Evidence structure and source-reported findings	Specification-adjusted interpretation	Evidence limitations by claim path	Sources
**MI-03 Steviol-glycoside recovery**	Sequential recovery from industrial stevia mother liquor. The abstract reports 42.25% crystallization recovery; Results report a crystallization ratio of 42.45 ± 0.78% at 96.89 ± 0.36% purity, with the ratio defined as crystal mass relative to starting MLS. RPC recovered 95.20% of SGs in the post-crystallization liquid at 98.08% purity; the source-calculated total was 97.23% using TR = C + (1 − C)R. The scale-up total recovery was 80.27% and is a distinct result [64].	Crystallization ratio, residual-stream RPC recovery, source-calculated total recovery, and scale-up recovery are non-interchangeable and must remain linked to their stated material basis and experiment scale.	Predeclared release criteria and repeat-batch conformity were not established. Physical resource productivity, economic plausibility, environmental improvement, and modeled substitution potential were outside the documented MI-03 package–claim scope. Observed displacement was evaluated as a separate empirical claim and remains unestablished; the available F10 review scope was insufficient for a nonreporting judgment (F10 = P).	[64].
**MI-04 Curcuminoid purification**	Combined crystallization and chromatography of CUR, DMC, and BDMC. The detailed Results report chromatography-stage yields of 81.0%, 79.7%, and 68.8% from the crystallization mother-liquor feed; the combined-process yield of pure CUR was 88.5%. Final purities were 100%, 98.6%, and 98.3%, respectively [65].	The overall CUR yield and chromatography-stage compound yields have different process bases. Each molecular target therefore requires a recovery-purity record tied to its unit-operation sequence and feed fraction.	Routine release and repeat-batch conformity were not established. Physical resource productivity, economic plausibility, environmental improvement, and modeled substitution potential were outside the documented MI-04 package–claim scope. Observed displacement was evaluated as a separate empirical claim but was not documented.	[65].
**CC-06 Pine-residue polyphenol extraction**	Comparative LCA of aqueous polyphenol extraction from pine bark, cones, and pruning residues. Among the assessed configurations, ground and sieved pine-bark powder had the lowest modeled impacts; electricity and natural gas were the principal impact drivers [66].	The LCA identifies relative burdens and process hotspots within its stated functional unit and system boundary. It does not, by itself, establish product conformity, economic plausibility, benchmark-equivalent service, or market displacement.	The assessed package did not report predeclared acceptance criteria, batch conformity, route-aligned TEA, benchmark-equivalent service, modeled substitution potential, or observed displacement.	[66].
**CC-07 Spruce-bark tannin production**	Modeled industrial-scale hot-water extraction and powder production assessed using TEA, sensitivity analysis, and LCA. The MSP was EUR 2.1 kg^{-1} and GWP was 4.5 kg CO_2-eq kg^{-1} product; overall process yield and steam demand were the dominant sensitivities [67].	The package supports conditional economic plausibility and category-specific environmental performance in parallel; neither establishes routine release, benchmark-equivalent application performance, or observed displacement.	The acceptance specification was partial, whereas specification pass and benchmark-equivalent service were not reported. TEA and LCA were explicit and support separate conditional conclusions. Modeled substitution potential and observed displacement were not reported.	[67].

Evidence package / boundary-condition case	Evidence structure and source-reported findings	Specification-adjusted interpretation	Evidence limitations by claim path	Sources
**FC-05**<br>**RG-I pectin production**	Prospective process scale-up, TEA, and cradle-to-gate LCA of RG-I pectin production. Alternative configurations lowered modeled climate-change impacts by 89.3–94.4% relative to the reference case; MSP ranged from EUR 16.2–56.0 kg^{-1} [68].	Aligned prospective models support conditional economic and environmental conclusions, but they do not establish repeat-batch release, benchmark-equivalent application performance, service-based substitution, or observed displacement.	The incomplete acceptance specification (F04 = P) was the first unresolved prerequisite for the CL05 claim. Specification-conformity and benchmark-equivalent-service evidence were not reported in the assessed record (NR). Modeled substitution potential for the recovered-pectin claim was not reported (F09 = NR), and no modeled substitution-potential conclusion is supported. TEA and LCA remained explicit within their respective model scopes. Observed displacement was assessed as a separate empirical claim but was not documented.	[55,68].
**FC-06**<br>**Kinnow-peel pectin**	Microwave-assisted acid extraction, physicochemical and functional characterization, comparison with commercial pectin, and LCA. Yield was 9.81%; EW was 833 mg, DE 66.67%, and GalA 63.15%; ethanol contributed 49% of the climate-change impact, with acetic acid also a major contributor [69].	Characterization and selected benchmark comparisons support bounded product comparability; the LCA identifies route hotspots but does not establish economic plausibility, benchmark-equivalent service, or market displacement.	Acceptance specification and specification pass were not reported. Benchmark-equivalent service was partial, TEA was outside the documented package–claim applicability map, LCA was explicit, and modeled substitution potential and observed displacement were not reported.	[55,62,69].

Notes: BDMC, bisdemethoxycurcumin; CC, composition-constrained; CO_2-eq, carbon dioxide equivalent; CUR, curcumin; DE, degree of esterification; DMC, demethoxycurcumin; EW, equivalent weight; FC, function-constrained; GalA, galacturonic acid; GWP, global-warming potential; LCA, life cycle assessment; MI, molecular-identity; MSP, minimum selling price; RG-I, rhamnogalacturonan I; SG, steviol glycoside; TEA, techno-economic assessment. Case-specific numerical values are attributed in the evidence column. The representative contextual sources are non-exhaustive and provide route- or assessment-level context; their inclusion does not imply that all cited studies belong to the same evidence package. The table does not rank routes or estimate framework performance.

References cited in Supplementary Table S4

55. Garcia-Garcia, G.; Rahimifard, S.; Matharu, A.S.; Dugmore, T.I.J. Life-cycle assessment of microwave-assisted pectin extraction at pilot scale. ACS Sustain. Chem. Eng. 2019, 7, 5167–5175. https://doi.org/10.1021/acssuschemeng.8b06052

62. Wang, W.; Ma, X.; Xu, Y.; Cao, Y.; Jiang, Z.; Ding, T.; Ye, X.; Liu, D. Ultrasound-assisted heating extraction of pectin from grapefruit peel: Optimization and comparison with the conventional method. Food Chem. 2015, 178, 106–114. https://doi.org/10.1016/j.foodchem.2015.01.080

64. Liu, Y.; Hua, X.; Zhang, M.; Zhou, A.; Zhou, X.; Yang, R. Recovery of steviol glycosides from industrial stevia by-product via crystallization and reversed-phase chromatography. Food Chem. 2021, 344, 128726. https://doi.org/10.1016/j.foodchem.2020.128726

65. Heffernan, C.; Ukrainczyk, M.; Gamidi, R.K.; Hodnett, B.K.; Rasmuson, Å.C. Extraction and purification of curcuminoids from crude curcumin by a combination of crystallization and chromatography. Org. Process Res. Dev. 2017, 21, 821–826. https://doi.org/10.1021/acs.oprd.6b00347

66. Baquero, G.; Sorolla, S.; Casas, C.; Bacardit, A. Life cycle assessment of polyphenolic extracts derived from pine by-products. Materials 2025, 18, 1000. https://doi.org/10.3390/ma18051000

67. Niiranen, I.; Forssell, S.; Oinas, P.; Dickson, R. Techno-economic and life cycle assessment of tannin production from spruce bark for leather industry applications. Green Chem. 2026, 28, 6284–6294. https://doi.org/10.1039/D6GC00120C

68. del Amo-Mateos, E.; Gosalvitr, P.; Cuéllar-Franca, R.M.; Dragone, G.; Mussatto, S.I.; García-Cubero, M.T.; Coca, M.; Lucas, S. An environmental and economic sustainability assessment of novel rhamnogalacturonan-I pectin production from agricultural residues. J. Environ. Chem. Eng. 2026, 14, 120786. https://doi.org/10.1016/j.jece.2025.120786

69. Duggal, M.; Singh, D.P.; Singh, S.; Khubber, S.; Garg, M.; Krishania, M. Microwave-assisted acid extraction of high-methoxyl kinnow (Citrus reticulata) peels pectin: Process, techno-functionality, characterization and life cycle assessment. Food Chem. Mol. Sci. 2024, 9, 100213. https://doi.org/10.1016/j.fochms.2024.100213
