# Supplementary material for: Laboratory-to-Industrial Translation in Plant-Derived Bioproduct Valorization: A Critical Integrative Review and Proposed Framework for Route-Level Evidence Assessment
Source: Molecules. 2026 Oct 15;31(20):3689. doi: 10.3390/molecules31203689 (PMC13707718; doi:10.3390/molecules31203689)
Supplement: Supplementary file 1 [file molecules-31-03689-s001.zip › Supplementary_Table_S5.pdf]

Table S5. Glossary of manuscript-specific terms and principal abbreviations.

Term / abbreviation	Meaning in this review	Definition location
Evidence package	A publication or linked set of publications organized around an identifiable route and its claim-relevant evidence. It is not an independent replicate.	2.2
Registered analytical record	A registered parent, variant, scenario, or comparison record. Its label describes organization, not statistical independence or whether all evidence is observed.	2.2; Figure S1
Route key / configuration	The feedstock condition, operations, scale, product state or grade, utility basis, and residual fate identifying the assessed route; comparison fields are added when needed.	2.2
Generated / accessible / accepted feed	Generated mass is distinguished from mass genuinely available to the route and from the accessible portion meeting incoming-feed criteria.	3.2, Eq. (1)
Gross extraction yield / target recovery	Gross extraction yield relates recovered extract mass to the stated feed-mass basis; target recovery relates recovered target mass to the target amount entering the stated operation or sequence. They are not interchangeable.	3.1–3.2
Predeclared acceptance specification	Applicable acceptance criteria fixed before outcome evaluation; a source, protocol, standard, or industrial criterion must be demonstrably applicable, not selected afterward.	2.4
Characterization / conformity / release	Characterization reports attributes; conformity requires an assessment against applicable predeclared criteria. Documented batch conformity does not alone establish routine commercial release.	2.4; 3.2
Readiness	A contextual term in discussions of product quality and translation. This review does not assign a maturity score, certification, or industrial-readiness rating.	4.1, Table 2; 5.4
Specification-conforming output	Product mass supported by both a defined final-product basis and sufficient evidence of conformity to the applicable predeclared criteria.	3.2, Eq. (4)
Benchmark-equivalent service	Service represented by the recovered product relative to a named comparator under matched test and use conditions within the supported range.	3.2, Eq. (5)
Modeled substitution potential	Scenario-conditional replacement potential based on compatible service, supply, demand, and realization assumptions.	3.2, Eq. (6)
Observed displacement	Empirical evidence linking recovered-product uptake to reduced procurement, production, or consumption of a named incumbent within a defined relationship and period.	2.5; 3.2
Claim path / stopping condition	A specified claim and its applicable requirements. An unresolved essential requirement limits that claim to its last supported conclusion, if any.	2.5; 3.4
E / P / NR / NA	Availability and completeness: explicit; partial or bounded; required but not reported in a sufficiently complete accessible record; not applicable to the documented claim. E does not mean a favorable result, and P does not automatically support a partial claim.	2.3
SR / MS	Provenance: source-reported information / source-reported modeled output. A model input or scenario setting is not itself an MS output.	2.3
SR/AD / AD	Provenance: a calculation from traceable source-reported inputs / an author derivation involving additional assumptions or constructions.	2.3

Term / abbreviation	Meaning in this review	Definition location
A / R / I	Linkage compatibility: aligned / explicitly reconciled / demonstrably incompatible in an essential basis. Missing information alone does not establish I.	2.3
NL / blank compatibility entry	NL denotes eligible evidence with no invoked cross-source or cross-basis linkage. A blank denotes no eligible evidence for compatibility assignment; neither establishes alignment.	2.3
MI / CC / FC	Molecular-identity, composition-constrained, and function-constrained product archetypes. The primary label organizes the record without removing other applicable requirements.	3.1
TEA / LCA	Techno-economic assessment / life-cycle assessment. Economic and environmental findings have separate evidence requirements even when models or inputs are shared.	2.5; 3.3
NPV / ROI / UPC	Net present value / return on investment / unit production cost. Preserve the source metric, time and monetary bases, and deterministic or probabilistic interpretation.	4.4
Internal consistency check / independent evaluation	The same-author check of records, calculations, and reporting is distinct from assessment by an independent human evaluator or external validation.	2.3; 5.5

Notes: These definitions summarize the manuscript's existing usage rather than establish new decision rules. The cited sections retain the complete conditions, exceptions, and operational definitions. A reporting code is distinct from product performance, compliance, or validation status.
