# Supplementary material for: Laboratory-to-Industrial Translation in Plant-Derived Bioproduct Valorization: A Critical Integrative Review and Proposed Framework for Route-Level Evidence Assessment
Source: Molecules. 2026 Oct 15;31(20):3689. doi: 10.3390/molecules31203689 (PMC13707718; doi:10.3390/molecules31203689)
Supplement: Supplementary file 1 [file molecules-31-03689-s001.zip › Supplementary_Table_S6.pdf]

Table S6. Descriptive distribution of recorded package–field availability and completeness codes stratified by documented access to route evidence sources.

Field	Recorded full text (n = 16)<br>E / P / NR / NA	Limited access (n = 1)<br>E / P / NR / NA	All packages (n = 17)<br>E / P / NR / NA
F01 Feedstock identity	16 / 0 / 0 / 0	1 / 0 / 0 / 0	17 / 0 / 0 / 0
F02 Route configuration	16 / 0 / 0 / 0	1 / 0 / 0 / 0	17 / 0 / 0 / 0
F03 Stage recovery	3 / 13 / 0 / 0	0 / 1 / 0 / 0	3 / 14 / 0 / 0
F04 Acceptance specification	1 / 9 / 6 / 0	0 / 1 / 0 / 0	1 / 10 / 6 / 0
F05 Specification-conformity evidence	1 / 8 / 7 / 0	0 / 1 / 0 / 0	1 / 9 / 7 / 0
F06 Benchmark-equivalent service	0 / 3 / 10 / 3	0 / 0 / 0 / 1	0 / 3 / 10 / 4
F07 TEA	5 / 0 / 5 / 6	0 / 0 / 0 / 1	5 / 0 / 5 / 7
F08 LCA	11 / 0 / 1 / 4	0 / 0 / 0 / 1	11 / 0 / 1 / 5
F09 Modeled substitution potential	0 / 0 / 13 / 3	0 / 0 / 0 / 1	0 / 0 / 13 / 4
F10 Observed displacement	0 / 4 / 12 / 0	0 / 1 / 0 / 0	0 / 5 / 12 / 0

Notes: Values are counts in the order E / P / NR / NA. For each field, E + P + NR equals stratum size minus NA. Codes retain the definitions in Section 2.3. The 170 cells are nested within 17 packages and are not independent observations. The recorded full-text stratum includes 15 sources recorded as full text checked and the publisher-hosted unedited source for CC-02; accepted manuscripts are not reclassified as final published versions. MI-01 is the only package in the limited-access stratum. Source-access strata preserve the recorded access history and do not establish the adequacy of field-specific review. MI-02, MI-03, CC-03, and FC-04 remain in the recorded full-text stratum, but their F10 codes are P because the accessible material and retained review records were insufficient for a complete F10 nonreporting judgment. Together with MI-01, these five F10 P codes reflect access or review-scope limitations, not partial empirical displacement. MI-01's F03–F05 retain their existing incomplete-evidence rationales. Access groups follow the route-source identifiers in the coding records; general contextual and exclusion-audit sources are excluded. All packages have one route-source identifier, with no mixed-access package. This table reports the finalized codes after the four F10 revisions and the reclassification of CC-04 F09 from P to NR; it neither estimates an effect of access nor removes selection bias or establishes independent validation. The following mapping identifies the recorded access and final F10 code for each package.

Table S6 (continued). Package-to-source mapping used for access stratification

Package	Route source	Recorded access / version	F10
MI-01	SRC-029	Limited: official abstract and metadata	P
MI-02	SRC-015	Full text checked; version limits retained	P
MI-03	SRC-018	Full text checked; version limits retained	P
MI-04	SRC-019	Full text checked; version limits retained	NR
CC-01	SRC-013	Full text checked; version limits retained	NR
CC-02	SRC-014	Publisher-hosted unedited full text	NR
CC-03	SRC-027	Full text checked; version limits retained	P
CC-04	SRC-028	Full text checked; version limits retained	NR
CC-05	SRC-017	Full text checked; version limits retained	NR

Package	Route source	Recorded access / version	F10
CC-06	SRC-020	Full text checked; version limits retained	NR
CC-07	SRC-021	Full text checked; version limits retained	NR
FC-01	SRC-024	Full text checked; version limits retained	NR
FC-02	SRC-025	Full text checked; version limits retained	NR
FC-03	SRC-026	Full text checked; version limits retained	NR
FC-04	SRC-016	Full text checked; version limits retained	P
FC-05	SRC-022	Full text checked; version limits retained	NR
FC-06	SRC-023	Full text checked; version limits retained	NR

Source: Supplementary Workbook S1, 02_Source_Register. (Access_level, column I), with source IDs cross-checked in 04_Configuration_Index., 07_Cell_Decision_Master., 08_Evidence_Record_Log., and 14_Claim_Path_Stop_Audit. The associated source register retains individual access notes and manuscript versions.
